# The General Factor of Psychopathology (p): Choosing Among Competing Models and Interpreting p

**DOI:** 10.1177/21677026221147872

**Published:** 2023-05-03

**Authors:** Avshalom Caspi, Renate M. Houts, Helen L. Fisher, Andrea Danese, Terrie E. Moffitt

**Affiliations:** 1 Department of Psychology & Neuroscience, Duke University; 2 PROMENTA, Department of Psychology, University of Oslo; 3 Social, Genetic & Developmental Psychiatry Centre, Institute of Psychiatry, Psychology, & Neuroscience, King’s College London; 4 ESRC Centre for Society and Mental Health, Kings’ College London; 5 Department of Child and Adolescent Psychiatry, Institute of Psychiatry, Psychology, & Neuroscience, King’s College London; 6 National and Specialist CAMHS Clinic for Trauma, Anxiety, and Depression, South London and Maudsley NHS Foundation Trust, London, United Kingdom

**Keywords:** developmental psychopathology, diagnosis, p, structure of psychopathology

## Abstract

Over the past 10 years, the general factor of psychopathology, p, has attracted interest and scrutiny. We review the history of the idea that all mental disorders share something in common, p; how we arrived at this idea; and how it became conflated with a statistical representation, the bifactor model. We then leverage data from the Environmental Risk Longitudinal Twin Study to examine the properties and nomological network of different statistical representations of p. We found that p performed similarly regardless of how it was modeled, suggesting that if the sample and content are the same, the resulting p factor will be similar. We suggest that the meaning of p is not to be found by dueling over statistical models but by conducting well-specified criterion-validation studies and developing new measurement approaches. We outline new directions to refresh research efforts to uncover what all mental disorders have in common.

Mental disorders that are thought to be distinct tend to co-occur. *Concurrently*, people who present with one mental disorder are more likely to meet diagnostic criteria for many other different disorders—the phenomenon known as comorbidity (de Jonge et al., 2018; Kessler et al., 2005). *Across the life course*, people who experience one mental disorder are at increased risk of previously or subsequently having all other disorders (Caspi et al. 2020; Plana-Rippol et al. 2019). And *across generations*, parents who experience one mental disorder are at increased risk of having offspring who will experience many other different disorders (Caspi et al., 2014). This ubiquitous overlap between different mental disorders has given rise to the suggestion that there may be one propensity to developing any and all forms of mental disorders. The idea of a general factor of psychopathology (Lahey et al., 2012), often called “p” (Caspi et al., 2014), has attracted interest and curiosity but also suspicion and even derision. We have three goals in this article. The first is to provide a historical record of how statistical approaches to modeling the structure of psychopathology came to dominate research and discussion about the idea of p. The second goal is to report an empirical evaluation of outstanding questions about modeling the higher-order structure of psychopathology and p. And the third goal is to offer testable ideas for future research about the nature of p.

## Ascendant Methodological Approaches to Modeling the Structure of Psychopathology: A Personal Prologue

Our story begins in 1995. Working with mental-disorder data from a new age-21 wave of the Dunedin Longitudinal Study, we observed remarkably high rates of comorbidity (Newman et al., 1998). Other studies, including the influential new U.S. National Comorbidity Survey, were hailing comorbidity, too (Caron & Rutter, 1991; Kessler et al., 1994). Most people who met diagnostic criteria for one disorder (e.g., depression) also met diagnostic criteria for another disorder (e.g., anxiety). Despite this emerging knowledge, the prevailing reflex when studying a specific disorder, such as depression, was to exclude participants with comorbid conditions or to control statistically for disorders other than depression. Scholars followed this practice in hundreds of publications. But if mental disorders were so highly correlated/comorbid, did research run the risk of misrepresenting mental disorders by studying such “partialed” or “pure” versions of a disorder? Evidence emerged that people with comorbid diagnoses constituted the most serious cases with the worst prognosis, which suggested that excluding comorbid cases was limiting research to the least consequential cases of a disorder (Kessler et al., 2005; Wittchen, Lieb, et al., 1999). There had to be a different way to think about comorbidity other than as a statistical nuisance. Psychological scientists noted the need for research that would deliberately examine patterns of comorbidity to “elucidate the broad, higher-order structure of phenotypic psychopathology” (L. A. Clark et al., 1995, p. 131).

In thinking about this problem, we drew on our background in child development research and speculated that dimensional models that had informed research on child psychopathology (Achenbach & Edelbrock, 1981) could also inform research on adult psychopathology. This speculation found a methodological match in structural equation modeling, still relatively new at the time. It offered an opportunity to use confirmatory factor analysis in the Dunedin Study to evaluate alternative hypotheses about the latent structure underlying 10 common adult mental disorders.

Our initial effort to model the structure of adult psychopathology tested a correlated-factors model in which some disorders were presumed to reflect internalizing problems (e.g., depression, generalized anxiety, phobias) and others were presumed to reflect externalizing problems (conduct disorder, substance dependence), as they did in children (Krueger et al., 1998). This suggested that comorbidity may occur because common mental disorders are reliable, covariant indicators of two stable, underlying core psychopathological processes. This idea, along with the use of the correlated-factors model, was then imported to the National Comorbidity Survey data, where it replicated (Krueger, 1999). Although both the statistical analyses and interpretation met with resistance (Wittchen, Hofler, & Merikangas, 1999), the idea that different mental disorders could be understood as manifestations of latent factors gained momentum with implications for etiology (Forbes et al., 2016; Krueger & Markon, 2006). As one example of how this work has unfolded over the past 20 years, it sparked research about the shared genetic etiology of different externalizing disorders (Kendler et al., 2003; Krueger et al., 2002), culminating in the discovery of genetic variants associated with a wide range of externalizing phenotypes (Karlsson Linnér et al., 2021).

Fifteen years on, with the age-38 assessment of the Dunedin Study in 2011, we revisited the structure of psychopathology. Two unanswered questions beckoned. First, where do thought disorders fit in the structure of psychopathology? Research on the structure of psychopathology had been focused on internalizing and externalizing disorders, partly because thought disorders were not part of the legacy of childhood-psychopathology research—where the two higher-order dimensions were first described—and partly because most surveys of mental disorders did not collect data on psychotic thought symptoms. We had been improving our assessment of psychotic symptoms in the Dunedin Study (Cannon et al., 2002; Poulton et al., 2000), prompted by new research revealing that psychotic symptoms are more commonly experienced in the general population than previously assumed (van Os et al., 2009). If researchers only ask, psychotic symptoms are out there. We asked, and then we sought to incorporate the resulting psychotic symptoms in our data into an expanded model of the structure of psychopathology (Kotov, Chang, et al., 2011; Kotov, Ruggero, et al., 2011; Wright et al., 2013).

This led to a second question: When there had been only externalizing and internalizing disorders, the focus was on how they differed, but the emergence of three correlated factors now drew attention to how much variation they all shared. How should we interpret the high correlations (≈.5–.8) between the factors in the correlated-factors model? Could these correlations between factors come about because the factors are influenced by common causation? In fact, our initial work on the Dunedin Study in the 1990s, using data from ages 18 and 21 years, had tested and rejected a one-factor model of psychopathology (Krueger et al., 1998; see also Krueger, 1999). With Dunedin data now spanning 38 years, we revisited the one-factor idea using a higher-order model, but we encountered convergence problems (specifically, a linear dependency leading to an unidentified model). As we vacillated, Lahey et al. (2012) independently fitted a bifactor model to diagnostic data about depression, anxiety, fears, substance-use disorders, and antisocial personality, reporting that a general psychopathology factor fitted their data well. We had a head-slapping moment; “why didn’t we think of that?” We subsequently fitted a bifactor model to our data (Caspi et al., 2014), and we called the general psychopathology factor “p.” The name p was a nod to g in research on intelligence. g summarizes the observation that individuals who do well on one type of cognitive test tend to do well on all other types of cognitive tests, suggesting that all cognitive functions—whether verbal skills, visuospatial skills, working memory, processing speed, or other skills—are, to some extent, influenced by common etiology (Deary, 2001). Perhaps all mental disorders were likewise influenced by common etiology to some extent.

## Methodological and Conceptual Debates

Unfortunately, the statistical bifactor model and the conceptual idea of p became conflated. The conflation has mired research about p in debates about factor analysis and sown confusion about the meaning of the resulting psychopathology dimensions.

The first debate focuses on how best to model a general factor of psychopathology (Bornakalova et al., 2020; Brunner et al., 2012; Rindskopf & Rose, 1988). There are several alternatives (see Fig. S1 in the Supplemental Material available online). In the *one-factor model*, every symptom/disorder loads directly on only one factor. In the *higher-order model*, every symptom/disorder loads on one of several correlated factors. These correlated factors (e.g., Externalizing, Internalizing, Thought Disorders) constitute a *correlated-factors model*. The correlated first-order factors then load on a second-order factor, the general factor of psychopathology, or p, that is defined by the covariation of the first-order factors. In contrast, in the *bifactor model*, every symptom/disorder loads on both a general factor (p) and also on a factor that is specified to be uncorrelated with the general factor, that is, a factor free of p, hereafter termed “p-free factors.” Does it matter how p is modeled?

A second, related debate involves the use of fit statistics to adjudicate between different representations of the structure of psychopathology. It has been noted that the goodness of fit of bifactor models tends to be positively biased. The implication is that previous claims that the bifactor model is superior to other models, made on the basis of goodness-of-fit indices, are suspect. Ancillary measures should be consulted when comparing different models (e.g., Bader & Moshagen, 2022; Bonifay & Cai, 2017; Greene et al., 2019; Murray & Johnson, 2013; Sellbom & Tellegen, 2019). These concerns raise a question: Has the bifactor model misled researchers about the existence of p?

A third debate centers on variations that have been introduced to the bifactor model. For example, some analyses have assumed that symptoms/disorders share additional sources of common variance other than p and have allowed the p-free factors to correlate with each other rather than constrain them to be uncorrelated. Other analyses have estimated bifactor models in which certain symptoms/disorders measure only the general factor but not any p-free factor (Eid et al., 2017; Heinrich et al., 2021). Do such modeling decisions alter the meaning of p?

Alongside attention to the bifactor model’s general factor of psychopathology, a fourth debate concerns how to evaluate the p-free factors in the bifactor model (Lahey et al., 2021; Moore et al., 2020). Before we turn to this question, it is important to agree about terminology. We use the term “first-order factors” to refer to factors (e.g., Externalizing, Internalizing, Thought problems) that group correlated symptoms/disorders in the correlated-factors model. The meaning of the correlated factors is not in dispute; they reflect the multidimensionality of mental disorders and have done so for decades. We use the term “p-free factors” to refer to factors in the bifactor solution that model the residual variance of each symptom/disorder beyond variance accounted for by the general factor of psychopathology. These p-free factors are often given the same labels (e.g., Externalizing, Internalizing, Thought problems) as the first-order factors. This practice has sown confusion. We suggest calling them p-free Externalizing, Internalizing, and Thought dimensions. The meaning of the p-free factors is dubious. Do they capture mental disorder that is not shared with any other disorders or p? Do they reflect individual differences in thoughts, feelings, and behaviors that do not involve harmful dysfunction? Are they noise? Do they have any robust, meaningful correlates?

And finally, there is the issue of “What is p?” The idea is that this is a latent manifestation of some shared causal factor (or factors) associated with every symptom/disorder. But if the structural validity of p differs as a function of the statistical model from which it is extracted, the very idea is not sound.

## Empirical Tests

To address these debates, we turned to data from the Environmental Risk (E-Risk) Longitudinal Twin Study, a nationally representative sample that follows 2,232 twins across the first two decades of life. We used data from E-Risk because the study (a) has measured a wide range of externalizing, internalizing, and thought disorder symptoms that can be used to estimate and compare different structural models of psychopathology; (b) provides an opportunity to estimate genetic and environmental contributions to psychopathology; and (c) contains longitudinal-developmental information about some of the most potent risk factors for mental disorders starting in the early years of life and that can be used to evaluate the nomological network of different representations of the structure of psychopathology (Garber & Strassberg, 1991).

Our empirical work is presented in five parts. First, we evaluate the fit of different statistical models of the structure of psychopathology using both traditional and ancillary fit statistics. Second, we compare factor scores derived from different statistical models of psychopathology: correlated-factors, one-factor, higher-order, and bifactor models. Third, we apply E-Risk’s twin design to examine genetic and environmental influences on psychopathology factor scores taken from different statistical models. Fourth, we use E-Risk’s longitudinal-developmental data to evaluate risk factors and correlates of psychopathology, including family history of mental illness, socioeconomic deprivation, early-emerging cognitive and self-regulation difficulties, adverse experiences in childhood and adolescence, and inflammation. Fifth, we evaluate the meaning of p-free factors extracted from bifactor models.

To help the reader anticipate where this article is heading, here is a summary of what the data show: p performed similarly regardless of how it was modeled, and caution is warranted when interpreting p-free factors yielded by bifactor models. We conclude that the answer to the meaning of p will not be found in continued dueling over statistical models, but rather it will be found in well-specified criterion validation studies and new measurement development.

## Method

### Study sample

Participants were members of the E-Risk Longitudinal Twin Study, which tracks the development of a birth cohort of 2,232 children born in 1994 and 1995 across England and Wales (Moffitt & E-Risk Study Team, 2002). In brief, the E-Risk sample was constructed in 1999–2000, when 1,116 families (93% of those eligible) with same-sex 5-year-old twins participated in home-visit assessments. This sample contained 56% monozygotic and 44% dizygotic twin pairs; sex was evenly distributed within zygosity (49% male). Of the full sample, 7% self-identified as Black, Asian, or mixed race. Families were recruited to represent the UK population with newborns in the 1990s on the basis of maternal age and geographic location both to ensure adequate numbers of children in disadvantaged homes and to avoid an excess of twins born to well-educated women using assisted reproduction. The study sample represents the full range of socioeconomic conditions in Great Britain, as reflected in the families’ distribution on a neighborhood-level socioeconomic index (Acorn, or “a classification of residential neighborhoods,” developed by CACI for commercial use; Odgers et al., 2012): 25.6% of E-Risk families live in “wealthy achiever” neighborhoods compared with 25.3% nationwide, 5.3% live in “urban prosperity” neighborhoods (vs. 11.6% nationwide), 29.6% live in “comfortably off” neighborhoods (vs. 26.9% nationwide), 13.4% live in “moderate means” neighborhoods (vs. 13.9% nationwide), and 26.1% live in “hard-pressed” neighborhoods (vs. 20.7% nationwide). E-Risk underrepresents urban prosperity neighborhoods because such households are likely to be childless.

Follow-up home visits were conducted when participants were ages 7 (98% participation), 10 (96% participation), 12 (96% participation), and most recently, 18 (93% participation) years. At age 18 years, 2,066 participants were assessed, each twin by a different interviewer. The average age at the time of assessment was 18.4 years (*SD* = 0.36); all interviews were conducted after the 18th birthday. There were no differences between individuals who did and did not take part at age 18 years in terms of socioeconomic status (SES) assessed when the cohort was initially defined, χ^2^ (2, *N* = 2232) = 0.86, *p* = .65; age-5 IQ scores, *t* (1112) = 0.98, *p* = .33; age-5 internalizing or externalizing behavior problems, *t* (1115) = 0.40, *p* = .69, and *t* (1115) = 0.41, *p* = .68, respectively; or childhood poly-victimization, *z* = 0.51, *p* = .61. The Joint South London, Maudsley, and Institute of Psychiatry Research Ethics Committee approved each phase of the study. Parents gave written informed consent; twins gave assent between 5 and 12 years old and then written informed consent at age 18 years. Data reported in this article are not publicly available because of lack of informed consent and ethical approval for public data sharing. Information about data access is available at https://sites.duke.edu/moffittcaspiprojects/data-use-guidelines/. Syntax and output for all models are available in the Supplemental Material and at https://moffittcaspi.trinity.duke.edu/research-topics/statistical-code.

### Measures

#### Symptoms of mental disorders

At age 18, E-Risk members were assessed in private interviews about symptoms of mental disorders (Table S1 in the Supplemental Material), as previously described (Schaefer et al., 2018). We assessed past-year symptoms of five externalizing-spectrum disorders: *Diagnostic and Statistical Manual of Mental Disorders* (*DSM*; American Psychiatric Association, 1994, 2013) symptoms of conduct disorder, attention-deficit/hyperactivity disorder (ADHD), alcohol dependence, and cannabis dependence, as well as symptoms of tobacco dependence, which were assessed with the Fagerström Test for Nicotine Dependence (Heatherton et al., 1991). We assessed past-year symptoms of four internalizing-spectrum disorders: *DSM* (American Psychiatric Association, 1994, 2013) symptoms of depression, generalized anxiety disorder (GAD), and posttraumatic stress disorder (PTSD), as well as symptoms of eating disorder, which were assessed via the SCOFF questionnaire (Morgan et al., 1999). We assessed symptoms of thought disorders in two ways. First, each E-Risk member was interviewed about delusions and hallucinations (e.g., “Have you ever thought you were being followed or spied on?” “Have you ever heard voices that other people cannot hear?”). This interview had also been administered at an earlier age to E-Risk members, and its scoring system is described in detail elsewhere (Polanczyk et al., 2010). Second, each E-Risk member was asked about unusual thoughts and feelings (e.g., “My thinking is unusual or frightening,” “People or places I know seem different”), drawing on item pools since formalized in prodromal psychosis instruments, including the PRIME screen and Structured Interview for Prodromal Syndromes (SIPS; Loewy et al., 2011). The six prodromal psychosis symptoms were used to create two parcels so that a Thought Disorders factor could be identified with three indicators (i.e., psychosis symptoms, prodromal symptoms A, and prodromal symptoms B).

#### Nomological network of psychopathology

To evaluate the nomological network of psychopathology, we examined the following putative causes of mental disorders: family history of mental illness, socioeconomic deprivation, early-emerging cognitive and self-regulation difficulties, adverse experiences in childhood and in adolescence, and inflammation.

##### Family history of psychiatric disorder

Family history of psychiatric disorder was ascertained at the age-12 assessment through a family-history interview with biological parents (Milne et al., 2008; Weissman et al., 2000). Family history of psychiatric disorder was defined as a report of (a) treatment or hospitalization for a psychiatric disorder or substance use problem or (b) attempted or completed suicide for any of the child’s biological mother, father, grandparents, or aunts and uncles. We report the proportion of family members with any of these conditions (Milne et al., 2008).

##### Childhood SES

The family SES when the twins were 5 years of age was defined through a standardized composite of parental income, education, and occupation. The three SES indicators were highly correlated (*r*s = .57–.67) and loaded significantly onto one latent factor (Trzesniewski et al., 2006).

##### Children’s IQ

At age 5, children’s IQ was individually tested using a short form of the Wechsler Preschool and Primary Scale of Intelligence–Revised (Wechsler, 1990). Two subtests (Vocabulary and Block Design) were used to prorate children’s IQs following procedures described by Sattler (1992, pp. 998–1004).

##### Low self-control

Children’s self-control during their first decade of life was measured using a multi-occasion/multi-informant strategy. A self-control factor was estimated via nine measures, including observational ratings of children’s lack of control (age 5 years), parent and teacher reports of poor impulse control (ages 5, 7, and 10 years), self-reports of inattentive and impulsive behavior (age 7 years), and interviewer judgments of the personality trait of conscientiousness (age 10 years; Richmond-Rakerd et al., 2019).

##### Childhood maltreatment

These measures have been described previously (Danese et al., 2017). In brief, mothers reported on their children’s exposure to several types of maltreatment during a standardized clinical interview when the children were 5, 7, 10, and 12 years of age. These reports were supplemented by researchers’ observations of indications of abuse and neglect at any of the successive home visits, information from clinicians whenever the study team made a child-protection referral, and children’s self-reports of bullying. Exposures assessed included domestic violence between the mother and her partner, frequent bullying by peers, physical maltreatment by an adult, sexual abuse, emotional abuse and neglect, and physical neglect. Exposure to each type of victimization was coded on a 3-point scale, in which 0 indicated no exposure, 1 indicated probable or less severe exposure, and 2 indicated definite or severe exposure.

##### Adolescent victimization

These measures have been described previously (Fisher et al., 2015). In brief, participants were interviewed at age 18 about exposure to a range of adverse experiences between 12 and 18 years using the second revision of the Juvenile Victimization Questionnaire (JVQ; Finkelhor et al., 2011; Hamby et al., 2004), adapted as a clinical interview. Each co-twin was interviewed by a different research worker, and each JVQ question was asked for the period since age 12. Age 12 is a salient age for our participants because it is when British children leave primary school to enter secondary school. Our adapted JVQ comprised 45 questions covering seven different forms of victimization: maltreatment, neglect, sexual victimization, family violence, peer/sibling victimization, cyber victimization, and crime victimization. Like childhood maltreatment, exposure to each type of adolescent victimization was also coded on a 3-point scale, in which 0 indicated no exposure, 1 indicated less severe exposure, and 2 indicated severe exposure.

##### Inflammatory biomarker (soluble urokinase plasminogen activator receptor)

Systemic chronic low-grade inflammation plays a role in the progression of many diseases. Mental health clinicians and researchers are also fired up about inflammation, although it is unclear whether inflammation contributes to the pathophysiology of mental disorders or is a by-product of mental disorders (Bauer & Teixeira, 2019). A challenge in studying the association between mental disorders and inflammation is the measurement of systemic inflammation. A variety of biomarkers are used to assess systemic inflammation, but there is limited consensus about which measures are optimal (Furman et al., 2019). Commonly used inflammatory biomarkers, such as C-reactive protein or inteleukin-6, can reflect acute change in immune activity, in addition to systemic chronic inflammation. A newer biomarker of systemic chronic inflammation is soluble urokinase plasminogen activator receptor (suPAR), which is less sensitive to acute changes in health compared with other inflammatory biomarkers and could therefore be a better measure of systemic chronic inflammation (Rasmussen et al., 2021).

Venous blood was collected from 82% (*n* = 1,700) of E-Risk participants in EDTA tubes at age 18. Tubes were spun at 2,500 × g for 10 min, and plasma was drawn off. Samples were stored at −80° C. Plasma was available for 1,448 participants. Plasma suPAR was analyzed with the suPARnostic AUTO Flex ELISA (ViroGates A/S, Birkerød, Denmark) following the manufacturer’s protocol. The coefficient of variation was 6% (Rasmussen et al., 2019).

## Results

The first two sections below will compare different statistical models of the structure of psychopathology and test the similarity of factor scores derived from these models. The following three sections will evaluate risk factors and correlates of psychopathology against factor scores derived from different statistical models of psychopathology. Collectively, these sections are intended to help answer the question of whether p is robust to how it is modeled.

### Evaluating the fit of different statistical models of the structure of psychopathology

Using confirmatory factor analysis, we tested four models (Brunner et al., 2012; Rindskopf & Rose, 1988) that are frequently used to examine hierarchically structured constructs (Figs. S1A–S1D in the Supplemental Material): (a) a correlated-factors model with three factors (representing Externalizing, Internalizing, and Thought Disorders), (b) a one-factor model of general psychopathology (labeled “p”), (c) a higher-order factor model (representing Externalizing, Internalizing, and Thought Disorders, which each load on the general factor of psychopathology, p), and (d) a bifactor model specifying a general psychopathology factor (p) and three orthogonal p-free factors for Externalizing, Internalizing, and Thought Disorders. All models included the 12 symptom-count variables (i.e., alcohol dependence, cannabis dependence, tobacco dependence, conduct disorder, ADHD, anxiety, depression, eating disorders, PTSD, psychosis symptoms, prodromal symptoms A, prodromal symptoms B).

In confirmatory factor analysis, latent continuous factors are hypothesized to account for the pattern of covariance among observed variables. As expected, all symptom scales were positively correlated with each other, averaging .23 (*Mdn* = 0.21) and ranging from .04 to .50 (Table S2 in the Supplemental Material). Higher correlations between some disorders (but not others) support the construction of latent factor scores representing the Externalizing, Internalizing, and Thought Disorders dimensions, whereas positive correlations between all symptom scales support the construction of a higher-order factor of general psychopathology (p).

All confirmatory factor analyses were run as two-level clustered models to account for the nesting of twins within families. We used *Mplus* (Version 8.5; Muthén & Muthén, 2017) and the robust maximum likelihood estimator, which uses a sandwich estimator to provide standard errors that are robust to nonnormality and nonindependence of observations. We assessed the fit of each model using the root-mean-square error of approximation (RMSEA; close fit < 0.05, reasonable fit < 0.08), comparative fit index (CFI; good fit > 0.90), Tucker-Lewis index (TLI; good fit > 0.90), and standardized root-mean-square residual (SRMR; good fit < 0.08). We also examined relative fit using the Akaike information criterion (AIC), Bayesian information criterion (BIC), and sample-adjusted BIC, for all of which lower scores indicate better fit.

#### Comparing traditional fit statistics in four competing models of psychopathology

##### Correlated-factors model

Our first model, the correlated-factors model (Fig. S1A), tested the hypothesis that there are correlated dimensions, each of which influences a subset of the measured disorder symptoms. In our case, we tested three dimensions representing Externalizing (with loadings from ADHD, alcohol dependence, cannabis dependence, tobacco dependence, and conduct disorder), Internalizing (with loadings from GAD, major depressive episode [MDE], eating pathology, and PTSD), and Thought Disorders (with loadings from psychosis symptoms and prodromal symptoms). The model assumes that the Externalizing, Internalizing, and Thought Disorders dimensions may be correlated.

Table S3A in the Supplemental Material shows the fit statistics, standardized factor loadings, and correlations between factors for this model. Absolute model-fit statistics were as follows: RMSEA = 0.04 (90% confidence interval [CI] = [0.04, 0.05]), CFI = 0.92, TLI = 0.90, and SRMR = 0.04, indicating good model-to-data fit. The relative model-fit statistics were as follows: AIC = 85,787.17, BIC = 86,006.87, and sample-adjusted BIC = 85,882.97. Loadings on each of the three factors were all positive, generally high (all *p*s < .001), and averaged 0.58 (Externalizing: average loading = 0.55; Internalizing: average loading = 0.57; Thought Disorders: average loading = 0.62). In the correlated-factors model, correlations between the three factors were all positive and ranged from .48 between Externalizing and Thought Disorders to .69 between Internalizing and Thought Disorders.

##### One-factor model

Our second model, a one-factor model (Fig. S1B), tested the hypothesis that there is one general factor, p, that influences all of the measured diagnoses or symptoms. Table S3A shows the fit statistics and standardized factor loadings for this model. Absolute model-fit statistics were as follows: RMSEA = 0.07 (90% CI = [0.07, 0.08]), CFI = 0.75, TLI = 0.69, and SRMR = 0.07, indicating mixed to poor model-to-data fit. The relative model-fit statistics were as follows: AIC = 86,615.04, BIC = 86,817.85, and sample-adjusted BIC = 86,703.47. Loadings on the one-factor model of p were all positive, generally high (all *p*s < .001), and averaged 0.48 (range = 0.34–0.63).

##### Higher-order factor model

Our third model, the higher-order factor model (Fig. S1C), is isomorphic with the correlated-factors model, meaning that it is a different manifestation of the same model. The higher-order factor model includes the same dimensional factors for Externalizing, Internalizing, and Thought Disorders, but rather than specifying them as correlated, it has them loading on the general factor, p.

Table S3A shows the fit statistics and standardized factor loadings for this model. Absolute model-fit statistics were as follows: RMSEA = 0.04 (90% CI = [0.04, 0.05]), CFI = 0.92, TLI = 0.90, and SRMR = 0.04, indicating good model-to-data fit. The relative model-fit statistics were as follows: AIC = 85,787.17, BIC = 86,006.87, and sample-adjusted BIC = 85,882.97. Loadings on each of the three factors were all positive, generally high (all *p*s < .001), and averaged 0.58 (Externalizing: average loading = 0.55; Internalizing: average loading = 0.57; Thought Disorders: average loading = 0.62). Externalizing loaded on p at 0.61, Internalizing loaded at 0.88, and Thought Disorders loaded at 0.79.

##### Bifactor model with orthogonal p-free factors

Our fourth model, the bifactor model (Fig. S1D), tested the hypothesis that the symptom measures reflect both general psychopathology and narrower p-free dimensions. In this model, p is represented by a factor that directly influences all of the symptom measures, and the p-free dimensions are represented by orthogonal factors (i.e., Externalizing, Internalizing, and Thought Disorders), each of which influences a smaller subset of the symptom items. For example, symptoms of alcohol dependence load jointly on p and on a p-free factor, whereas symptoms of depression load jointly on p and on a different, orthogonal p-free factor. The p-free factors represent the constructs of Externalizing, Internalizing, and Thought Disorders that share variance apart from p. The classic bifactor (orthogonal p-free) model assumes that the p-free factors are uncorrelated (Yung et al., 1999), and we specified this model as such.

Table S3A shows fits statistics and standardized factor loadings for this bifactor (orthogonal p-free) model. Absolute model-fit statistics were as follows: RMSEA = 0.04 (90% CI = [0.03, 0.05]), CFI = 0.94, TLI = 0.91, and SRMR = 0.03, indicating good model-to-data fit. The relative model-fit statistics were as follows: AIC = 85,660.95, BIC = 85,931.35, and sample-adjusted BIC = 85,778.85. Loadings on the general factor, p, were all positive, generally high (all *p*s < .001), and averaged 0.43 (range = 0.25–0.65); the highest standardized loadings were for MDE (0.65), prodromal symptoms (0.56 and 0.54), GAD (0.50), and ADHD (0.50). Loadings for the p-free factors were all positive and averaged 0.44 for Externalizing and 0.42 for Thought Disorder. However, all loadings on the p-free Internalizing factor were lower (average 0.23) and nonsignificant (*p* > .05), suggesting that, in this cohort, there were no p-free Internalizing symptoms.

##### Summary

Looking at traditional model-fit statistics, we can see that the one-factor model has the poorest fit and that fits are successively better for the correlated-factors and higher-order factor models. The bifactor (orthogonal p-free) model appears to fit best.

#### Alternative specifications of the bifactor model

As the bifactor model has gained currency in research about the structure of psychopathology, some analyses have introduced variations in modeling the p-free factors. We evaluate these variations next because there has been concern that modeling variations may have a large influence on the meaning on p.

##### Bifactor model with oblique p-free factors

The bifactor (oblique p-free) model assumes that there are additional sources of common variance other than p, and thus allows the p-free factors to correlate (Fig. S1E in the Supplemental Material). Traditional fit statistics for this model indicate that this was the best-fitting model both in terms of absolute fit measures, RMSEA = 0.03 (90% CI = [0.02, 0.04]), CFI = 0.97, TLI = 0.95, and SRMR = 0.03, and relative model-fit statistics, AIC = 85,574.16, BIC = 85,861.46, and sample-adjusted BIC = 85,699.43 (Table S3B in the Supplemental Material). When compared with the bifactor (orthogonal p-free) model, factor loadings for Externalizing symptoms on p increased from an average of 0.34 to an average of 0.52, factor loadings for Internalizing symptoms on p decreased from an average of 0.53 to an average of 0.35, and factor loadings for Thought Disorders symptoms decreased from an average of 0.46 to an average of 0.29. These findings suggests that in this model, p is more heavily weighted toward externalizing symptomatology than in the original bifactor (orthogonal p-free) model.

##### Bifactor-1 p-free factor models

The next three alternative bifactor specifications each drop one of the three p-free factors in turn (Figs. S1F–S1H in the Supplemental Material). These models conceptualize p relative to a reference domain of symptoms that are not included in the model as a separate p-free factor. In these models, p is defined by the omitted domain and that portion of variance that other symptoms share with items in the omitted domain; the remaining p-free factors represent variance in symptoms that is not shared with the reference domain. Thus, the interpretation of both p and the p-free factors may change depending on which p-free factor is omitted and designated as the reference (Eid et al., 2017; Heinrich et al., 2021).

Omitting the Externalizing p-free factor resulted in a poorly fitting model using traditional absolute fit statistics, RMSEA = 0.06 (90% CI = [0.05, 0.06]), CFI = 0.87, TLI = 0.82 (Table S3B). As would be expected, and as indicated by the factor loadings, p in the bifactor (–p-free Externalizing) model was more heavily weighted toward externalizing symptomatology than in the original bifactor (orthogonal p-free) model. Removing the Internalizing p-free factor resulted in a well-fitting model using traditional absolute fit statistics, RMSEA = 0.04 (90% CI = [0.03, 0.04]), CFI = 0.94, TLI = 0.92 (Table S3B). Furthermore, loadings onto p were skewed only slightly relative to the bifactor (orthogonal p-free) model, indicating that any differences in the underlying meaning of p would likely be negligible. Removing the Thought Disorders p-free factor resulted in an adequately fitting model using traditional absolute fit statistics, RMSEA = 0.04 (90% CI = [0.04, 0.05]), CFI = 0.93, TLI = 0.90 (Table S3B). As would be expected, and as indicated by the factor loadings, p was more heavily weighted toward thought disorders symptomatology than in the original bifactor (orthogonal p-free) model.

##### Summary

Of the various alternative bifactor specifications, the bifactor (–p-free Internalizing) model fitted best. Nonetheless, traditional model-fit statistics are biased toward accepting an overfitted bifactor model (Bonifay & Cai, 2017; Bonifay, Lane & Reise, 2017; Reise et al., 2016), so we now consider ancillary fit statistics for the five bifactor models (i.e., orthogonal p-free, oblique p-free, –p-free Externalizing, –p-free Internalizing, and –p-free Thought Disorders).

#### Comparing ancillary statistics for different specifications of bifactor models of psychopathology

Researchers using bifactor models are encouraged to assess ancillary statistics to inform “(a) the quality of unit-weighted total and subscale score composites, as well as factor score estimates, and (b) the specification and quality of a measurement model in structural equation modeling” (Rodriguez et al., 2016, pp. 137). Table 1 (and Table S4 in the Supplemental Material) compares the five bifactor models (i.e., orthogonal p-free, oblique p-free, –p-free Externalizing, –p-free Internalizing, and –p-free Thought Disorder) using the following factor-level statistics: omega (ω, ω_s_), omega H (ω_H_, ω_HS_), relative omega, the H Index, and the explained common variance (ECV). Importantly, these statistics were designed to evaluate the adequacy of scores when created outside the structural equation modeling framework (i.e., when creating unit-weighted total and subscale composites or when factors scores are saved and treated as observed variables in subsequent analyses), which is how they are most commonly used in practice.

**Table 1.** Ancillary Factor-Level Fit Statistics for the Bifactor Models of Psychopathology

Factor and model	ω/ωS	ωH/ωHS	Relative ω	H Index	Explained common variance
p-factor					
Bifactor (orthogonal p-free)	0.82	0.65	0.79	0.77	0.55
Bifactor (oblique p-free)	0.81	0.62	0.76	0.76	0.48
Bifactor (–p-free Externalizing)	0.81	0.68	0.84	0.77	0.61
Bifactor (–p-free Internalizing)	0.81	0.64	0.79	0.79	0.59
Bifactor (–p-free Thought Disorder)	0.81	0.63	0.78	0.79	0.58
Externalizing p-free					
Bifactor (orthogonal p-free)	0.70	0.44	0.63	0.61	0.24
Bifactor (oblique p-free)	0.74	0.13	0.17	0.54	0.14
Bifactor (–p-free Internalizing)	0.70	0.47	0.67	0.63	0.26
Bifactor (–p-free Thought Disorder)	0.69	0.49	0.70	0.63	0.27
Internalizing p-free					
Bifactor (orthogonal p-free)	0.68	0.11	0.16	0.37	0.09
Bifactor (oblique p-free)	0.66	0.41	0.62	0.53	0.18
Bifactor (–p-free Externalizing)	0.66	0.38	0.57	0.53	0.20
Bifactor (–p-free Thought Disorder)	0.67	0.27	0.40	0.48	0.15
Thought Disorders p-free					
Bifactor (orthogonal p-free)	0.66	0.30	0.46	0.41	0.12
Bifactor (oblique p-free)	0.66	0.52	0.79	0.61	0.21
Bifactor (–p-free Externalizing)	0.66	0.44	0.67	0.54	0.20
Bifactor (–p-free Internalizing)	0.66	0.35	0.53	0.45	0.15

Note: Ancillary fit statistics for the one-factor, higher-order factor, and correlated-factors models are shown in Table S4 in the Supplemental Material.

##### Omega

Omega (ω, ω_s_; McDonald, 1999) provides a model-based estimate of the reliability of the factor scores. It represents the proportion of variance in the observed general (ω) and p-free (ω_s_) scores that is attributable to all modeled sources of common variance. Interpretation of ω follows that of coefficient α (Cronbach, 1951; Rodriguez et al., 2016), with scores ≥ 0.70 considered acceptable and scores ≥ 0.80 considered good. Across all bifactor models, ω for p ranged from 81% to 82%, suggesting that p has good reliability regardless of the model used. Reliability of the p-free factors was questionable: p-free Externalizing ω_s_ ranged from 69% to 74%, p-free Internalizing ω_s_ ranged from 66% to 68%, and p-free Thought Disorder ω_s_ were all 66%.

##### Omega H/omega HS

Omega H (ω_H_) estimates the degree to which a unit-weighted total score reflects individual differences in p and is defined as the proportion of total score variance that can be attributed to the general factor after accounting for all p-free factors. Similarly, Omega HS (ω_HS_) for the p-free factors represents the degree to which a unit-weighted subscale score reflects the intended p-free factor and is defined as the proportion of subscale score variance that can be attributed to the p-free factor after accounting for the general factor. Generally, scores greater than 0.50 can be considered acceptable, whereas scores greater than 0.75 are preferred (Reise et al., 2010). Across all bifactor models, ω_H_ for p ranged from 0.62 to 0.68, suggesting that a total score of symptoms predominantly reflects a single general factor regardless of the model used. ω_HS_ ranged from 0.13 to 0.49 for the Externalizing p-free factor, from 0.11 to 0.41 for the Internalizing p-free factor, and from 0.30 to 0.52 for the Thought Disorders p-free factor, suggesting that in most models, unit-weighted subscale scores would reflect p more so than the intended p-free factor.

##### Relative omega

Relative ω provides a comparison of ω and ω_H_. For p, it represents the percentage of reliable variance in the multidimensional composite that is due to the general factor; for p-free factors, relative ω represents the proportion of reliable variance in the subscale composite that is independent of the general factor. To the best of our knowledge, no published empirically derived guidelines exist for relative ω, but higher values indicate more reliable variance. Consistent with results from ω and ω_H_, relative ω for p ranged from 76% to 84%, indicating that the majority of the reliable variance in a unit-weighted total score of p is due to the general factor, regardless of the model used. Relative ωs ranged from 17% to 70% for the p-free Externalizing factors, from 16% to 62% for the p-free Internalizing factors, and from 46% to 79% for the p-free Thought Disorders factors. This suggests that there was more variability and generally less reliable variance in the p-free factors.

##### H Index

The H Index captures the proportion of variance explained by a latent factor divided by variance unexplained by that latent factor and “reflects the extent to which a latent variable is represented by its indicators and thus how likely it is to be replicated across studies” (Watts et al., 2019, p. 1288). H values greater than 0.70 to 0.80 suggest a well-defined latent variable (Hancock & Mueller, 2001) that is likely to replicate across studies (Rodriguez et al., 2016). The H Index for p across the bifactor models ranged from 0.76 to 0.79, indicating that, regardless of the model used, the p factor is well-defined by its indicators. In general, the H Index for the p-free Externalizing, Internalizing, and Thought Disorders factors remained low (< ~0.60), suggesting that these were not well represented by their indicators and, thus, unlikely to replicate across studies.

##### Explained common variance

ECV represents the proportion of all common variance explained by a factor and ranges from 0 to 1; values ≥ 85% for the general factor indicate sufficient unidimensionality to warrant a one-factor model (Stucky & Edelen, 2015; Stucky et al., 2013). In our bifactor models, ECVs for the general factor and the p-free factors totaled to 100%. Across all models, 48% to 61% of the extracted variance was explained by p, 14% to 26% was explained by the p-free Externalizing factor, 9% to 20% was explained by the p-free Internalizing factor, and 12% to 21% was explained by the p-free Thought Disorders factor. In all models, ECVs for p were considerably below the values indicative of unidimensionality. This suggests that scores created from the symptom scales are likely multidimensional, and when scores are created for p, this multidimensionality should be accounted for by including p-free factors.

##### Summary

Ancillary fit statistics indicate that whereas p appears reliably specified regardless of the model used, the p-free factors are much more variable. This suggests that p should replicate across studies but that the p-free Externalizing, Internalizing, and Thought Disorders factors may not. That said, the ancillary statistics also suggest that symptoms of mental disorders assessed here are not fully unidimensional. In particular, Externalizing behaviors that are independent of p may be of particular interest and worthy of interrogation, at least in the age group studied here (who were all 18 years old).

### Comparing psychopathology factor scores across models

#### Factor extraction

Because it is common practice to extract factor scores from structural equation models to use them in further analyses, we examined the *factor determinacy* of the respective p and p-free factor scores. Higher factor determinacy indicates a higher correlation between factor scores and the factor. All in all, factor determinacy scores were acceptable in all models examined. Factor determinacy for p ranged from 0.83 to 0.89 across all the models (Tables S3A and S3B), factor determinacy scores for the three first-order factors in the correlated-factors and higher-order factor models ranged from 0.86 to 0.87, and factor determinacy scores for the p-free factors in the various bifactor models were lower and ranged from 0.65 to 0.80.

To summarize, factor determinacy scores for the three correlated dimensions of psychopathology and for p were acceptable regardless of the model used. Factor determinacy for the p-free factors in the various bifactor models was more variable.

#### Correlations among factors

We extracted factor scores from each of our models (one-factor model, correlated-factors model, higher-order factor model, and the five bifactor models), saved them, and calculated their correlations with each other (Table 2; see also Table S5 in the Supplemental Material). The p factor scores extracted from the one-factor, higher-order factor, bifactor (orthogonal p-free), and bifactor (–p-free Internalizing) models were all highly correlated (*r*s ≥ .95; Table 2). p extracted from the bifactor (–p-free Thought Disorder) model also correlated highly with p from these models, albeit a bit less (*r*s = .88–.95; Table 2). Recall that p factors from the bifactor (oblique p-free) and bifactor (–p-free Externalizing) models were more heavily weighted to Externalizing. This is evident in correlations between p from these models and p from all other models. Specifically, p factor scores from these models were highly correlated with one another (*r* = .98) but had lower correlations (*r*s = .69–.92) with p extracted from other models (Table 2).

**Table 2.** Correlations Between Extracted Factor Scores and Factor Congruencies of the One-Factor, Higher-Order, the Five Bifactor Models, and Correlated-Factors Model of Early-Adult Psychopathology

Model	1	2	3	4	5	6	7
1. One-factor	—	.99	.99	.95	.96	.98	.98
2. Higher-order	.99	—	.99	.91	.93	.99	.98
3. Bifactor (orthogonal p-free)	.98	.99	—	.92	.93	1.00	.98
4. Bifactor (oblique p-free)	.87	.79	.80	—	.99	.90	.87
5. Bifactor (–p-free Externalizing)	.92	.85	.84	.98	—	.91	.90
6. Bifactor (–p-free Internalizing)	.95	.98	.99	.77	.79	—	.96
7. Bifactor (–p-free Thought Disorder)	.94	.95	.93	.69	.76	.88	—

Note: Values below the diagonal are correlations between extracted factor scores; values above the diagonal are factor congruencies. Factor congruency values greater than .95 are “considered equal”; values between .85 and .94 are considered to have “fair similarity” (Lorenzo-Seva & ten Berge, 2006).

In addition to reporting the correlations among factor scores from different models, we also calculated factor congruencies across the models (Table 2). Factor congruencies indicate the similarity between factors derived in factor analysis (Lorenzo-Seva & ten Berge, 2006). All the congruence coefficients were approximately equal to .90, a value which indicates a high degree of factor similarity.

To summarize, although there were subtle differences in the correlations among the factor scores for p extracted from the various models, all were very highly correlated with one another.

### Genetic and environmental influences on psychopathology

We begin our evaluation of the risk factors of psychopathology by evaluating the genetic and environmental architecture of factors derived from different statistical models of the structure of psychopathology. The classical twin model has been used to quantify the contribution of both genetic and environmental causes of variation in psychological traits and in disease susceptibility. The results of twin studies are often said to serve as signposts for guiding further etiological research. Thus, information about whether different statistical models yield similar or different estimates of genetic effects on p and on p-free factors can be used to inform the search for the shared and unique genetic causes of variation in mental disorders (e.g., Allegrini et al., 2020; Grotzinger et al., 2022). Likewise, information about whether different statistical models yield similar or different estimates of shared, general environmental risks versus specific environmental risks for psychopathology can be used to inform exposomics.

Table S6 in the Supplemental Material shows the correlations between monozygotic twins and dizygotic twins, as well as the results of behavioral-genetic models fitted to these data. In behavioral-genetic model fitting, variation in phenotype (e.g., p) is assumed to be influenced by latent additive genetic (*A*), common environmental (*C*), and nonshared environmental (*E*) factors. Model fitting was conducted using *Mplus* (Version 8.5; Muthén & Muthén, 2017).

Four findings stand out. First, the three dimensions from the correlated-factors model were all under considerable genetic influence: 38% for Thought Disorders psychopathology, 47% for Externalizing psychopathology, and 51% for Internalizing psychopathology. There was a hint that shared environmental influences shaped Externalizing psychopathology (11%), a point estimate that is in line with those provided in quantitative reviews of twin studies of Externalizing phenotypes (Burt, 2009; Polderman et al., 2015). In contrast, there was scant evidence for shared environmental influences on Internalizing and Thought Disorder psychopathology. Second, consistent with heritability estimates for the three psychopathology dimensions from the correlated-factors model, approximately 50% of the variation in p was accounted for by additive genetic factors regardless of the model used to extract p. Third, regardless of the bifactor model used, the p-free Internalizing and Thought Disorders factors were under considerably less genetic influence and were suffused with variation attributable to *E*, which includes unique environmental experiences but also, importantly, measurement error. This is consistent with the ancillary model-fitting statistics, which documented that there was generally less reliable variance in these p-free factors regardless of the model used (Table 1). Fourth, consistent with evidence of its greater reliability, the p-free Externalizing factor from the bifactor models was not as suffused with variation attributable to E. Interestingly, there were hints of shared environmental influences on the p-free Externalizing factor, suggesting that environmental influences create similarity between family members’ externalizing behavior apart from family members’ level of psychopathology, p.

### Dimensions of psychopathology, p, and the nomological network of psychopathology

**Fig. 1.**
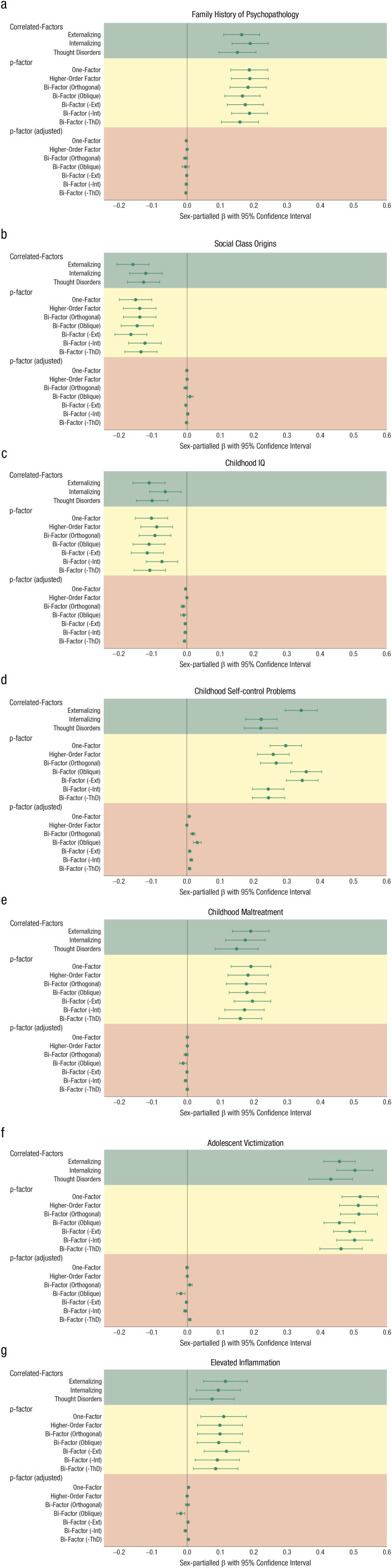
The nomological network of psychopathology. Each panel shows associations between the first-order factors from the correlated-factors model (in green), different versions of the p-factor (in yellow), and adjusted p-factor scores (in brown) with (a) family history of psychopathology, (b) social class origins, (c) childhood IQ, (d) childhood self-control problems, (e) childhood maltreatment, (f) adolescent victimization, and (g) elevated inflammation. Values shown are βs (error bars indicate 95% confidence intervals). Ext = Externalizing; Int = Internalizing; ThD = Thought Disorders.

We evaluated the nomological network of psychopathology by measuring putative causes of mental disorders: family history of mental illness, socioeconomic deprivation, early-emerging cognitive and self-regulation difficulties, exposure to adverse experiences, and inflammation. We selected these risk factors because, historically, some of them have been considered general risk factors (e.g., socioeconomic deprivation), whereas others have been considered specific risk factors (e.g., poor self-control and externalizing disorders; inflammation and depression). It is imperative to evaluate the same risk factors in relation to factors from different statistical models of the structure of psychopathology in the same sample to ensure that any differences in the correlates of psychopathology are not the result of sampling differences. Such a comprehensive analysis has not, to the best of our knowledge, been reported in relation to the multiple, competing statistical models of the structure psychopathology evaluated here. Figure 1 graphs the standardized coefficients obtained from regression models in which psychopathology was the dependent variable; all models controlled for sex, and the standard errors were adjusted for clustering of twins within families.

Three findings stand out. First, the nomological network of the three psychopathology dimensions (Externalizing, Internalizing, and Thought Disorders) from the correlated-factors model is remarkably similar (Fig. 1, green subpanels; see also Table S7 in the Supplemental Material). It may not be surprising that family history of mental illness was equally strongly linked to all three psychopathology dimensions or that growing up in socioeconomically deprived circumstances was equally strongly linked to all three psychopathology dimensions. But there was also little evidence of specificity between children’s early-emerging psychological characteristics and the three psychopathology dimensions. Low childhood IQ was equally strongly linked to all three psychopathology dimensions. Poor childhood self-control was also linked to all three psychopathology dimensions, although the association with Externalizing psychopathology was significantly stronger. Similarly, there was little evidence of specificity between exposure to adverse events and the three psychopathology dimensions. Childhood maltreatment and adolescent victimization experiences were both equally strongly linked to all three psychopathology dimensions. Finally, elevated levels of chronic inflammation were equally apparent across all three psychopathology dimensions.

The second finding that stands out is that regardless of the model from which p was extracted, the nomological network of p was the same; that is, the meaning of p did not depend on whether it was derived from the one-factor, higher-order factor, or bifactor models (Fig. 1, yellow subpanels).

The third finding is that the lack of specificity observed in the correlated-factors model is recapitulated in p. That is, we observed that the risk factors for psychopathology were similar across Externalizing, Internalizing, and Thought Disorders, and this was parsimoniously reflected in p, a latent manifestation of shared causal factors that are associated with every mental disorder. This finding bears on the claim that p should “improve on the external validity of the correlated factors model” (Watts et al., 2019, p. 1288). It is not apparent to us why this hypothesis was put forward, or why that should be the case, to the extent that p reflects what is common among diverse, correlated forms of psychopathology. And, indeed, it is not the case. When we modified our regression models by adding the Externalizing, Internalizing, and Thought Disorders dimensions to the list of independent variables, all of the associations between p and the variables in the nomological network were essentially reduced to zero (βs = −0.01 to 0.02), suggesting that p parsimoniously captured the combined information in all forms of psychopathology that we measured (Fig. 1, orange subpanels).

### Exploring the meaning of p-free factors extracted from bifactor models

**Fig. 2.**
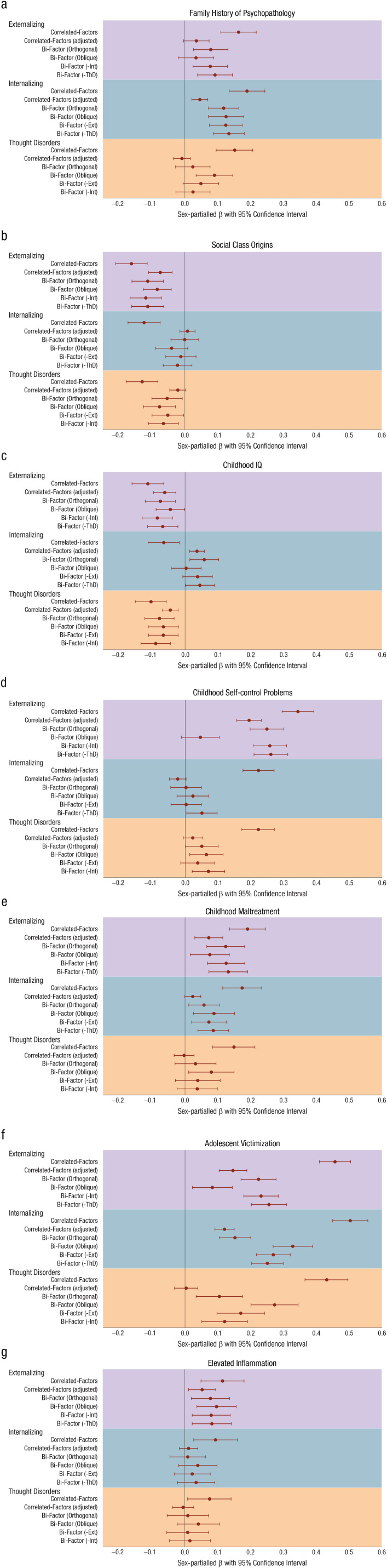
What do the p-free factors from bifactor models mean? The figure shows associations between the three first-order factors from the correlated-factors model, each of the three first-order factors from the correlated-factors model adjusted for the other two first-order factors, and p-free factors from the bifactor models with (a) family history of psychopathology, (b) social class origins, (c) childhood IQ, (d) childhood self-control problems, (e) childhood maltreatment, (f) adolescent victimization, and (g) elevated inflammation. Values shown are βs (error bars indicate 95% confidence intervals). Ext = Externalizing; Int = Internalizing; ThD = Thought Disorders.

To answer the question “What do the p-free factors extracted from bifactor models mean?” we compared the nomological network of psychopathology using (a) the Externalizing, Internalizing, and Thought Disorders dimensions from the correlated-factors model; (b) the Externalizing, Internalizing, and Thought Disorders dimensions from the correlated-factors model after controlling for the other two dimensions (which represent the unique effects of each dimension after taking comorbidity into account); and (c) the p-free Externalizing, Internalizing, and Thought Disorders factors from the bifactor models. Figures 2a to 2g (see also Table S8 in the Supplemental Material) graph the standardized regression coefficients obtained from regression models in which psychopathology was the dependent variable; as before, all models controlled for sex, and the standard errors were adjusted for clustering of twins within families.

Three findings stand out. First, the three unadjusted dimensions of psychopathology (Externalizing, Internalizing, and Thought Disorders) had the strongest absolute associations with variables in the nomological network. Second, adjusting one dimension of psychopathology for the other two dimensions yielded much smaller associations with variables in the nomological network; these were often nonsignificant and even approached zero, suggesting that relatively few of the putative causes we measured were uniquely associated with any psychopathology dimension. Third, associations with p-free factors extracted from the bifactor models were similar to those seen with the adjusted psychopathology dimensions, suggesting that the p-free factors extracted from the bifactor models represent the effect of Externalizing, Internalizing, or Thought Disorders devoid of overlap with the other dimensions. (The exceptions to this pattern involved the association between self-control and the p-free Externalizing factor from the oblique model [Fig. 2d] and between adolescent victimization and some of the p-free factors [Fig. 2f].) Basically, studying the p-free factors from the bifactor models is tantamount to studying “pure” disorders; that is, of studying one mental-disorder diagnosis while controlling for all other mental disorders, which typically yields smaller associations with variables in the nomological network of mental disorders.

### Sensitivity analyses

In our criterion validity tests, we estimated associations with external correlates using factor scores extracted from *Mplus*. When using maximum likelihood estimation with robust standard errors with variables treated as continuous, *Mplus*’s default method of factor score extraction is the maximum a posteriori (aka regression) method. Some readers may wish to see these associations using latent factors in the structural equation modeling framework because there is concern that factor scores might smooth over some of the differences between the different p-factors. Other readers may be concerned that many researchers and clinicians are not familiar with the nuances of latent variable modeling and wish to see these associations using the sum (or average) of relevant symptoms. In Table S9 in the Supplemental Material, we compare associations with external correlates using (a) extracted factors, (b) latent factors, and (c) the sum of the relevant symptoms. For the most part, the results are very similar; for example, the vast majority of associations estimated using factor scores fall within the 95% CIs of associations estimated with latent factors and summed scores.

## Discussion

Our goal in this article was to review the history of the idea about a general factor of psychopathology, address methodological qualms about whether its existence depends on modeling approaches, and refresh research directions.

The positive manifold of psychopathology refers to the fact that different mental disorders tend to correlate with each other. That is, not only are depression and anxiety (Internalizing disorders) highly correlated, not only are conduct disorder and substance dependencies (Externalizing disorders) highly correlated, and not only are schizophrenia and mania (Thought Disorders) highly correlated, but all of these disorders are intercorrelated, pointing to the possibility that a wide range of mental disorders are unified by a general latent dimension of psychopathology, p. To study the general factor of psychopathology, we went into the weeds using data about mental disorders and their correlates in the E-Risk study of behavioral development. This was a necessary excursion because of the misapprehension that p is simply a product of how a general factor is estimated in model-fitting exercises: at best, the result of bifactor models that produce fits that are too good; at worst, the strange offspring of statistical shenanigans. Two main findings emerged.

First, p factors extracted from different statistical models (i.e., one-factor, higher-order factor, or bifactor models) were all very highly correlated, suggesting that they all rank-order individuals in a similar way on a latent dimension of general psychopathology. Second, regardless of the model from which p was extracted, the nomological network of p’s correlates was similar, suggesting that the meaning of the p factor is fairly consistent regardless of how it is modeled. Of course, these results need to be viewed in light of limitations related to sample size; cohort, period, and age effects; and ethnicity. E-Risk is a modestly sized, nationally representative birth cohort; the participants were all born in the mid-1990s and assessed as 18-year-olds, and they are primarily White Europeans. Fortunately, there is increasingly more inclusive research about the structure of psychopathology, suggesting racially/ethnically robust results (He & Li, 2021). In addition, the set of measures and constructs of psychopathology that we modeled is not exhaustive; for example, we did not interview participants about symptoms of bipolar disorder, obsessive-compulsive disorder, and autism, among many impairing conditions. A conservative conclusion from our analysis is that if the sample and content are the same, the resulting p factor will be similar regardless of how it is modeled, but it does not rule out the possibility that there may be somewhat different p factors at different ages, in different historical eras, in different ethnic groups, or if the psychopathology measurement content changes. In this regard, it is helpful to evaluate the present findings in relation to other recent reports. The findings reported here echo the conclusions reached in an analysis of data from preadolescents in the Adolescent Brain Cognitive Development (ABCD) Study (D. A. Clark et al., 2021), youth in the Reproducible Brain Charts (RBC) initiative (Scopel Hoffmann et al., 2022), and adults in the *National Epidemiologic Survey on Alcohol and Related Conditions (NESARC)*: “All latent general factors of psychopathology tended to perform similarly well across tests of reliability and validity” (Forbes et al., 2021, p. 313).

Collectively, we think these findings may help to allay concerns about modeling p. Indeed, p was not borne out of a specific factor analytic approach, as some scholars have suggested (Greene et al., 2022). Rather, our thinking about p was stimulated by empirical observations made over years while repeatedly assessing mental disorders in the representative Dunedin birth cohort. Understanding this history of the idea of p may help to refresh research.

The first observation that led us to think about p grew out of the specificity conundrum. Most research on mental disorders tries to find specific causes of specific disorders. What genetic factors cause schizophrenia? What altered brain morphology causes ADHD? What types of child maltreatment cause depression? To answer such questions, the common strategy, historically, has been to compare cases that have a specific disorder with controls (e.g., children meeting criteria for conduct disorder vs. healthy controls) and, more recently, to study differences between people in their location on higher-order dimensions of psychopathology (e.g., the Externalizing spectrum). But the search for causal specificity has been elusive. Evidence from genetics, brain imaging, psychosocial, and macrosocial research shows that many of the putative causes of mental disorder are transdiagnostic (Caspi & Moffitt, 2018; Sprooten et al., 2022). If, as data document, the major dimensions of psychopathology are highly correlated, and if they share many of the same causes, correlates, and consequences, then maybe psychopathology can be more parsimoniously described by a general factor. In this sense, p parsimoniously recapitulates the nonspecificity so frequently observed at the level of first-order psychopathology dimensions that are derived from the correlated-factors model and, in turn, at the lower-order level of specific disorders that are summarized by these dimensions.

The second observation that led us to think about p emerged from developmental research. When people are followed longitudinally, not only is there continuity of the same disorders over time, but also people who meet diagnostic criteria for a specific disorder at one age are also at significantly higher risk for meeting criteria for every other different disorder at subsequent ages. Over decades, people experience many changing disorders and shift between different internalizing, externalizing, and thought disorders (Caspi et al., 2020; Plana-Ripoll et al., 2019). These longitudinal data suggest that it is not a surprise that different disorders have the same causes, correlates, and consequences because it is the same person who has the different disorders when the person is followed over time. p was invoked to take into account early onset, persistent duration, co-occurrence, and sequential comorbidity of mental disorders across the life course (Caspi et al., 2014).

A few decades ago, a cognate field of research was trying to synthesize similar observations. That field, criminology, showed that virtually everyone breaks the law if followed long enough, but “crime careers” are defined by three developmental parameters: age of onset of offending, life-course duration of offending, and diversity of offense types committed (across groupings such as fraud, theft, and violence; Blumstein et al., 1986a). These three parameters tend to covary within individuals. For example, a “crime career” can be early-onset, chronic, and diverse or late-onset, brief, and specialized, or any pattern in between. Early-onset, long duration, and diversity found together signal more significant liability to a serious crime career (Moffitt, 1993; Piquero et al., 2003). We co-opted the developmental approach from criminology not because we equate mental disorder with crime. Rather, we did so because just as the U.S. National Academy of Sciences report on the criminal-careers approach revolutionized crime research and justice policy (Blumstein et al., 1986b), we thought that evidence about developmental features of mental-disorder histories may likewise have implications for research, practice, and public understanding.

**Fig. 3.**
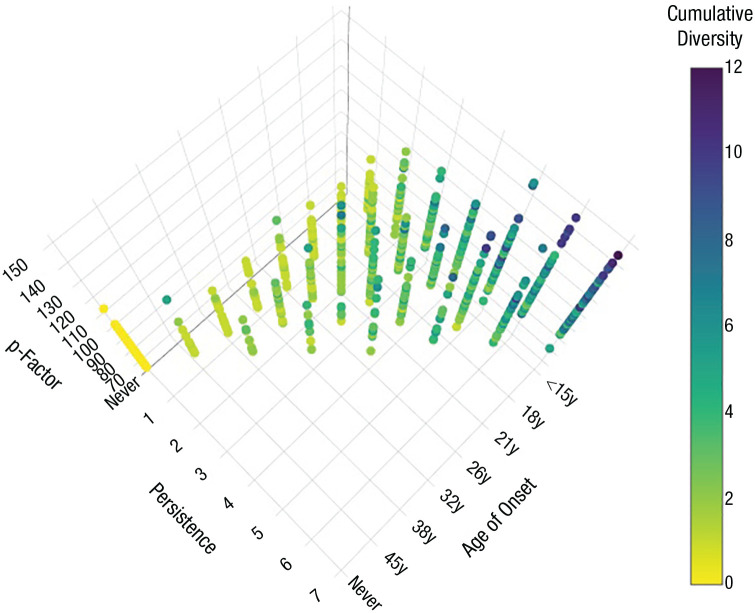
p is a developmental life-course phenomenon. Higher p—derived from a factor analysis of all symptoms reported by participants over the first half of their lives—is associated with early onset, persistence, and cumulative diversity of mental disorders, from age 11 to age 45 (data from Caspi et al., 2020). y = years.

This, then, is the essence of p: Virtually everyone experiences mental disorder if followed long enough, but younger age of onset of disorder, longer life-course duration of disorder, and more diversity of disorders (across groupings of Internalizing, Externalizing, and Thought Disorders) tend to covary within individuals, together signaling a serious mental-disorder life history. Figure 3 illustrates this using data from the Dunedin Study. The three-dimensional model shows that higher p—derived from a factor analysis of all symptoms reported by participants over the first half of their lives—is associated with earlier onset, longer persistence, and greater cumulative diversity of mental disorders, from age 11 to age 45 (data for this figure were obtained from Caspi et al., 2020). Although most researchers do not have such longitudinal mental-disorder data, modeling cross-sectional data about diverse mental disorders can provide an imperfect but reasonable proxy representation of this life-course reality. Cross-sectional data can be a good proxy because greater comorbidity at any given point in time is associated with earlier age of onset, persistence, and prior and subsequent diversity. The bottom line is that p is a surrogate for a developmental life-course phenomenon; it was not the product of theorizing to explain a factor analysis finding.

Ten years after the possibility of a general factor of psychopathology was suggested, it is time to redirect energies away from continued dueling over various statistical models. Here we offer four ideas, from the practical to the ambitious.

First, research about p is best done by studies that measure a wide range of psychopathology content, rather than by studies that focus on a limited set of disorders or dimensional phenotypes. This is the only way to resolve the specificity conundrum and to figure out what is common across diverse disorders. However, many extant studies as well as new initiatives that are intended to serve up discovery data to the research community, including the UK Biobank and All of Us Research Program, unfortunately measure a small fraction of common disorders rather than collect comprehensive data about multiple disorders. Researchers often debate the discovery trade-off between sample size and depth of phenotyping in studying mental disorders (Sanchez-Roige & Palmer, 2020), but breadth of phenotyping is often left out of these discussions despite the fact that it offers a necessary route to investigating what is common and what is unique about different mental disorders. The Philadelphia Neurodevelopment Cohort is a good example of a research initiative that has measured broadly (Satterthwaite et al., 2016). Frameworks such as the Hierarchical Taxonomy of Psychopathology (HiTOP) will enable new research programs to implement broad phenotyping (Conway, Forbes, & South, 2022; Kotov et al., 2017) necessary for generating new insights about the structure of psychopathology.

Second, to the extent that researchers will continue to model general factors of psychopathology with their data, it would be helpful if they reported results *both* for the first-order dimensions of psychopathology derived from the correlated-factors model and for p of their choosing (higher-order, bifactor, or even a one-factor model; the reason for this is, as has now been shown, there is no evidence for the superiority of any one way of modeling p). Because the bifactor model has dominated research about the general factor of psychopathology, many researchers have stopped reporting about the first-order dimensions of psychopathology in their data and now often report only about p and the p-free factors from the bifactor model. This has created mix-ups. The p-free factors from the bifactor model and the first-order factors from the correlated-factors model are often given the same names, leading readers to conflate them and risking confusion in data integration and synthesis. Moreover, it is vital to compare results for p with results for the unpartialed first-order dimensions from the correlated-factors model in order to evaluate the hypothesis that p is a parsimonious representation of many of the causes, correlates, and consequences of psychopathology that have often proven to be nonspecific. We suggest that (a) we are not yet ready to abandon first-order dimensions of psychopathology in favor of p and should continue to evaluate them alongside p and (b) p-free factors are no substitute for interrogating first-order dimensions of psychopathology.

This recommendation raises the vexing problem of the p-free factors in the bifactor representation of the structure of psychopathology. Some research suggests that p-free factors from bifactor models may yield constructs that are better matched to theoretical predictions about causes and consequences that are specific to some mental disorders and not others (e.g., p-free internalizing factors should aid the search for the unique causes and consequences of internalizing symptoms that are not also associated with other mental health problems; Brislin et al., 2022; Moore et al., 2020). But the caveat is that p-free factors, as shown here and elsewhere (e.g., D. A. Clark et al., 2021; Forbes et al., 2021), are not always very reliable and should be accompanied by a caution to “use with care.” A tougher recommendation is that reports of p-free factors should include replication in two or more data sets. We suggest that if a research program is concerned with testing specificity, much the same can be achieved by multivariate analyses of the first-order dimensions of psychopathology from the correlated-factors model as by studying p-free factors from the bifactor model, with less baggage although not without statistical challenges posed by partialing (Lynam et al., 2006).

Some readers may think that we are unduly pessimistic about efforts to identify disorder-specific causes, correlates, and consequences of mental disorders. In fact, it has been suggested that, in the present study, we have stacked the deck by including external correlates that are likely to be transdiagnostic and by excluding external correlates that may be specific to particular psychopathology domains. In this regard, it is important to recognize that, increasingly, yesterday’s specific correlates have turned out to be today’s transdiagnostic features. The reason for this knowledge transformation is because, increasingly, yesterday’s specific correlates are being evaluated in studies—such as the present one—that measure a wide range of psychopathology content rather than just a specific disorder or dimension. Consider self-control, which historically was considered the main factor behind externalizing disorders (Gottredson & Hirschi, 1990) but which now appears to be an early-emerging risk factor for multiple internalizing and thought disorders. Consider inflammation, which has been thought to underlie many cases of depression (Bullmore, 2018) but which it now turns out may promote, or be promoted by, many other disorders, including externalizing and thought disorders. Or consider early life experiences of abuse, which were understood to be potent risk factors in the background of many cases of externalizing and internalizing disorders but which are now known to increase the risk of developing psychosis, an idea that only recently went from fringe to mainstream (Read et al., 2005).

Does this mean that disorder-specific risk factors do not exist? No. We will list three predictions and recommendations. First, it means that specific factors are more difficult to identify than has heretofore been appreciated. Much of the search for specific factors has been guided by disorder-specific theories and tested in case-control designs, with the result that the search has often fallen prey to the “streetlight effect.” When the search, as it should, is broadened beyond testing putative specific correlates in relation to one specific disorder/dimension, it turns out that many putative specific factors are not so specific after all. This suggests that breadth of psychopathology phenotyping is crucial for research that seeks to evaluate what is common versus what is unique about different mental disorders. Second, we predict that when longitudinal data are included, disorder-specific risk factors will prove even more difficult to detect because few people keep only a unique set of symptoms over time (Caspi et al., 2020; Lahey et al., 2005; Plana-Ripoll et al., 2019). An interesting exception might be with regard to substance-use disorders that dominate the externalizing spectrum, given that these disorders uniquely involve drug metabolism and pharmacokinetics. Third, it may be that the search for specificity could be better guided by a hierarchical model of psychopathology rather than by comparing different mental disorders, as is usually done (Conway, Kotov, et al., 2022). Consider HiTOP, which tries to disentangle lower-order versus higher-order features of mental disorders. In this system, for example, social anxiety could be viewed in terms of avoidance of physiological arousal (symptom components), a general tendency to avoid threatening situations (fear subfactor), or a basic predisposition to negative affect (internalizing spectrum). By mapping risk factors onto a hierarchical model, it may be possible to identify risk factors that will affect narrow, more specific symptoms and traits versus those that might affect higher-order dimensions (internalizing). For example, whereas specific symptoms and maladaptive traits may reflect more limited, circumscribed exposures (e.g., parental criticism of a child’s physical appearance), higher-order dimensions may reflect contributions of multifaceted exposures that affect many parts of people’s lives (e.g., child abuse).

Third, both researchers and clinicians need better history-taking tools that will allow them to measure the key developmental parameters of p: age of onset, persistence of duration, and cumulative diversity. In the absence of collecting repeated, longitudinal measurements of mental disorders, obtaining accurate lifetime retrospective reports of mental disorders is a priority for research and practice. And yet it is widely recognized that the manner in which most researchers collect lifetime reports of mental disorders yields unreliable retrospective data (Simon & VonKorff, 1995). In clinical practice, the manner in which clinicians gather such information is idiosyncratic. Methods are being refined. An example is the use of life-history calendars that use visual aids, inquire about streams of events, record event sequences, and contextualize questions about various life events to improve the quality of retrospective reports (Caspi et al., 1996; Freedman et al., 1988). Life-history calendars have been shown to yield more reliable information than standardized questionnaires about various vulnerabilities, including illnesses, crime victimization, and absenteeism (Belli et al., 2001;Morselli et al., 2016; Yoshihama et al., 2005). Importantly, calendars have been shown to improve measurement of lifetime experience with mental disorders (Axinn et al., 2020). A developmental view of the general factor of psychopathology prioritizes valid expert history-taking to enhance accurate measurement for research purposes and to support strategic treatment planning in patients’ lives. There exists an opportunity for collaboration between cognitive scientists, psychopathologists, and clinicians to develop accurate data-collection tools for gathering lifetime retrospective reports of mental disorders.

Fourth, factor analysis will not tell us what p is. The descriptive phase of research about the structure of psychopathology, which has led to p, suggests that most mental disorders share something (or some things) in common. Until 10 years ago, this was not widely known. But factor analysis cannot adjudicate between different causes that may give rise to positive correlations between different mental disorders (Fried et al., 2021). Evidence of common variance also does not imply a single unitary cause but could reflect multiple shared causes (Wright & Woods, 2020). Moreover, despite the assumptions of factor models, it is important to recognize that extracting common variance cannot even differentiate between whether p is a shared cause or a shared consequence of different mental disorders (Smith et al., 2020). The journalist Alex Riley (2021) has written that “The p-factor is the dark matter of psychiatry: an invisible, unifying force that might lie behind a multitude of mental disorders.” If most mental disorders share something in common, we need to figure out what that might be and to measure this directly rather than infer it by statistically modeling common variance. This is the next phase of research about p, moving beyond description.

What would such a measure look like? p is, by definition, transdiagnostic. Emotional dysregulation and negative affect have been put forth as transdiagnostic traits that unify different mental disorders (e.g., Lahey et al., 2017). These are attractive candidates because, among other reasons, they offer the opportunity for cross-species analyses of behavioral and emotional problems. But what distinguishes human dysfunction is thought. The human mind is unique in recalling the past; planning for the future; knowing what others know, see, and believe; and navigating complex relationships. And what is unique about human mental disorders are distortions in thinking, perceiving, and sensing that bring about harmful dysfunction. For this reason, we hypothesize that p represents the disordered form and content of thought that permeates practically every disorder dimension. Examples include not only delusions and hallucinations but also difficulty in dealing with uncertainty; difficulty in making decisions; irrational fears, worries, and rumination; intrusive thoughts and memories; unhelpful schemas; reexperiencing trauma; dissociative states; beliefs that something terrible will happen if a behavior is not performed; body image disturbances; hostile attributions made in response to ambiguous social situations; and attributing failure to internal, stable, and global causes. Direct measures of what is presumed to be at the core of p will allow tests of etiology, a way to study continuity and change, and an opportunity to design and evaluate novel interventions. For example, when do disordered thoughts emerge? How do they give rise to the emotions and behaviors that cause harm and dysfunction at different points in the life course? Do interventions directed at disordered thinking have preventative and ameliorative transdiagnostic effects? Whether disordered thought proves key to unraveling what is common to many mental disorders is one hypothesis among others (e.g., Phillips et al., 2022; Southward et al., 2022), but the time is right to move beyond debates about how to model mental disorder/symptom data to measuring what is common to many mental disorders and to evaluating what accounts for individual differences in mental-disorder life histories that vary in their onset, duration, and comorbidity across the life course.

The idea of p has animated research and thinking about transdiagnostic approaches to mental disorders. It has also been unnecessarily shackled by statistical debates about factor analysis. The way forward will be achieved not by statistics but by better research designs: by studies that more routinely gather broad, representative measurement of mental disorders; by more transparent reporting of findings at different levels of the hierarchy of mental-disorder measurements; by ascertaining key developmental parameters in people’s mental-health histories; and by developing new theory-based measurements of what different mental disorders share in common.

## Supplemental Material

sj-docx-1-cpx-10.1177_21677026221147872 – Supplemental material for The General Factor of Psychopathology (p): Choosing Among Competing Models and Interpreting p Click here for additional data file. Supplemental material, sj-docx-1-cpx-10.1177_21677026221147872 for The General Factor of Psychopathology (p): Choosing Among Competing Models and Interpreting p by Avshalom Caspi, Renate M. Houts, Helen L. Fisher, Andrea Danese and Terrie E. Moffitt in Clinical Psychological Science

sj-pdf-2-cpx-10.1177_21677026221147872 – Supplemental material for The General Factor of Psychopathology (p): Choosing Among Competing Models and Interpreting p Click here for additional data file. Supplemental material, sj-pdf-2-cpx-10.1177_21677026221147872 for The General Factor of Psychopathology (p): Choosing Among Competing Models and Interpreting p by Avshalom Caspi, Renate M. Houts, Helen L. Fisher, Andrea Danese and Terrie E. Moffitt in Clinical Psychological Science

## Transparency

*Action Editor:* Aidan G. C. Wright

*Editor:* Jennifer L. Tackett

*Author Contributions*

**Avshalom Caspi:** Conceptualization; Data curation; Funding acquisition; Investigation; Methodology; Project administration; Resources; Writing – original draft.

**Renate M. Houts:** Conceptualization; Data curation; Formal analysis; Investigation; Methodology; Visualization; Writing – original draft.

**Helen L. Fisher:** Data curation; Investigation; Writing – review & editing.

**Andrea Danese:** Investigation; Writing – review & editing.

**Terrie E. Moffitt:** Conceptualization; Data curation; Funding acquisition; Investigation; Writing – review & editing.
