# Supplementary material for: The General Factor of Psychopathology (p): Choosing Among Competing Models and Interpreting p
Source: Clin Psychol Sci. 2023 May 3;12(1):53–82. doi: 10.1177/21677026221147872 (PMC10794018; doi:10.1177/21677026221147872)
Supplement: sj-docx-1-cpx-10.1177_21677026221147872 – Supplemental material for The General Factor of Psychopathology (p): Choosing Among Competing Models and Interpreting p [file sj-docx-1-cpx-10.1177_21677026221147872.docx]

# The general factor of psychopathology (p): Choosing among competing models and interpreting p Caspi, Houts, Fisher, Danese, & Moffitt

**Supplemental Information**

**Supplemental Table 1**. Assessment of symptoms of mental disorders in E-Risk cohort at age 18 years .... 2

**Supplemental Table 2.** Correlations between mental-disorder symptom scales 6

**Supplemental Table 3.** Comparing models of the structure of psychopathology: Traditional fit statistics, standardized factor loadings and factor correlations 7

**Supplemental Table 4.** Ancillary factor-level fit statistics for the models of psychopathology 9

**Supplemental Table 5.** Correlations and factor congruencies between first-order dimensions of psychopathology, and ‘p’ and p-free factors from different models 10

**Supplemental Table 6.** Genetic and environmental influences on psychopathology 11

**Supplemental Table 7.** Relationships between first-order factors and ‘p’ and the nomological net 12

**Supplemental Table 8.** Relationships between first-order factors and p-free factors and the nomological net 13

**Supplemental Table 9.** Sensitive analyses comparing raw-score scales, first-order factors, ‘p’ and p-free factors and the nomological net using regression vs structural equation modeling 14

**Supplemental Figure 1.** Comparing different models of the structure of psychopathology 18

# MPlus Syntax. MPlus Syntax. MPlus Syntax. MPlus Syntax. MPlus Syntax. MPlus Syntax. MPlus Syntax. MPlus Syntax.

Correlated-Factors Model 20

One-Factor Model 21

Higher-Order Factor Model 22

Bi-Factor (Orthogonal p-free) Model 23

Bi-Factor (Oblique p-free) Model 24

Bi-Factor (-p-free Externalizing) Model 25

Bi-Factor (-p-free Internalizing) Model 26

Bi-Factor (-p-free Thought Disorder) Model 27

1

**Supplemental Table 1.** Assessment of symptoms of mental disorders in the E-Risk cohort at age 18 years. Values in parentheses indicate variable names used in MPlus syntax (Supplemental Information, pp 20-27).

Symptom Scale N Mean SD Range Symptoms

|  | | | | | • | Make careless mistakes | • | Feel fidgety or squirmy; feel very restlss |
| --- | --- | --- | --- | --- | --- | --- | --- | --- |
|  |  |  |  |  | • | Don't listen | • | Don’t enjoy doing quiet activities |
|  |  |  |  |  | • | Leave projects unfinished | • | Too loud or noisy |
|  |  |  |  |  | •  • | Disorganized; difficulty organizing tasks that have many steps  Get borded quickly; can't concentrate / mind wanders; tune out when should focus | •  • | Talk too much  Always on the go, in a hurry, as if driven by a motor |
| ADHD  (adhd18) | 2061 | 5.79 | 4.29 | 0 - 18 | •  • | Lack self-discipline; put off tasks that require lots of effort  Misplace wallet, keys, mobile phone, paperwork. Lose things | •  • | Uncomfortable sitting still; need to get up and move.  Make "snap" decisions (too fast) |
|  | | | | | • | Easily distracted/get sidetracked easily; can't resist temptation | • | Difficulty waiting; impatient |
|  |  |  |  |  |  |  | • | Jump into projects without reading the |
|  |  |  |  |  | • | Forgetful: forget appointments, forget to do errands, forget to return calls |  | instructions; impulsive/act without  thinking about what might happen; interrupt, barge into others' |
|  |  |  |  |  |  |  |  | conversations |

Alcohol Dependence (alc18)

2063 1.12 1.68 0 - 11

- Tolerance • Use despite social, psychological, physical problems
- Withdrawal • Use when hazardous
- Loss of control; used more than intended • Interferes with responsibilities
- Unable to cut down • Continued use despite objections
- Spent great deal of time using • Cravings
- Reduced or gave up activities

| Symptom Scale | N | Mean | SD | Range | Symptoms |  |
| --- | --- | --- | --- | --- | --- | --- |
|  |  |  |  |  | - Tolerance | - Reduced or gave up activities |
| Cannabis Dependence (mar18) |  |  |  |  | - Withdrawal | - Use despite social, psychological, physical problems |
|  | 2066 0.33 1.38 0 - 10 | | | |  |  |
|  |  |  |  |  | - Loss of control; used more than intended | - Use when hazardous |
|  |  |  |  |  | - Unable to cut down | - Interferes with responsibilities |
|  |  |  |  |  | - Spent great deal of time using | - Continued use despite objections |
|  |  |  |  |  | - Number of cigarettes smoked per day (0- 3 pts) | - Hard not to smoke in nonsmoking area |
| Tobacco Dependence (smk18) | 2062 | 0.67 | 1.62 | 0 - 10 | - Length of time from waking to first cigarette (0-3 pts) | - Hate to give up cigarette in morning / after hot drink / after meal / with alcohol   / when craving |
|  |  |  |  |  | - Smoke when sick | - Smoke most in the morning |
|  |  |  |  |  | - Bully/threaten | - Broken into car/property |
|  |  |  |  |  | - Initiates physical fights | - Often lies to avoit obligations |
| Conduct Disorder (cd18) |  |  |  |  | - Used a weapon | - Stolen without confronting victim |
|  | 2053 | 2.12 | 2.28 | 0 - 13 | - Physically cruel | - Stayed out late without permission |
|  |  |  |  |  | - Physically cruel to animals | - Run away from home |
|  |  |  |  |  | - Stolen while confronting victim | - Skip school/college |
|  |  |  |  |  | - Destroyed others' property (no fire) |  |
|  |  |  |  |  | - Restless, keyed up or on edge | - Trouble with falling or staying asleep, or waking up tired |
| Generalized Anxiety  Disorder (gad18) |  |  |  |  | - Easily tired | - Tense, sore or aching muscles |
|  | 2066 | 1.13 | 2.15 | 0 - 7 | - Difficulty concentrating on what you were doing | - Any somatization (e.g., jumpy, sweating, dry mouth, nausea, trouble swallowing) |
|  |  |  |  |  | - More irritable than usual |  |

| Symptom Scale | N | Mean | SD | Range | Symptoms |  |
| --- | --- | --- | --- | --- | --- | --- |
|  |  |  |  |  | - Depressed mood | - Fatigue |
| Major Depression (mde18) |  |  |  |  | - Diminished interest | - Feelings of worthlessness/guilt |
|  | 2063 | 1.81 | 2.97 | 0 - 9 | - Weight or appetite change | - Thought difficulties, lack of focus |
|  |  |  |  |  | - Sleep problems | - Thoughts of death/suicide |
|  |  |  |  |  | - Psychomotor problems |  |
|  |  |  |  |  | - Made self sick by eating until uncomfortably full | - Believe you're fat when others say you're too thin |
| Disordered Eating  (eat18) | 2064 | 0.45 | 0.88 | 0 - 5 | - Lost control over amount eaten | - Food dominated life |
|  |  |  |  |  | - Lose more than one stone (3.6 kg or 14 lbs) in 3 month period |  |
|  |  |  |  |  | - Remembering trauma over & over, or when you didn't want to | - Begin to feel isolated or distant from other people |
|  |  |  |  |  | - Having bad dreams or nightmares about trauma | - Find it more difficult to feel love for other people |
|  |  |  |  |  | - Reexperiencing trauma event | - More irritable or short-tempered |
|  |  |  |  |  | - Being in similar situations to trauma is upsetting/anxiety provoking | - Having more trouble than usual falling asleep or staying asleep because of trauma |
| Post-traumatic stress disorder (PTSD18) | 2064 | 1.37 | 3.32 | 0 - 17 | - Heart pound/sweat/physically ill when reminded of trauma | - Feel there was no point in planning for the future |
|  |  |  |  |  |  | - More trouble than usual keeping your mind on what you were doing |
|  |  |  |  |  | - Avoid talking about trauma |  |
|  |  |  |  |  | - Stay away from certain people/places or activities to avoid being reminded of trauma | - Become more concerned about danger or much more careful about things (e.g., checking to see who and what is around you) |
|  |  |  |  |  | - Struggle to remember important parts of trauma | - Feel jumpy or get easily startled by ordinary noises or movements |
|  |  |  |  |  | - Lose interest in activities that were important or enjoyable |  |

| Symptom Scale | N | Mean | SD | Range | Symptoms |  |
| --- | --- | --- | --- | --- | --- | --- |
|  |  |  |  |  | - Thoughts can be read by another | - Felt under the control of special power |
| Psychosis Symptoms (psy18) | 2063 | 0.04 | 0.31 | 0 - 7 | - Sent messages through radio or TV | - Read thoughts of another person |
|  |  |  |  |  | - Being followed or spied on | - See something others cannot hear |
|  |  |  |  |  | - Heard voiced others cannot hear |  |
| Prodromal Symptoms A (prodA18) | 2062 | 0.27 | 0.77 | 0 - 6 | - More sensitive to lights or sounds | - Food may be poisoned |
|  |  |  |  |  | - Can't trust anyone |  |
| Prodromal Symptoms B (prodB18) | 2062 | 0.25 | 0.76 | 0 - 6 | - People or places I know seem different | - Have special abilities or powers beyond my natural talent |
|  |  |  |  |  | - Thinking is unusual or frightening |  |

*Note.* We assessed the same 11 disorder/symptoms as in Schaefer et al. (2018): alcohol dependence, cannabis dependence, tobacco dependence, conduct disorder, attention-deficit hyperactivity disorder (ADHD), major depression, generalized anxiety disorder, post-traumatic stress disorder, disordered eating, psychosis symptoms, prodromal symptoms (we divided these into 2 parcels to allow proper identification of the confirmatory factor models). All symptoms were coded as 0 = absent, 1 = present except prodromal symptoms which study members endorsed on a 3-point (0, 1, 2) scale. Schaefer et al. (2018) treated symptom scales as ordinal; we treat them as continuous.

**Supplemental Table 2.** Correlations between mental-disorder symptom scales in the E-Risk cohort.

|  | ADHD | Alc | Cann | Tob | CD | GAD | MDE | Eat | PTSD | Psych | Prod A |
| --- | --- | --- | --- | --- | --- | --- | --- | --- | --- | --- | --- |
| Alcohol Dependence | 0.30 |  |  |  |  |  |  |  |  |  |  |
| Cannabis Dependence | 0.22 | 0.19 |  |  |  |  |  |  |  |  |  |
| Tobacco Dependence | 0.22 | 0.19 | 0.38 |  |  |  |  |  |  |  |  |
| Conduct Disorder | 0.43 | 0.37 | 0.40 | 0.32 |  |  |  |  |  |  |  |
| Generalized Anxiety Disorder | 0.26 | 0.13 | 0.08 | 0.08 | 0.14 |  |  |  |  |  |  |
| Major Depressive Episode | 0.34 | 0.26 | 0.17 | 0.19 | 0.24 | 0.46 |  |  |  |  |  |
| Eating Disorder | 0.28 | 0.20 | 0.09 | 0.15 | 0.17 | 0.27 | 0.35 |  |  |  |  |
| Post-Traumatic Stress Disorder | 0.19 | 0.12 | 0.08 | 0.17 | 0.15 | 0.24 | 0.34 | 0.22 |  |  |  |
| Psychosis Symptoms | 0.13 | 0.04 | 0.11 | 0.09 | 0.11 | 0.17 | 0.17 | 0.14 | 0.16 |  |  |
| Prodromal Symptoms A | 0.27 | 0.10 | 0.19 | 0.15 | 0.21 | 0.30 | 0.34 | 0.26 | 0.35 | 0.35 |  |
| Prodromal Symptoms B | 0.27 | 0.17 | 0.22 | 0.12 | 0.25 | 0.30 | 0.34 | 0.21 | 0.27 | 0.29 | 0.50 |

*Note.* The shading indicates strength of association, with darker shading indicating stronger correlations. Higher correlations between some disorders (but not others) support the construction of latent factor scores representing the externalizing, internalizing, and thought disorder spectra, whereas the positive correlations between all symptom scales support the construction of a higher-order factor of general psychopathology (which we label “p”). ADHD = attention-deficit hyperactivity disorder; Alc = alcohol dependence; Cann = cannabis dependence; Tob = tobacco dependence; CD = conduct disorder; GAD = generalized anxiety disorder; MDE = major depressive episode; Eat = eating disorder; PTSD = post-traumatic stress disorder; Psych = psychosis symptoms; Prod A = prodromal symptoms (A).

**Supplemental Table 3.** Comparing models of the structure of psychopathology: Traditional fit statistics, standardized factor loadings and factor correlations (**Panel A**).

| Correlated-Factors | | | | One-Factor | Higher-Order Factor | | |  | Bi-Factor (Orthogonal p-free) | | | |
| --- | --- | --- | --- | --- | --- | --- | --- | --- | --- | --- | --- | --- |
| Model Fit Statistics |  |  |  |  |  |  |  |  |  |  |  |  |
| Chi-Square |  | 232.26 |  | 648.61 |  | 232.26 | |  |  | 172.97 | |  |
| Degrees of freedom |  | 51 |  | 54 |  | 51 |  |  |  | 42 |  |  |
| Akaike Information Criteria | 85787.17 | |  | 86615.04 |  | 85787.17 | |  |  | 85660.95 | |  |
| Bayesian Information Criteria (BIC) | 86006.87 | |  | 86817.85 |  | 86006.87 | |  |  | 85931.35 | |  |
| Sample Adjusted BIC | 85882.97 | |  | 86703.47 |  | 85882.97 | |  |  | 85778.85 | |  |
| RMSEA (90% CI) | 0.04 (0.04 – 0.05) | | | 0.07 (0.07 – 0.08) |  | 0.04 (0.04 – 0.05) | |  |  | 0.04 (0.03 – 0.05) | |  |
| Comparative Fit Index |  | 0.92 |  | 0.75 |  | 0.92 |  |  |  | 0.94 |  |  |
| Tucker-Lewis Index |  | 0.90 |  | 0.69 |  | 0.90 |  |  |  | 0.91 |  |  |
| SRMR |  | 0.04 |  | 0.07 |  | 0.04 |  |  |  | 0.03 |  |  |
| Standardized Factor Loadings | Ext | Int | ThD | p | p | Ext | Int | ThD | p | Ext | Int | ThD |
| ADHD | 0.60 |  |  | 0.55 |  | 0.60 |  |  | 0.50 | 0.32 |  |  |
| Alcohol Dependence | 0.48 |  |  | 0.38 |  | 0.48 |  |  | 0.32 | 0.35 |  |  |
| Cannabis Dependence | 0.51 |  |  | 0.38 |  | 0.51 |  |  | 0.25 | 0.47 |  |  |
| Tobacco Dependence | 0.47 |  |  | 0.36 |  | 0.47 |  |  | 0.27 | 0.38 |  |  |
| Conduct Disorder | 0.72 |  |  | 0.50 |  | 0.72 |  |  | 0.37 | 0.68 |  |  |
| Generalized Anxiety Disorder |  | 0.58 |  | 0.50 |  |  | 0.58 |  | 0.50 | *0.23* | |  |
| Major Depressive Episode |  | 0.74 |  | 0.63 |  |  | 0.74 |  | 0.65 | *0.58* | |  |
| Eating Disorder |  | 0.48 |  | 0.45 |  |  | 0.48 |  | 0.47 | *0.08* | |  |
| Post-Traumatic Stress Disorder |  | 0.48 |  | 0.45 |  |  | 0.48 |  | 0.49 | *0.04* | |  |
| Psychosis Symptoms |  |  | 0.43 | 0.34 |  |  |  | 0.43 | 0.28 |  |  | 0.37 |
| Prodromal Symptoms A |  |  | 0.74 | 0.59 |  |  |  | 0.74 | 0.56 |  |  | 0.51 |
| Prodromal Symptoms B |  |  | 0.69 | 0.58 |  |  |  | 0.69 | 0.54 |  |  | 0.39 |
| Externalizing |  |  |  |  | 0.61 |  |  |  |  |  |  |  |
| Internalizing |  |  |  |  | 0.88 |  |  |  |  |  |  |  |
| Thought Disorder |  |  |  |  | 0.79 |  |  |  |  |  |  |  |
| Factor Determinacy | 0.86 | 0.87 | 0.86 | 0.89 | 0.83 | 0.86 | 0.87 | 0.86 | 0.85 | 0.78 | 0.67 | 0.65 |
| Factor Correlations |  |  |  |  |  |  |  |  |  |  |  |  |
| Externalizing |  |  |  |  |  |  |  |  | 0.00 |  |  |  |
| Internalizing | 0.54 |  |  |  |  |  |  |  | 0.00 | 0.00 |  |  |
| Thought Disorder | 0.48 | 0.69 |  |  |  |  |  |  | 0.00 | 0.00 | 0.00 |  |

**Supplemental Table 3, Continued.** Comparing models of the structure of psychopathology: Traditional fit statistics, standardized factor loadings and factor correlations (**Panel B**).

|  | Bi-Factor (Oblique p-free) | | | | Bi-Factor (–p-free Ext) | | | Bi-Factor (–p-free Int) | | | Bi-Factor (–p-free ThD) | | |
| --- | --- | --- | --- | --- | --- | --- | --- | --- | --- | --- | --- | --- | --- |
| Model Fit Statistics  Chi-Square | 111.90 | | | | 345.69 | | | 181.26 | | | 208.09 | | |
| Degrees of freedom | 39 | | | | 47 | | | 46 | | | 45 | | |
| Akaike Information Criteria | 85574.16 | | | | 85996.31 | | | 85688.61 | | | 85750.68 | | |
| Bayesian Information Criteria (BIC) | 85861.46 | | | | 86238.54 | | | 85936.48 | | | 86004.18 | | |
| Sample Adjusted BIC | 85699.43 | | | | 86101.93 | | | 85796.68 | | | 85861.21 | | |
| RMSEA (90% CI) | 0.03 (0.02 – 0.04) | | | | 0.06 (0.05 – 0.06) | | | 0.04 (0.03 – 0.04) | | | 0.04 (0.04 – 0.05) | | |
| Comparative Fit Index | 0.97 | | | | 0.87 | | | 0.94 | | | 0.93 | | |
| Tucker-Lewis Index | 0.95 | | | | 0.82 | | | 0.92 | | | 0.90 | | |
| SRMR | 0.03 | | | | 0.06 | | | 0.04 | | | 0.04 | | |
| Standardized Factor Loadings | p | Ext | Int | ThD | p | Int | ThD | p | Ext | ThD | p | Ext | Int |
| ADHD | 0.69 | *-0.08* |  |  | 0.61 |  |  | 0.48 | 0.35 |  | 0.43 | 0.38 |  |
| Alcohol Dependence | 0.50 | *0.01* |  |  | 0.47 |  |  | 0.31 | 0.36 |  | 0.24 | 0.40 |  |
| Cannabis Dependence | 0.39 | 0.70 |  |  | 0.49 |  |  | 0.23 | 0.48 |  | 0.27 | 0.45 |  |
| Tobacco Dependence | 0.38 | 0.32 |  |  | 0.45 |  |  | 0.25 | 0.39 |  | 0.23 | 0.40 |  |
| Conduct Disorder | 0.64 | *0.22* |  |  | 0.66 |  |  | 0.34 | 0.69 |  | 0.34 | 0.69 |  |
| Generalized Anxiety Disorder | 0.31 |  | 0.53 |  | 0.32 | 0.50 |  | 0.58 |  |  | 0.43 |  | 0.38 |
| Major Depressive Episode | 0.47 |  | 0.55 |  | 0.49 | 0.61 |  | 0.72 |  |  | 0.51 |  | 0.63 |
| Eating Disorder | 0.36 |  | 0.32 |  | 0.36 | 0.30 |  | 0.49 |  |  | 0.37 |  | 0.26 |
| Post-Traumatic Stress Disorder | 0.28 |  | 0.40 |  | 0.32 | 0.30 |  | 0.48 |  |  | 0.44 |  | 0.18 |
| Psychosis Symptoms | 0.16 |  |  | 0.42 | 0.21 |  | 0.40 | 0.26 |  | 0.38 | 0.42 |  |  |
| Prodromal Symptoms A | 0.33 |  |  | 0.69 | 0.42 |  | 0.63 | 0.53 |  | 0.55 | 0.72 |  |  |
| Prodromal Symptoms B | 0.37 |  |  | 0.55 | 0.44 |  | 0.49 | 0.50 |  | 0.43 | 0.67 |  |  |
| Factor Determinacy | 0.85 | 0.76 | 0.75 | 0.79 | 0.87 | 0.74 | 0.75 | 0.87 | 0.79 | 0.69 | 0.87 | 0.80 | 0.73 |
| Factor Correlations |  |  |  |  |  |  |  |  |  |  |  |  |  |
| Externalizing | 0.00 |  |  |  |  |  |  | 0.00 |  |  | 0.00 |  |  |
| Internalizing | 0.00 | *-0.10* |  |  | 0.00 |  |  |  |  |  | 0.00 | 0.00 |  |
| Thought Disorder | 0.00 | *0.13* | 0.58 |  | 0.00 | 0.00 |  | 0.00 | 0.00 |  |  |  |  |

*Note*. RMSEA = root mean square error of approximation; CI = Confidence Interval; SRMR = standardized root mean square residual; ADHD = attention deficit hyperactivity disorder; Ext = Externalizing; Int = Internalizing; ThD = Thought Disorder. *Italics* = p > 0.05.

**Supplemental Table 4.** Ancillary factor-level fit statistics for the models of psychopathology.

| p-factor | | | | | |  |  |  |  |  |  |  |  |  |  | p-free Factors | |  |  |  |  |  |  |  |  |
| --- | --- | --- | --- | --- | --- | --- | --- | --- | --- | --- | --- | --- | --- | --- | --- | --- | --- | --- | --- | --- | --- | --- | --- | --- | --- |
|  |  |  |  |  |  |  |  |  |  | Externalizing | | |  |  |  | Internalizing | |  |  |  | Thought Disorders | | | |  |
|  |  |  |  |  |  |  |  |  |  |  |  |  |  |  |  |  |  |  |  |  |  |  |  |  |  |
|  | OF | HO | OR | OB | -Ext | -Int | -ThD | CF | HO | OR | OB | -Int | -ThD | CF | HO | OR | OB | -Ext | -ThD | CF | HO | OR | OB | -Ext | -Int |
| ω / ωS | .78 | .85 | .82 | .81 | .81 | .81 | .81 | .69 | .76 | .70 | .74 | .70 | .69 | .66 | .78 | .68 | .66 | .66 | .67 | .66 | .76 | .66 | .66 | .66 | .66 |
| ωH / ωHS | -- | .52 | .65 | .62 | .68 | .64 | .63 | -- | .55 | .44 | .13 | .47 | .49 | -- | .44 | .11 | .41 | .38 | .27 | -- | .47 | .30 | .52 | .44 | .35 |
| Relative ω | -- | .62 | .79 | .76 | .84 | .79 | .78 | -- | .73 | .63 | .17 | .67 | .70 | -- | .57 | .16 | .62 | .57 | .40 | -- | .62 | .46 | .79 | .67 | .53 |
| H Index | .80 | .76 | .77 | .76 | .77 | .79 | .79 | .72 | .72 | .61 | .54 | .63 | .63 | .70 | .70 | .37 | .53 | .53 | .48 | .70 | .70 | .41 | .61 | .54 | .45 |
| ECV | 1.0 | .37 | .55 | .48 | .61 | .59 | .58 | .38 | .24 | .24 | .14 | .26 | .27 | .33 | .21 | .09 | .18 | .20 | .15 | .29 | .19 | .12 | .21 | .20 | .15 |

*Note*. OF = One-Factor; HO = Higher-Order; CF = Correlated-Factors; OR = Bi-Factor (Orthogonal p-free); OB = Bi-Factor (Oblique p-free); -Ext = Bi- Factor (-p-free Externalizing); -Int = Bi-Factor (-p-free Internalizing); -ThD = Bi-Factor (-p-free Thought Disorders). ωH / ωHS and Relative ω could not be calculated for the One-Factor Model because it had no p-free factors and the Correlated-Factors Model because it did not estimate p.

ECV = Explained Common Variance.

**Supplemental Table 5.** Correlations and factor congruencies between first-order dimensions of psychopathology, and ‘p’ and p-free factors from different models.

| Correlated-Factors | | | |  |  | General Factor | | |  |  |  |  |  |  |  |  | p-free Factors | |  |  |  |  |  |  |  |
| --- | --- | --- | --- | --- | --- | --- | --- | --- | --- | --- | --- | --- | --- | --- | --- | --- | --- | --- | --- | --- | --- | --- | --- | --- | --- |
|  |  |  |  |  |  |  | p-factor | |  |  |  |  | Externalizing | |  |  |  | Internalizing | |  |  | Thought Disorders | | |  |
|  |  |  |  |  |  |  |  |  |  |  |  |  |  |  |  |  |  |  |  |  |  |  |  |  |  |
|  | Ext | Int | ThD | OF | HO | OR | OB | -Ext | -Int | -ThD | HO | OR | OB | -Int | -ThD | HO | OR | OB | -Ext | -ThD | HO | OR | OB | -Ext | -Int |
| Correlated-Factors | | | | | | | | | | | | | | | | | | | | | | | | | |
| Ext | -- | -- | -- | .58 | .50 | .49 | .79 | .77 | .46 | .44 | 1.0 | .98 | .62 | .98 | .99 | -- | -- | -- | -- | -- | -- | -- | -- | -- | -- |
| Int | .67 | -- | -- | .61 | .66 | .68 | .48 | .48 | .73 | .57 | -- | -- | -- | -- | -- | 1.0 | .84 | .99 | .99 | .96 | -- | -- | -- | -- | -- |
| ThD | .61 | .83 | -- | .54 | .56 | .53 | .35 | .41 | .49 | .69 | -- | -- | -- | -- | -- | -- | -- | -- | -- | -- | 1.0 | .99 | 1.0 | 1.0 | .99 |
| General Factor ‘p’ | | | | | | | | | | | | | | | | | | | | | | | | | |
| OF | .84 | .94 | .90 | -- | .99 | .99 | .95 | .96 | .98 | .98 | .58 | .56 | .33 | .56 | .57 | .61 | .50 | .60 | .60 | .58 | .54 | .53 | .53 | .53 | .53 |
| HO | .75 | .97 | .92 | .99 | -- | .99 | .91 | .93 | .99 | .98 | -- | .49 | .31 | .49 | .50 | -- | .56 | .66 | .65 | .64 | -- | .56 | .56 | .56 | .56 |
| OR | .74 | .98 | .88 | .98 | .99 | -- | .92 | .93 | 1.00 | .98 | .49 | -- | .24 | .47 | .47 | .68 | -- | .68 | .67 | .65 | .53 | -- | .53 | .52 | .52 |
| OB | .97 | .73 | .62 | .87 | .79 | .80 | -- | .99 | .90 | .87 | .79 | .76 | -- | .76 | .77 | .48 | .41 | -- | .47 | .46 | .35 | .34 | -- | .34 | .34 |
| -Ext | .99 | .77 | .71 | .92 | .85 | .84 | .98 | -- | .91 | .90 | .77 | .75 | .46 | .76 | .76 | .48 | .40 | .47 | -- | .46 | .41 | .40 | .41 | -- | .40 |
| -Int | .69 | .99 | .83 | .95 | .98 | .99 | .77 | .79 | -- | .96 | .46 | .43 | .22 | -- | .44 | .73 | .61 | .73 | .72 | .70 | .49 | .48 | .49 | .49 | -- |
| -ThD | .66 | .87 | .99 | .94 | .95 | .93 | .69 | .76 | .88 | -- | .44 | .42 | .25 | .42 | -- | .57 | .45 | .57 | .56 | -- | .69 | .68 | .69 | .69 | .68 |
| p-free Factors |  |  |  |  |  |  |  |  |  |  |  |  |  |  |  |  |  |  |  |  |  |  |  |  |  |
| Externalizing |  |  |  |  |  |  |  |  |  |  |  |  |  |  |  |  |  |  |  |  |  |  |  |  |  |
| OR | .80 | .12 | .11 | .36 | .23 | .19 | .68 | .69 | .14 | .15 | .80 | -- | .70 | 1.0 | 1.0 | -- | -- | -- | -- | -- | -- | -- | -- | -- | -- |
| OB | .37 | .02 | .13 | .17 | .11 | .03 | .17 | .32 | -.01 | .11 | .37 | .53 | -- | .70 | .66 | -- | -- | -- | -- | -- | -- | -- | -- | -- | -- |
| -Int | .81 | .13 | .15 | .38 | .25 | .21 | .69 | .70 | .14 | .18 | .81 | 1.0 | .53 | -- | 1.0 | -- | -- | -- | -- | -- | -- | -- | -- | -- | -- |
| -ThD | .83 | .20 | .09 | .40 | .28 | .25 | .74 | .72 | .21 | .14 | .83 | .98 | .45 | .97 | -- | -- | -- | -- | -- | -- | -- | -- | -- | -- | -- |
| Internalizing |  |  |  |  |  |  |  |  |  |  |  |  |  |  |  |  |  |  |  |  |  |  |  |  |  |
| OR | .10 | .52 | .13 | .28 | .37 | .36 | .17 | .16 | .48 | .14 | .10 | -.16 | -.13 | .20 | -.05 | .52 | -- | .83 | .89 | .95 | -- | -- | -- | -- | -- |
| OB | .12 | .81 | .66 | .60 | .72 | .72 | .20 | .26 | .78 | .67 | .12 | -.44 | -.22 | .44 | -.37 | .81 | .56 | -- | .99 | .95 | -- | -- | -- | -- | -- |
| -Ext | .08 | .75 | .40 | .48 | .59 | .61 | .19 | .19 | .72 | .43 | .08 | -.41 | -.28 | .44 | -.29 | .75 | .80 | .92 | -- | .99 | -- | -- | -- | -- | -- |
| -ThD | .23 | .66 | .17 | .41 | .49 | .51 | .32 | .29 | .63 | .22 | .23 | -.10 | -.18 | .15 | .05 | .66 | .93 | .65 | .88 | -- | -- | -- | -- | -- | -- |
| Thought Disorders | | | | | | | | | | | | | | | | | | | | | | | | | |
| OR | .10 | .20 | .70 | .34 | .36 | .30 | .04 | .17 | .21 | .62 | .10 | -.11 | .16 | .06 | -.24 | .20 | -.31 | .26 | -.10 | -.44 | .70 | -- | 1.0 | 1.0 | 1.0 |
| OB | .19 | .60 | .88 | .61 | .68 | .63 | .18 | .31 | .59 | .83 | .19 | -.25 | .09 | .22 | -.32 | .60 | .06 | .70 | .39 | .01 | .88 | .86 | -- | 1.0 | 1.0 |
| -Ext | .06 | .40 | .79 | .45 | .51 | .46 | .05 | .17 | .40 | .73 | .06 | -.32 | .00 | .27 | -.41 | .40 | -.11 | .55 | .21 | -.20 | .79 | .94 | .96 | -- | 1.0 |
| -Int | .14 | .20 | .71 | .36 | .38 | .31 | .09 | .21 | .21 | .64 | .14 | -.06 | .18 | .01 | -.20 | .20 | -.36 | .23 | -.15 | -.48 | .71 | 1.0 | .84 | .93 | -- |

-

-

-

-

-

-

-

-

Note. Below diagonal = Correlations between extracted factor scores; above diagonal = factor congruencies. Factor congruency values > .95 = “considered equal”; values between 0.85-0.94 = “fair similarity” (Lorenzo-Seva & ten Berg, 2006). Ext = Externalizing; Int = Internalizing; ThD = Thought Disorders; OF = One-Factor; HO = Higher-Order; OR = Bi-Factor (Orthogonal p-free); OB = Bi-Factor (Oblique p-free); -Ext = Bi-Factor (-p- free Externalizing); -Int = Bi-Factor (-p-free Internalizing); -ThD = Bi-Factor (-p-free Thought Disorders).

**Supplemental Table 6.** Genetic and environmental influences on psychopathology.

|  | rMZ | rDZ | A^2^ (95% CI) | C^2^ (95% CI) | E^2^ (95% CI) |
| --- | --- | --- | --- | --- | --- |
| Correlated-Factors |  |  |  |  |  |
| Externalizing | 0.58 | 0.35 | 0.47 (0.29 – 0.66) | 0.11 (0.00 – 0.28) | 0.42 (0.37 – 0.47) |
| Internalizing | 0.51 | 0.23 | 0.51 (0.43 – 0.58) | 0.00 (0.00 – 0.00) | 0.50 (0.44 – 0.55) |
| Thought Disorders | 0.38 | 0.17 | 0.38 (0.30 – 0.46) | 0.00 (0.00 – 0.00) | 0.63 (0.56 – 0.69) |
| General Factor (‘p’) |  |  |  |  |  |
| One-Factor | 0.53 | 0.27 | 0.51 (0.31 – 0.72) | 0.01 (0.00 – 0.19) | 0.48 (0.42 – 0.53) |
| Higher-Order | 0.51 | 0.24 | 0.51 (0.43 – 0.58) | 0.00 (0.00 – 0.00) | 0.50 (0.44 – 0.55) |
| Bi-Factor (Orthogonal p-free) | 0.51 | 0.23 | 0.51 (0.43 – 0.58) | 0.00 (0.00 – 0.00) | 0.50 (0.44 – 0.55) |
| Bi-Factor (Oblique p-free) | 0.54 | 0.29 | 0.51 (0.31 – 0.71) | 0.03 (0.00 – 0.21) | 0.46 (0.41 – 0.51) |
| Bi-Factor (-p-free Externalizing) | 0.57 | 0.33 | 0.49 (0.30 – 0.68) | 0.08 (0.00 – 0.25) | 0.43 (0.38 – 0.48) |
| Bi-Factor (-p-free Internalizing) | 0.51 | 0.22 | 0.51 (0.43 – 0.58) | 0.00 (0.00 – 0.00) | 0.50 (0.44 – 0.55) |
| Bi-Factor (-p-free Thought Disorders) | 0.42 | 0.19 | 0.42 (0.34 – 0.49) | 0.00 (0.00 – 0.00) | 0.59 (0.52 – 0.65) |
| p-free Factors |  |  |  |  |  |
| Externalizing |  |  |  |  |  |
| Bi-Factor (Orthogonal p-free) | 0.57 | 0.35 | 0.45 (0.27 – 0.64) | 0.12 (0.00 – 0.29) | 0.43 (0.38 – 0.48) |
| Bi-Factor (Oblique p-free) | 0.48 | 0.09 | 0.46 (0.38 – 0.54) | 0.00 (0.00 – 0.00) | 0.55 (0.49 – 0.61) |
| Bi-Factor (-p-free Internalizing) | 0.57 | 0.35 | 0.44 (0.25 – 0.62) | 0.13 (0.00 – 0.30) | 0.43 (0.38 – 0.48) |
| Bi-Factor (-p-free Thought Disorders) | 0.57 | 0.34 | 0.45 (0.27 – 0.64) | 0.12 (0.00 – 0.28) | 0.43 (0.38 – 0.48) |
| Internalizing |  |  |  |  |  |
| Bi-Factor (Orthogonal p-free) | 0.13 | -0.01 | 0.11 (0.03 – 0.19) | 0.00 (0.00 – 0.00) | 0.89 (0.80 – 0.98) |
| Bi-Factor (Oblique p-free) | 0.45 | 0.19 | 0.44 (0.37 – 0.52) | 0.00 (0.00 – 0.00) | 0.56 (0.50 – 0.62) |
| Bi-Factor (-p-free Externalizing) | 0.40 | 0.13 | 0.38 (0.30 – 0.46) | 0.00 (0.00 – 0.00) | 0.62 (0.56 – 0.69) |
| Bi-Factor (-p-free Thought Disorders) | 0.26 | 0.04 | 0.23 (0.15 – 0.31) | 0.00 (0.00 – 0.00) | 0.76 (0.69 – 0.85) |
| Thought Disorders |  |  |  |  |  |
| Bi-Factor (Orthogonal p-free) | 0.13 | 0.02 | 0.11 (0.03 – 0.19) | 0.00 (0.00 – 0.00) | 0.89 (0.80 – 0.98) |
| Bi-Factor (Oblique p-free) | 0.30 | 0.11 | 0.29 (0.21 – 0.36) | 0.00 (0.00 – 0.00) | 0.72 (0.64 – 0.79) |
| Bi-Factor (-p-free Externalizing) | 0.22 | 0.06 | 0.20 (0.12 – 0.28) | 0.00 (0.00 – 0.00) | 0.80 (0.72 – 0.89) |
| Bi-Factor (-p-free Internalizing) | 0.12 | 0.02 | 0.11 (0.03 – 0.19) | 0.00 (0.00 – 0.00) | 0.89 (0.80 – 0.99) |

*Note*. rMZ = correlation between monozygotic twins; rDZ = correlation between dizygotic twins; A^2^ = additive genetic variation; C^2^ = common (shared) environmental variation; E^2^ = specific (non-shared) environmental variation + measurement error.

**Supplemental Table 7.** Relationships (standardized β with 95% Confidence Intervals) between first-order factors and ‘p’ extracted from various models and the nomological net (cf. Figure 1). Model 1 shows associations between each p-factor and the nomological network; Model 2 shows associations between each p-factor and the nomological network after adjusting for the contribution of the three first-order factors in the Correlated Factors Model. All models controlled for sex.

| Relationship with: | Family History | Childhood SES | IQ (age 5) | Low Self- Control | Childhood Maltreatment | Adolescent Victimization | suPAR |
| --- | --- | --- | --- | --- | --- | --- | --- |
| Correlated-Factors |  |  |  |  |  |  |  |
| Externalizing | .16 (.11, .22) | -.16 (-.21, -.11) | -.11 (-.16, -.06) | .34 (.29, .39) | .19 (.13, .25) | .46 (.41, .51) | .11 (.05, .18) |
| Internalizing | .19 (.13, .24) | -.12 (-.17, -.07) | -.07 (-.11, -.02) | .22 (.18, .27) | .17 (.11, .23) | .50 (.45, .56) | .09 (.03, .16) |
| Thought Disorders | .15 (.09, .21) | -.13 (-.18, -.08) | -.10 (-.15, -.06) | .22 (.17, .27) | .15 (.08, .21) | .43 (.36, .50) | .07 (.01, .14) |
| Model 1, p from: |  |  |  |  |  |  |  |
| One Factor | .19 (.13, .24) | -.15 (-.20, -.11) | -.11 (-.16, -.11) | .30 (.25, .35) | .19 (.13, .25) | .52 (.46, .57) | .11 (.04, .18) |
| Higher-Order | .19 (.13, .24) | -.14 (-.19, -.09) | -.09 (-.14, -.09) | .26 (.21, .31) | .18 (.12, .24) | .51 (.46, .57) | .10 (.03, .17) |
| Bi-Factor (Orthogonal p-free) | .18 (.13, .24) | -.14 (-.19, -.09) | -.10 (-.14, -.10) | .27 (.22, .32) | .18 (.12, .24) | .52 (.46, .57) | .10 (.03, .17) |
| Bi-Factor (Oblique p-free) | .17 (.11, .22) | -.15 (-.20, -.10) | -.11 (-.16, -.11) | .36 (.31, .41) | .18 (.12, .23) | .46 (.41, .50) | .09 (.03, .16) |
| Bi-Factor (-p-free Externalizing) | .17 (.12, .23) | -.17 (-.22, -.12) | -.12 (-.17, -.12) | .35 (.30, .40) | .20 (.14, .25) | .49 (.44, .54) | .12 (.05, .19) |
| Bi-Factor (-p-free Internalizing) | .19 (.13, .24) | -.13 (-.18, -.08) | -.07 (-.12, -.08) | .24 (.20, .29) | .17 (.11, .23) | .50 (.45, .56) | .09 (.02, .16) |
| Bi-Factor (-p-free Thought  Disorders) | .16 (.10, .22) | -.14 (-.19, -.09) | -.11 (-.16, -.11) | .25 (.20, .30) | .16 (.09, .22) | .46 (.40, .53) | .09 (.02, .15) |
| Model 2, p from: |  |  |  |  |  |  |  |
| One Factor | -.002 (-.006, .001) | -.001 (-.004, .002) | -.004 (-.007, -.001) | .007 (.003, .011) | .000 (-.003, .004) | -.001 (-.005, .003) | .004 (.000, .008) |
| Higher-Order | .000 (.000, .000) | .000 (.000, .000) | .000 (.000, .000) | .000 (.000, .000) | .000 (.000, .000) | .000 (.000, .000) | .000 (.000, .000) |
| Bi-Factor (Orthogonal p-free) | -.005 (-.012, .002) | -.004 (-.011, .003) | -.012 (-.018, -.005) | .018 (.010, .025) | -.004 (-.011, .003) | .007 (-.001, .016) | .001 (-.006, .009) |
| Bi-Factor (Oblique p-free) | -.005 (-.016, .006) | .009 (-.002, .019) | -.009 (-.020, .001) | .031 (.019, .043) | -.013 (-.024, -.001) | -.019 (-.033, -.006) | -.019 (-.033, -.006) |
| Bi-Factor (-p-free Externalizing) | -.001 (-.005, .002) | -.004 (-.007, .000) | -.005 (-.008, -.002) | .008 (.005, .012) | -.001 (-.005, .002) | -.002 (-.006, .002) | .002 (-.002, .006) |
| Bi-Factor (-p-free Internalizing) | -.002 (-.007, .002) | .002 (-.003, .006) | -.005 (-.010, -.001) | .013 (.008, .018) | -.005 (-.010, .000) | -.006 (-.012, -.001) | -.004 (-.010, .000) |
| Bi-Factor (-p-free Thought  Disorders) | -.003 (-.008, .001) | -.002 (-.006, .002) | -.007 (-.012, -.003) | .008 (.003, .013) | .000 (-.004, .004) | .007 (.002, .012) | .003 (-.001, .008) |

**Supplemental Table 8.** Relationships (standardized β with 95% Confidence Intervals) between first-order factors and p-free factors extracted from various models and the nomological net (cf. Figure 2).

| Factor: | Family History | Childhood SES | IQ (age 5) | Low Self- Control | Childhood Maltreatment | Adolescent Victimization | suPAR |
| --- | --- | --- | --- | --- | --- | --- | --- |
| Externalizing from: |  |  |  |  |  |  |  |
| Correlated-Factors | .16 (.11, .22) | -.16 (-.21, -.11) | -.11 (-.16, -.06) | .34 (.29, .39) | .19 (.13, .25) | .46 (.41, .51) | .11 (.05, .18) |
| Correlated-Factors (adjusted)^a^ | .04 (.00, .07) | -.07 (-.11, -.04) | -.06 (-.10, -.03) | .19 (.16, .23) | .07 (.03, .12) | .15 (.10, .19) | .05 (.01, .10) |
| Bi-Factor (Orthogonal p-free) | .08 (.03, .13) | -.11 (-.16, -.06) | -.08 (-.12, -.03) | .25 (.20, .30) | .12 (.07, .18) | .22 (.17, .28) | .08 (.02, .14) |
| Bi-Factor (Oblique p-free) | .03 (-.02, .09) | -.08 (-.13, -.04) | -.04 (-.09, -.00) | .05 (-.01, .10) | .08 (.02, .14) | .08 (.02, .14) | .10 (.04, .16) |
| Bi-Factor (-p-free Internalizing) | .08 (.02, .13) | -.12 (-.17, -.07) | -.09 (-.13, -.04) | .26 (.21, .31) | .13 (.07, .18) | .13 (.18, .28) | .08 (.02, .14) |
| Bi-Factor (-p-free Thought  Disorders) | .09 (.04, .15) | -.11 (-.16, -.06) | -.07 (-.12, -.02) | .26 (.21, .31) | .13 (.07, .19) | .26 (.20, .31) | .08 (.02, .14) |
| Internalizing from: |  |  |  |  |  |  |  |
| Correlated-Factors | .19 (.13, .24) | -.12 (-.17, -.07) | -.07 (-.11, -.02) | .22 (.18, .27) | .17 (.11, .23) | .50 (.45, .56) | .09 (.03, .16) |
| Correlated-Factors (adjusted)^b^ | .04 (.02, .07) | .01 (-.02, .03) | .04 (.01, .06) | -.02 (-.05, .00) | .02 (.00, .05) | .12 (.09, .15) | .01 (-.02, .04) |
| Bi-Factor (Orthogonal p-free) | .12 (.07, .17) | .00 (-.04, .04) | .06 (.01, .10) | .00 (-.05, .05) | .06 (.01, .10) | .15 (.10, .20) | .01 (-.05, .06) |
| Bi-Factor (Oblique p-free) | .13 (.07, .18) | -.04 (-.09, .01) | .00 (-.04, .05) | .02 (-.03, .07) | .09 (.02, .15) | .33 (.27, .39) | .04 (-.02, .10) |
| Bi-Factor (-p-free Externalizing) | .12 (.07, .18) | -.01 (-.06, .03) | .04 (-.01, .09) | .00 (-.04, .05) | .07 (.02, .13) | .27 (.22, .32) | .02 (-.03, .08) |
| Bi-Factor (-p-free Thought  Disorders) | .13 (.09, .18) | -.02 (-.07, .02) | .05 (.00, .09) | .05 (.00, .10) | .09 (.04, .13) | .25 (.20, .30) | .03 (-.02, .09) |
| Thought Disorders from: |  |  |  |  |  |  |  |
| Correlated-Factors | .15 (.09, .21) | -.13 (-.18, -.08) | -.10 (-.15, -.06) | .22 (.17, .27) | .15 (.08, .21) | .43 (.36, .50) | .07 (.01, .14) |
| Correlated-Factors (adjusted)^c^ | -.01 (-.04, .02) | -.02 (-.05, .00) | -.04 (-.07, -.02) | .02 (-.01, .05) | .00 (-.03, .03) | .00 (-.03, .04) | -.01 (-.04, .03) |
| Bi-Factor (Orthogonal p-free) | .02 (-.03, .08) | -.05 (-.10, -.01) | -.08 (-.12, -.03) | .05 (.00, .10) | .03 (-.03, .10) | .10 (.03, .18) | .01 (-.05, .07) |
| Bi-Factor (Oblique p-free) | .09 (.03, .15) | -.08 (-.13, -.03) | -.07 (-.11, -.02) | .06 (.01, .12) | .08 (.01, .15) | .27 (.20, .35) | .04 (-.02, .11) |
| Bi-Factor (-p-free Externalizing) | .05 (-.01, .10) | -.05 (-.10, .00) | -.07 (-.11, -.02) | .04 (-.01, .09) | .04 (-.03, .11) | .17 (.10, .24) | .01 (-.06, .07) |
| Bi-Factor (-p-free Internalizing) | .02 (-.03, .08) | -.07 (-.11, -.02) | -.09 (-.14, -.04) | .07 (.02, .12) | .04 (-.02, .10) | .12 (.05, .19) | .02 (-.05, .08) |

*Note*. All models controlled for sex. ^a^ Adjusted for Internalizing and Thought Disorders; ^b^ Adjusted for Externalizing and Thought Disorders; ^c^

Adjusted for Externalizing and Internalizing.

**Supplemental Table 9.** Sensitivity analyses: Are the nomological-network results similar whether they are estimated using raw scores, extracted factor scores or structural equation modeling? Results shown are standardized β with 95% Confidence Intervals. Panel A presents results about the nomological network with p from various models. Panel B presents results about the nomological network and Externalizing Disorders from various models. Panel C presents results about the nomological network and Internalizing Disorders from various models. Panel D presents results about the nomological network and Thought Disorders from various models.

| Panel A | Family History | Childhood SES | IQ (age 5) | Low Self-Control | Childhood Maltreatment | Adolescent Victimization | suPAR |
| --- | --- | --- | --- | --- | --- | --- | --- |
| p-from: |  |  |  |  |  |  |  |
| Raw Score | .19 (.14, .25) | -.15 (-.20, -.10) | -.09 (-.14, -.04) | .30 (.25, .34) | .19 (.13, .25) | .52 (.47, .57) | .12 (.05, .19) |
| One-Factor |  |  |  |  |  |  |  |
| Factor Score Regression | .19 (.13, .24) | -.15 (-.20, -.11) | -.11 (-.16, -.11) | .30 (.25, .35) | .19 (.13, .25) | .52 (.46, .57) | .11 (.04, .18) |
| Structural Equation Modeling | .21 (.15, .27) | -.18 (-.23, -.12) | -.12 (-.18, -.07) | .34 (.29, .40) | .22 (.15, .28) | .59 (.54, .64) | .13 (.05, .21) |
| Higher-Order |  |  |  |  |  |  |  |
| Factor Score Regression | .19 (.13, .24) | -.14 (-.19, -.09) | -.09 (-.14, -.09) | .26 (.21, .31) | .18 (.12, .24) | .51 (.46, .57) | .10 (.03, .17) |
| Structural Equation Modeling | .23 (.16, .29) | -.17 (-.23, -.11) | -.11 (-.18, -.04) | .33 (.26, .40) | .22 (.15, .29) | .62 (.57, .68) | .12 (.04, .21) |
| Bi-Factor (Orthogonal p-free) |  |  |  |  |  |  |  |
| Factor Score Regression | .18 (.13, .24) | -.14 (-.19, -.09) | -.10 (-.14, -.10) | .27 (.22, .32) | .18 (.12, .24) | .52 (.46, .57) | .10 (.03, .17) |
| Structural Equation Modeling | .18 (.08, .27) | -.19 (-.32, -.06) | -.23 (-.43, -.03) | .64 (.52, .76) | Did not converge | .58 (.32, .85) | -.39 (-.93, .15) |
| Bi-Factor (Oblique p-free) |  |  |  |  |  |  |  |
| Factor Score Regression | .17 (.11, .22) | -.15 (-.20, -.10) | -.11 (-.16, -.11) | .36 (.31, .41) | .18 (.12, .23) | .46 (.41, .50) | .09 (.03, .16) |
| Structural Equation Modeling | .16 (.09, .23) | -.13 (-.29, .03) | Did not converge | .46 (.39, .52) | .17 (.04, .29) | Did not converge | -.24 (-.42, -.05) |
| Bi-Factor (-p-free Externalizing) |  |  |  |  |  |  |  |
| Factor Score Regression | .17 (.12, .23) | -.17 (-.22, -.12) | -.12 (-.17, -.12) | .35 (.30, .40) | .20 (.14, .25) | .49 (.44, .54) | .12 (.05, .19) |
| Structural Equation Modeling | .17 (.11, .24) | -.19 (-.26, -.13) | -.14 (-.20, -.08) | .43 (.37, .49) | .22 (.15, .29) | .48 (.42, .54) | .14 (.05, .23) |
| Bi-Factor (-p-free Internalizing) |  |  |  |  |  |  |  |
| Factor Score Regression | .19 (.13, .24) | -.13 (-.18, -.08) | -.07 (-.12, -.08) | .24 (.20, .29) | .17 (.11, .23) | .50 (.45, .56) | .09 (.02, .16) |
| Structural Equation Modeling | .21 (.15, .27) | -.12 (-.18, -.06) | -.06 (-.12, .00) | .23 (.17, .29) | .18 (.11, .25) | .56 (.50, .62) | .10 (.02, .19) |
| Bi-Factor (-p-free Thought Disorders) |  |  |  |  |  |  |  |
| Factor Score Regression | .16 (.10, .22) | -.14 (-.19, -.09) | -.11 (-.16, -.11) | .25 (.20, .30) | .16 (.09, .22) | .46 (.40, .53) | .09 (.02, .15) |
| Structural Equation Modeling | .14 (.08, .21) | -.15 (-.21, -.09) | -.15 (-.21, -.10) | .25 (.18, .31) | .15 (.07, .23) | .43 (.36, .51) | .08 (-.02, .18) |

| Panel B | Family History | Childhood SES | IQ (age 5) | Low Self-Control | Childhood Maltreatment | Adolescent Victimization | suPAR |
| --- | --- | --- | --- | --- | --- | --- | --- |
| Externalizing from: |  |  |  |  |  |  |  |
| Raw Score | .14 (.09, .20) | -.16 (-.21, -.11) | -.12 (-.17, -.07) | .35 (.30, .40) | .18 (.12, .23) | .40 (.35, .45) | .12 (.06, .19) |
| Correlated Factors (CF) |  |  |  |  |  |  |  |
| Factor Score Regression | .16 (.11, .22) | -.16 (-.21, -.11) | -.11 (-.16, -.06) | .34 (.29, .39) | .19 (.13, .25) | .46 (.41, .51) | .11 (.05, .18) |
| Structural Equation Modeling | .17 (.10, .23) | -.18 (-.24, -.12) | -.13 (-.19, -.07) | .40 (.34, .46) | .21 (.14, .28) | .47 (.42, .52) | .14 (.05, .22) |
| p-free models |  |  |  |  |  |  |  |
| Adjusted Raw Score^a^ | .07 (.02, .12) | -.11 (-.16, -.07) | -.09 (-.14, -.05) | .28 (.23, .32) | .11 (.06, .16) | .24 (.19, .30) | .09 (.03, .14) |
| Adjusted CF Factor Score Regression^a^ | .04 (.00, .07) | -.07 (-.11, -.04) | -.06 (-.10, -.03) | .19 (.16, .23) | .07 (.03, .12) | .15 (.10, .19) | .05 (.01, .10) |
| Bi-Factor (Orthogonal p-free) |  |  |  |  |  |  |  |
| Factor Score Regression | .08 (.03, .13) | -.11 (-.16, -.06) | -.08 (-.12, -.03) | .25 (.20, .30) | .12 (.07, .18) | .22 (.17, .28) | .08 (.02, .14) |
| Structural Equation Modeling | .06 (-.02, .14) | -.07 (-.18, .05) | .03 (-.13, .19) | -.07 (.26, .12) | Did not converge | .22 (-.01, .45) | .47 (.07, .87) |
| Bi-Factor (Oblique p-free) |  |  |  |  |  |  |  |
| Factor Score Regression | .03 (-.02, .09) | -.08 (-.13, -.04) | -.04 (-.09, -.00) | .05 (-.01, .10) | .08 (.02, .14) | .08 (.02, .14) | .10 (.04, .16) |
| Structural Equation Modeling | .05 (-.07, .17) | -.16 (-.35, .03) | Did not converge | -.01 (-.38, .38) | .13 (-.07, .32) | Did not converge | .27 (.14, .40) |
| Bi-Factor (-p-free Internalizing) |  |  |  |  |  |  |  |
| Factor Score Regression | .08 (.02, .13) | -.12 (-.17, -.07) | -.09 (-.13, -.04) | .26 (.21, .31) | .13 (.07, .18) | .13 (.18, .28) | .08 (.02, .14) |
| Structural Equation Modeling | .05 (-.02, .12) | -.12 (-.19, -.04) | -.09 (-.16, -.03) | .29 (.22, .36) | .11 (.04, .19) | .23 (.15, .32) | .07 (-.02, .16) |
| Bi-Factor (-p-free Thought Disorders) |  |  |  |  |  |  |  |
| Factor Score Regression | .09 (.04, .15) | -.11 (-.16, -.06) | -.07 (-.12, -.02) | .26 (.21, .31) | .13 (.07, .19) | .26 (.20, .31) | .08 (.02, .14) |
| Structural Equation Modeling | .10 (.03, .17) | -.11 (-.18, -.03) | -.04 (-.10, .03) | .30 (.22, .38) | .15 (.06, .23) | .34 (.27, .41) | .10 (-.02, .23) |

| Panel C | Family History | Childhood SES | IQ (age 5) | Low Self-Control | Childhood Maltreatment | Adolescent Victimization | suPAR |
| --- | --- | --- | --- | --- | --- | --- | --- |
| Internalizing from: |  |  |  |  |  |  |  |
| Raw Score | .17 (.12, .23) | -.09 (-.14, -.04) | -.03 (-.08, .02) | .16 (.11, .21) | .15 (.09, .21) | .46 (.41, .51) | .08 (.02, .14) |
| Correlated Factors (CF) |  |  |  |  |  |  |  |
| Factor Score Regression | .19 (.13, .24) | -.12 (-.17, -.07) | -.07 (-.11, -.02) | .22 (.18, .27) | .17 (.11, .23) | .50 (.45, .56) | .09 (.03, .16) |
| Structural Equation Modeling | .21 (.15, .27) | -.12 (-.17, -.06) | -.04 (-.10, .02) | .20 (.14, .26) | .19 (.12, .25) | .57 (.51, .63) | .10 (.02, .18) |
| p-free models |  |  |  |  |  |  |  |
| Adjusted Raw Score^b^ | .09 (.05, .13) | .00 (-.04, .05) | .05 (.01, .08) | -.01 (-.06, .03) | .05 (.01, .10) | .27 (.22, .32) | .02 (-.03, .07) |
| Adjusted CF Factor Score Regression^b^ | .04 (.02, .07) | .01 (-.02, .03) | .04 (.01, .06) | -.02 (-.05, .00) | .02 (.00, .05) | .12 (.09, .15) | .01 (-.02, .04) |
| Bi-Factor (Orthogonal p-free) |  |  |  |  |  |  |  |
| Factor Score Regression | .12 (.07, .17) | .00 (-.04, .04) | .06 (.01, .10) | .00 (-.05, .05) | .06 (.01, .10) | .15 (.10, .20) | .01 (-.05, .06) |
| Structural Equation Modeling | .06 (-.20, .33) | .19 (-.14, .52) | .39 (.07, .70) | -.96 (-1.23, -.69) | Did not converge | -.39 (-.61, -.18) | .70 (.25, 1.16) |
| Bi-Factor (Oblique p-free) |  |  |  |  |  |  |  |
| Factor Score Regression | .13 (.07, .18) | -.04 (-.09, .01) | .00 (-.04, .05) | .02 (-.03, .07) | .09 (.02, .15) | .33 (.27, .39) | .04 (-.02, .10) |
| Structural Equation Modeling | .14 (.06, .21) | -.03 (-.17, .11) | Did not converge | -.16 (-.28, -.05) | .10 (-.03, .22) | Did not converge | .25 (.15, .35) |
| Bi-Factor (-p-free Externalizing) |  |  |  |  |  |  |  |
| Factor Score Regression | .12 (.07, .18) | -.01 (-.06, .03) | .04 (-.01, .09) | .00 (-.04, .05) | .07 (.02, .13) | .27 (.22, .32) | .02 (-.03, .08) |
| Structural Equation Modeling | .13 (.05, .20) | .04 (-.04, .12) | .09 (.01, .16) | -.17 (-.28, -.06) | .04 (-.04, .13) | .38 (.28, .49) | .00 (-.09, .08) |
| Bi-Factor (-p-free Thought Disorders) |  |  |  |  |  |  |  |
| Factor Score Regression | .13 (.09, .18) | -.02 (-.07, .02) | .05 (.00, .09) | .05 (.00, .10) | .09 (.04, .13) | .25 (.20, .30) | .03 (-.02, .09) |
| Structural Equation Modeling | .17 (.10, .24) | .02 (-.07, .11) | .14 (.05, .22) | .02 (-.08, .13) | .10 (.02, .18) | .41 (.26, .57) | .05 (-.07, .17) |

| Panel D | Family History | Childhood SES | IQ (age 5) | Low Self-Control | Childhood Maltreatment | Adolescent Victimization | suPAR |
| --- | --- | --- | --- | --- | --- | --- | --- |
| Thought Disorders from: |  |  |  |  |  |  |  |
| Raw Score | .12 (.06, .17) | -.11 (-.16, -.06) | -.10 (-.14, -.05) | .18 (.12, .23) | .12 (.05, .19) | .33 (.26, .41) | .06 (-.01, .12) |
| Correlated Factors (CF) |  |  |  |  |  |  |  |
| Factor Score Regression | .15 (.09, .21) | -.13 (-.18, -.08) | -.10 (-.15, -.06) | .22 (.17, .27) | .15 (.08, .21) | .43 (.36, .50) | .07 (.01, .14) |
| Structural Equation Modeling | .14 (.08, .20) | -.14 (-.19, -.08) | -.13 (-.18, -.07) | .22 (.16, .27) | .14 (.06, .21) | .42 (.35, .48) | .07 (-.02, .15) |
| p-free models |  |  |  |  |  |  |  |
| Adjusted Raw Score^c^ | .02 (-.02, .06) | -.04 (-.08, .00) | -.07 (-.11, -.03) | .06 (.01, .11) | .03 (-.03, .08) | .10 (.04, .16) | .01 (-.04, .06) |
| Ajusted CF Factor Score Regression^c^ | -.01 (-.04, .02) | -.02 (-.05, .00) | -.04 (-.07, -.02) | .02 (-.01, .05) | .00 (-.03, .03) | .00 (-.03, .04) | -.01 (-.04, .03) |
| Bi-Factor (Orthogonal p-free) |  |  |  |  |  |  |  |
| Factor Score Regression | .02 (-.03, .08) | -.05 (-.10, -.01) | -.08 (-.12, -.03) | .05 (.00, .10) | .03 (-.03, .10) | .10 (.03, .18) | .01 (-.05, .07) |
| Structural Equation Modeling | .00 (-.10, .11) | .02 (-.15, .19) | .09 (-.19, .37) | -.47 (-.68, -.26) | Did not converge | -.04 (-.71, .62) | .49 (-.09, 1.07) |
| Bi-Factor (Oblique p-free) |  |  |  |  |  |  |  |
| Factor Score Regression | .09 (.03, .15) | -.08 (-.13, -.03) | -.07 (-.11, -.02) | .06 (.01, .12) | .08 (.01, .15) | .27 (.20, .35) | .04 (-.02, .11) |
| Structural Equation Modeling | .08 (.01, .15) | -.09 (-.19, .02) | Did not converge | .01 (-.09, .11) | .07 (-.04, .18) | Did not converge | .13 (.03, .23) |
| Bi-Factor (-p-free Externalizing) |  |  |  |  |  |  |  |
| Factor Score Regression | .05 (-.01, .10) | -.05 (-.10, .00) | -.07 (-.11, -.02) | .04 (-.01, .09) | .04 (-.03, .11) | .17 (.10, .24) | .01 (-.06, .07) |
| Structural Equation Modeling | .05 (-.03, .12) | -.03 (-.10, .05) | -.06 (-.12, .01) | -.07 (-.18, .03) | .01 (-.09, .11) | .23 (.13, .33) | -.01 (-.10, .08) |
| Bi-Factor (-p-free Internalizing) |  |  |  |  |  |  |  |
| Factor Score Regression | .02 (-.03, .08) | -.07 (-.11, -.02) | -.09 (-.14, -.04) | .07 (.02, .12) | .04 (-.02, .10) | .12 (.05, .19) | .02 (-.05, .08) |
| Structural Equation Modeling | .02 (-.09, .06) | -.07 (-.14, .01) | -.12 (-.19, -.05) | .07 (-.01, .15) | .01 (-.08, .10) | -.01 (-.19, .17) | -.01 (-.15, .12) |

*Note*. All models controlled for sex. ^a^Adjusted for Internalizing and Thought Disorders; ^b^Adjusted for Externalizing and Thought Disorders; ^c^Adjusted for Externalizing and Internalizing. Cells highlighted in orange indicate that the point estimate of the factor score regression is not within the 95% Confidence Interval of the Structual Equation Modeling estimate. Three structural equation models did not converge; in the Bifactor-Oblique models the problem involved estimating the correlations between the p-free factors and in the Bifactor-Orthogonal model the problem involved the estimation of p-free internalizing on childhood maltreatment. The adjusted Correlated-Factors models could not be estimated within the structural equation modeling framework.


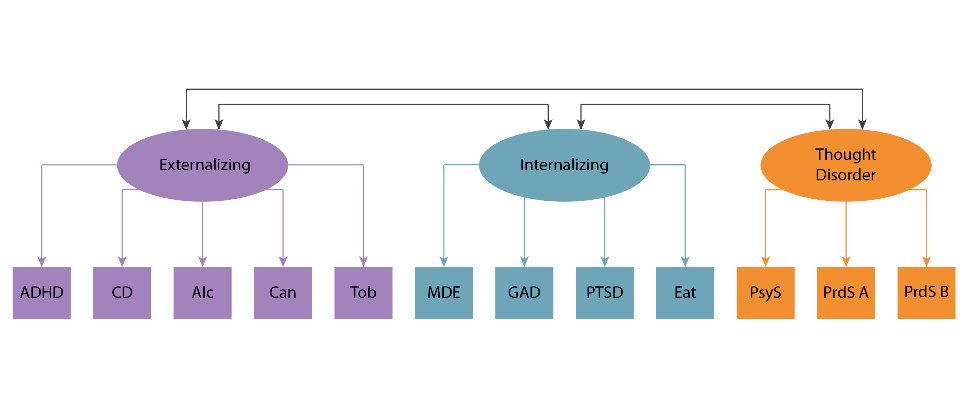

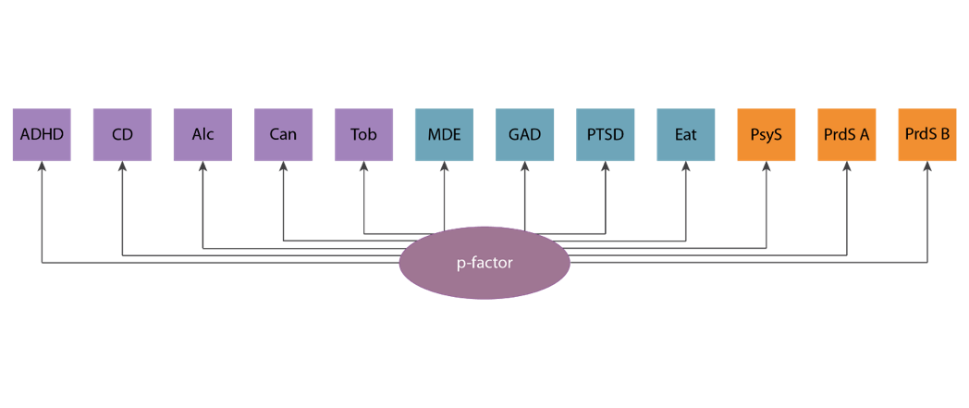
**Supplemental Figure 1.** Comparing different models of the structure of psychopathology. The figure shows structural models that have been used to examine the hierarchical structure of psychopathology. ADHD = attention deficit hyperactivity disorder; CD = conduct disorder; Alc = alcohol dependence; Can = cannabis dependence; Tob = tobacco dependence; MDE = major depressive disorder; GAD = generalized anxiety disorder; PTSD = posttraumatic stress disorder; Eat = eating disorder; PsyS = psychosis symptoms; PrdS A = prodromal psychosis symptoms, parcel A; PrdS B = prodromal psychosis symptoms, parcel B.

| A. Correlated-Factors Model | B. One-Factor Model |
| --- | --- |
| C. Higher-Order Factor Model | D. Bi-Factor Model, Orthogonal p-free Factors |


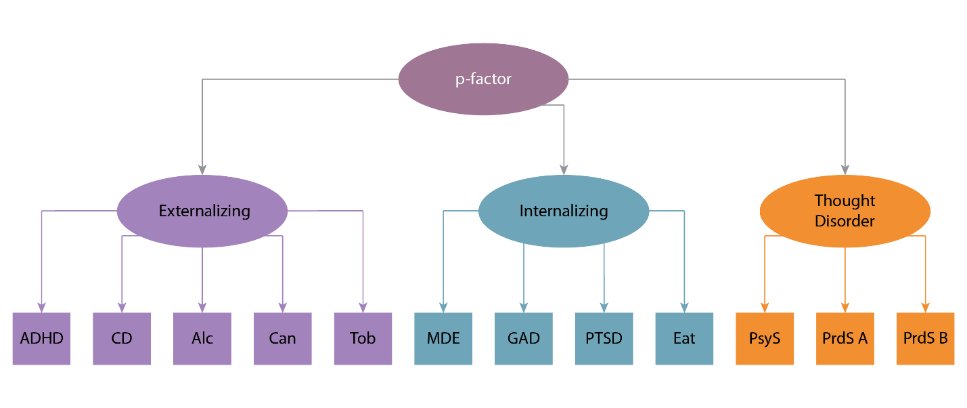

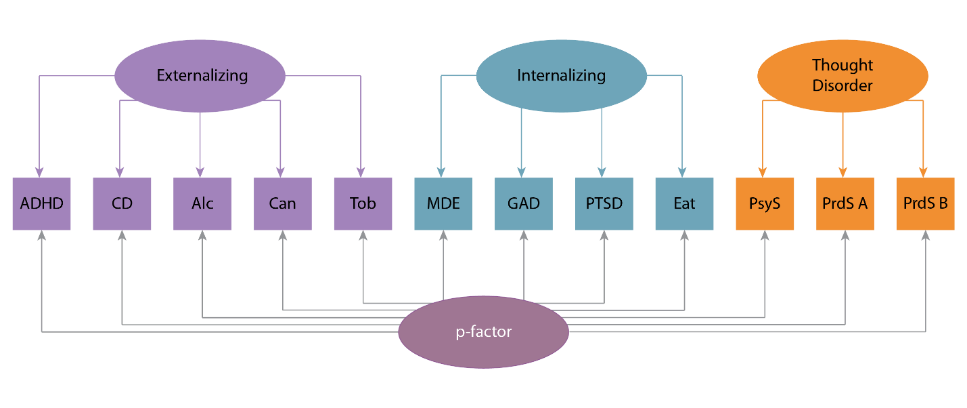


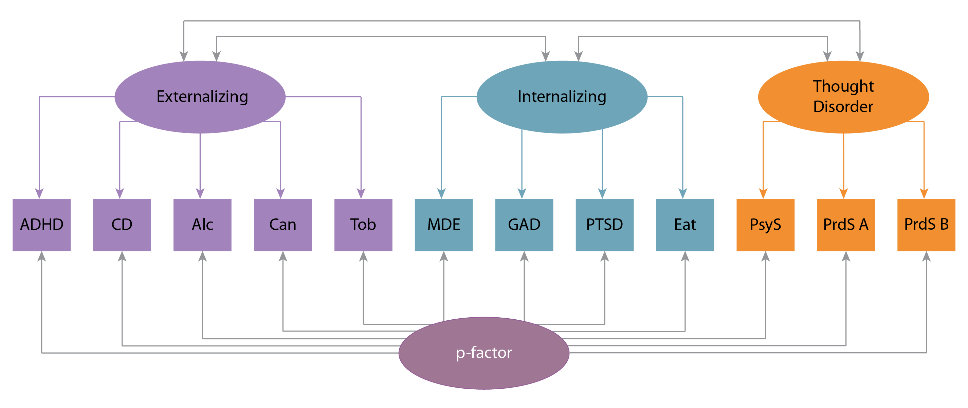

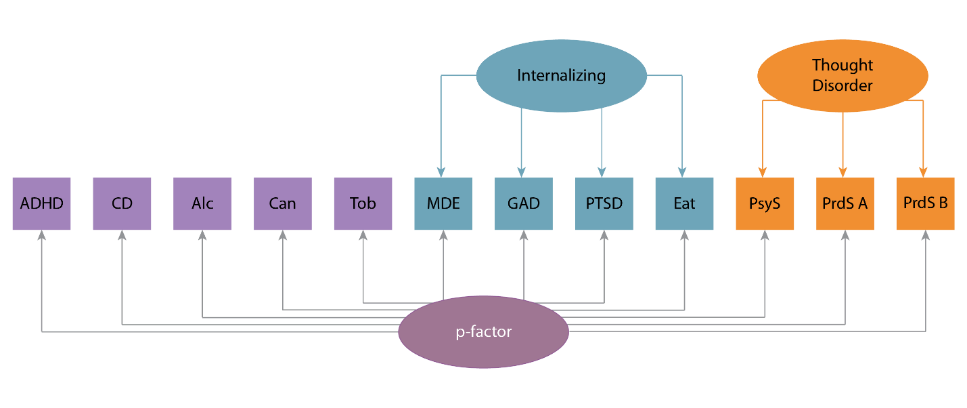
**Supplemental Figure 1. Continued.** Comparing different models of the structure of psychopathology.

| E. Bi-Factor Model, Oblique p-free Factors | F. Bi-Factor Model, -p-free Externalizing |
| --- | --- |
| G. Bi-Factor Model, -p-free Internalizing | H. Bi-Factor Model, -p-free Thought Disorder |


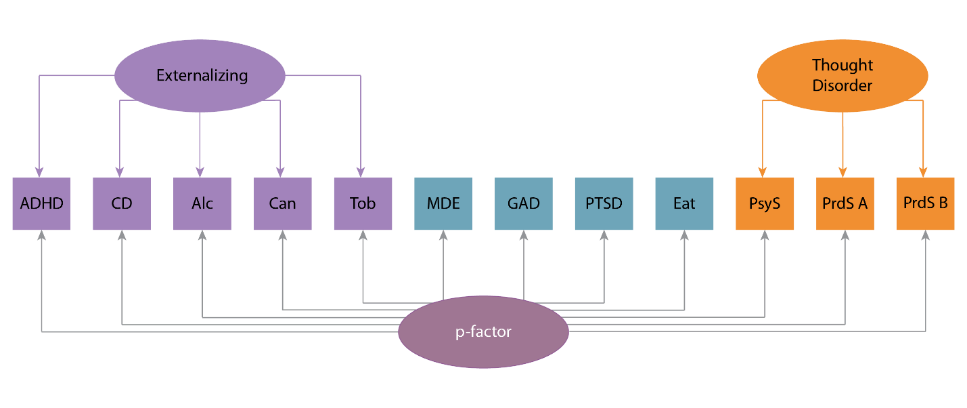

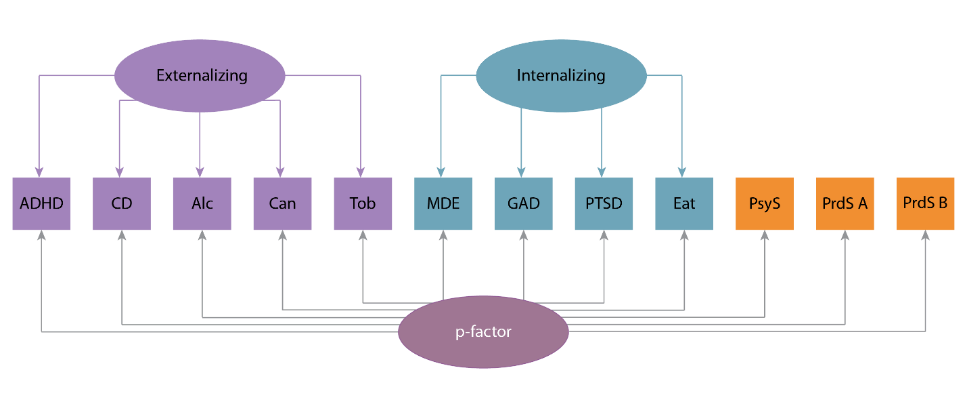


# MPlus Syntax: Correlated-Factors Model

TITLE: Correlated-Factors Model; DATA: FILE IS PCompare_Oct2020.dat; VARIABLE:

NAMES ARE

familyid atwinid

smk18 cd18 adhd18 psy18 alc18 mar18 gad18 mde18 eat18 PTSD18 prodA18 prodB18;

MISSING

ALL (999999);

CLUSTER = familyid; USEVARIABLES ARE

smk18 cd18 adhd18 psy18 alc18 mar18 gad18 mde18 eat18 PTSD18 prodA18 prodB18;

IDVARIABLE IS

atwinid;

ANALYSIS:

TYPE = COMPLEX; ESTIMATOR = MLR;

MODEL = NOCOVARIANCES;

MODEL:

ext BY adhd18* alc18 mar18 smk18 cd18; int BY gad18* mde18 eat18 PTSD18;

thd BY psy18* prodA18 prodB18;

[ext@0 int@0 thd@0]; ext@1 int@1 thd@1;

ext WITH int thd; int WITH thd;

OUTPUT: SAMPSTAT STANDARDIZED FSDETERMINACY SAVEDATA:

FILE = CorrelatedFactors.dat;

SAVE = FSCORES; MISSFLAG = 9999;

# MPlus Syntax: One-Factor Model

TITLE: One-Factor Model;

DATA: FILE IS PCompare_Oct2020.dat; VARIABLE:

NAMES ARE

familyid atwinid

smk18 cd18 adhd18 psy18 alc18 mar18 gad18 mde18 eat18 PTSD18 prodA18 prodB18;

MISSING

ALL (999999);

CLUSTER = familyid; USEVARIABLES ARE

smk18 cd18 adhd18 psy18 alc18 mar18 gad18 mde18 eat18 PTSD18 prodA18 prodB18;

IDVARIABLE IS

atwinid;

ANALYSIS:

TYPE = COMPLEX; ESTIMATOR = MLR;

MODEL = NOCOVARIANCES;

MODEL:

P BY adhd18* alc18 mar18 smk18 cd18 gad18 mde18 eat18 PTSD18 psy18 prodA18 prodB18;

[P@0];

p@1;

OUTPUT: SAMPSTAT STANDARDIZED FSDETERMINACY SAVEDATA:

FILE = OneFactor.dat; SAVE = FSCORES; MISSFLAG = 9999;

# MPlus Syntax: Higher-Order Factor Model

TITLE: Higher-Order Factor Model; DATA: FILE IS PCompare_Oct2020.dat; VARIABLE:

NAMES ARE

familyid atwinid

smk18 cd18 adhd18 psy18 alc18 mar18 gad18 mde18 eat18 PTSD18 prodA18 prodB18;

MISSING

ALL (999999);

CLUSTER = familyid; USEVARIABLES ARE

smk18 cd18 adhd18 psy18 alc18 mar18 gad18 mde18 eat18 PTSD18 prodA18 prodB18;

IDVARIABLE IS

atwinid;

ANALYSIS:

TYPE = COMPLEX; ESTIMATOR = MLR;

MODEL = NOCOVARIANCES;

MODEL:

ext BY adhd18* alc18 mar18 smk18 cd18; int BY gad18* mde18 eat18 PTSD18;

thd BY psy18* prodA18 prodB18; p BY ext* int thd;

[ext@0 int@0 thd@0 p@0]; ext@1 int@1 thd@1 p@1;

OUTPUT: SAMPSTAT STANDARDIZED FSDETERMINACY SAVEDATA:

FILE = HigherOrder.dat;

SAVE = FSCORES; MISSFLAG = 9999;

# MPlus Syntax: Bi-Factor (Orthogonal p-free)

TITLE: Bi-Factor (Orthogonal p-free); DATA: FILE IS PCompare_Oct2020.dat; VARIABLE:

NAMES ARE

familyid atwinid

smk18 cd18 adhd18 psy18 alc18 mar18 gad18 mde18 eat18 PTSD18 prodA18 prodB18;

MISSING

ALL (999999);

CLUSTER = familyid; USEVARIABLES ARE

smk18 cd18 adhd18 psy18 alc18 mar18 gad18 mde18 eat18 PTSD18 prodA18 prodB18;

IDVARIABLE IS

atwinid;

ANALYSIS:

TYPE = COMPLEX; ESTIMATOR = MLR;

MODEL = NOCOVARIANCES;

MODEL:

ext BY adhd18* alc18 mar18 smk18 cd18; int BY gad18* mde18 eat18 PTSD18;

thd BY psy18* prodA18 prodB18;

p BY adhd18* alc18 mar18 smk18 cd18 gad18 mde18 eat18 PTSD18 psy18 prodA18 prodB18;

[ext@0 int@0 thd@0 p@0]; ext@1 int@1 thd@1 p@1;

ext WITH int@0 thd@0; int WITH thd@0;

P WITH ext@0 int@0 thd@0;

OUTPUT: SAMPSTAT STANDARDIZED FSDETERMINACY SAVEDATA:

FILE = BiFactorOrtho.dat; SAVE = FSCORES; MISSFLAG = 9999;

# MPlus Syntax: Bi-Factor (Oblique p-free)

TITLE: Bi-Factor (Oblique p-free); DATA: FILE IS PCompare_Oct2020.dat; VARIABLE:

NAMES ARE

familyid atwinid

smk18 cd18 adhd18 psy18 alc18 mar18 gad18 mde18 eat18 PTSD18 prodA18 prodB18;

MISSING

ALL (999999);

CLUSTER = familyid; USEVARIABLES ARE

smk18 cd18 adhd18 psy18 alc18 mar18 gad18 mde18 eat18 PTSD18 prodA18 prodB18;

IDVARIABLE IS

atwinid;

ANALYSIS:

TYPE = COMPLEX; ESTIMATOR = MLR;

MODEL = NOCOVARIANCES;

MODEL:

ext BY adhd18* alc18 mar18 smk18 cd18; int BY gad18* mde18 eat18 PTSD18;

thd BY psy18* prodA18 prodB18;

p BY adhd18* alc18 mar18 smk18 cd18 gad18 mde18 eat18 PTSD18 psy18 prodA18 prodB18;

[ext@0 int@0 thd@0 p@0]; ext@1 int@1 thd@1 p@1;

ext WITH int thd; int WITH thd;

P WITH ext@0 int@0 thd@0;

OUTPUT: SAMPSTAT STANDARDIZED FSDETERMINACY SAVEDATA:

FILE = BiFactorOblique.dat; SAVE = FSCORES;

MISSFLAG = 9999;

# MPlus Syntax: Bi-Factor (-p-free Externalizing)

TITLE: Bi-Factor (-p-free Externalizing); DATA: FILE IS PCompare_Oct2020.dat; VARIABLE:

NAMES ARE

familyid atwinid

smk18 cd18 adhd18 psy18 alc18 mar18 gad18 mde18 eat18 PTSD18 prodA18 prodB18;

MISSING

ALL (999999);

CLUSTER = familyid; USEVARIABLES ARE

smk18 cd18 adhd18 psy18 alc18 mar18 gad18 mde18 eat18 PTSD18 prodA18 prodB18;

IDVARIABLE IS

atwinid;

ANALYSIS:

TYPE = COMPLEX; ESTIMATOR = MLR;

MODEL = NOCOVARIANCES;

MODEL:

int BY gad18* mde18 eat18 PTSD18; thd BY psy18* prodA18 prodB18;

p BY adhd18* alc18 mar18 smk18 cd18 gad18 mde18 eat18 PTSD18 psy18 prodA18 prodB18;

[int@0 thd@0 p@0]; int@1 thd@1 p@1;

int WITH thd@0;

P WITH int@0 thd@0;

OUTPUT: SAMPSTAT STANDARDIZED FSDETERMINACY SAVEDATA:

FILE = BiFactorS-Ext.dat;

SAVE = FSCORES; MISSFLAG = 9999;

# MPlus Syntax: Bi-Factor (-p-free Internalizing)

TITLE: Bi-Factor (-p-free Internalizing); DATA: FILE IS PCompare_Oct2020.dat; VARIABLE:

NAMES ARE

familyid atwinid

smk18 cd18 adhd18 psy18 alc18 mar18 gad18 mde18 eat18 PTSD18 prodA18 prodB18;

MISSING

ALL (999999);

CLUSTER = familyid; USEVARIABLES ARE

smk18 cd18 adhd18 psy18 alc18 mar18 gad18 mde18 eat18 PTSD18 prodA18 prodB18;

IDVARIABLE IS

atwinid;

ANALYSIS:

TYPE = COMPLEX; ESTIMATOR = MLR;

MODEL = NOCOVARIANCES;

MODEL:

ext BY adhd18* alc18 mar18 smk18 cd18; thd BY psy18* prodA18 prodB18;

p BY adhd18* alc18 mar18 smk18 cd18 gad18 mde18 eat18 PTSD18 psy18 prodA18 prodB18;

[ext@0 thd@0 p@0]; ext@1 thd@1 p@1;

ext WITH thd@0;

P WITH ext@0 thd@0;

OUTPUT: SAMPSTAT STANDARDIZED FSDETERMINACY SAVEDATA:

FILE = BiFactorS-Int.dat;

SAVE = FSCORES; MISSFLAG = 9999;

# MPlus Syntax: Bi-Factor (-p-free Thought Disorders)

TITLE: Bi-Factor (-p-free Thought Disorders); DATA: FILE IS PCompare_Oct2020.dat; VARIABLE:

NAMES ARE

familyid atwinid

smk18 cd18 adhd18 psy18 alc18 mar18 gad18 mde18 eat18 PTSD18 prodA18 prodB18;

MISSING

ALL (999999);

CLUSTER = familyid; USEVARIABLES ARE

smk18 cd18 adhd18 psy18 alc18 mar18 gad18 mde18 eat18 PTSD18 prodA18 prodB18;

IDVARIABLE IS

atwinid;

ANALYSIS:

TYPE = COMPLEX; ESTIMATOR = MLR;

MODEL = NOCOVARIANCES;

MODEL:

ext BY adhd18* alc18 mar18 smk18 cd18; int BY gad18* mde18 eat18 PTSD18;

p BY adhd18* alc18 mar18 smk18 cd18 gad18 mde18 eat18 PTSD18 psy18 prodA18 prodB18;

[ext@0 int@0 p@0]; ext@1 int@1 p@1;

ext WITH int@0;

P WITH ext@0 int@0;

OUTPUT: SAMPSTAT STANDARDIZED FSDETERMINACY SAVEDATA:

FILE = BiFactorS-ThD.dat;

SAVE = FSCORES; MISSFLAG = 9999;
