# Supplementary material for: The General Factor of Psychopathology (p): Choosing Among Competing Models and Interpreting p
Source: Clin Psychol Sci. 2023 May 3;12(1):53–82. doi: 10.1177/21677026221147872 (PMC10794018; doi:10.1177/21677026221147872)
Supplement: sj-pdf-2-cpx-10.1177_21677026221147872 – Supplemental material for The General Factor of Psychopathology (p): Choosing Among Competing Models and Interpreting p [file sj-pdf-2-cpx-10.1177_21677026221147872.pdf]

The general factor of psychopathology (p): Choosing among competing models and interpreting p

Caspi, Houts, Fisher, Danese, & Moffitt

Supplemental Information: MPlus Output

Correlated-Factors Model 2

One-Factor Model 13

Higher-Order Factor Model 23

Bi-Factor (Orthogonal p-free) Model 34

Bi-Factor (Oblique p-free) Model 46

Bi-Factor (-p-free Externalizing) Model 58

Bi-Factor (-p-free Internalizing) Model 69

Bi-Factor (-p-free Thought Disorder) Model 80

```
Mplus VERSION 8.7
MUTHEN & MUTHEN
10/27/2022  10:12 AM

INPUT INSTRUCTIONS

  TITLE: E-Risk P Compare (Correlated-Factors Model);

  DATA:  FILE IS PCompare_Oct2020.dat;

  VARIABLE:
          NAMES ARE
              familyid atwinid rorder sex zygosity iqe5 seswq35 lowsc harm
              polyv512 aces512 famhist smk18 cd18 adhd18 psy18
              polyv18 CRP18 IL6_18 suPAR iq18 fsiq18
              alc18 mar18 gad18 mde18 eat18 ptsd18 prod18 prodA18 prodB18;

          MISSING
              ALL (999999);

          CLUSTER = familyid;

          USEVARIABLES ARE
              smk18 cd18 adhd18 psy18 alc18 mar18 gad18 mde18 eat18 PTSD18
              prodA18 prodB18;

          IDVARIABLE IS
              atwinid;

  ANALYSIS:
      TYPE      = COMPLEX;
      ESTIMATOR = MLR;
      MODEL     = NOCOVARIANCES;

  MODEL:

      ext BY adhd18* alc18 mar18 smk18 cd18;
      int BY gad18* mde18 eat18 PTSD18;
      thd BY psy18* prodA18 prodB18;

      [ext@0 int@0 thd@0];
       ext@1 int@1 thd@1;

      ext WITH int thd;
      int WITH thd;

  OUTPUT: SAMPSTAT STANDARDIZED FSDETERMINACY PATTERNS;

  !SAVEDATA:
  !    FILE     = CorrelatedFactors.dat;
  !    SAVE     = FSCORES;
  !    MISSFLAG = 9999;

*** WARNING
  Data set contains cases with missing on all variables.
  These cases were not included in the analysis.
  Number of cases with missing on all variables:  166
   1 WARNING(S) FOUND IN THE INPUT INSTRUCTIONS

E-Risk P Compare (Correlated-Factors Model);

SUMMARY OF ANALYSIS

Number of groups                                                 1
Number of observations                                        2066

Number of dependent variables                                   12
Number of independent variables                                  0
Number of continuous latent variables                            3
```

Observed dependent variables

Continuous

SMK18	CD18	ADHD18	PSY18	ALC18	MAR18
GAD18	MDE18	EAT18	PTSD18	PRODA18	PRODB18

Continuous latent variables

EXT	INT	THD

Variables with special functions

Cluster variable	FAMILYID
ID variable	ATWINID

Estimator	MLR
Information matrix	OBSERVED
Maximum number of iterations	1000
Convergence criterion	0.500D-04
Maximum number of steepest descent iterations	20
Maximum number of iterations for H1	2000
Convergence criterion for H1	0.100D-03

Input data file(s)
PCompare_Oct2020.dat

Input data format FREE

SUMMARY OF DATA

Number of missing data patterns	13
Number of clusters	1044

SUMMARY OF MISSING DATA PATTERNS

MISSING DATA PATTERNS (x = not missing)

	1	2	3	4	5	6	7	8	9	10	11	12	13
SMK18	x	x	x	x	x	x	x	x	x	x	x		
CD18	x	x	x	x	x	x						x	x
ADHD18	x	x	x	x	x		x	x	x			x	x
PSY18	x	x	x	x	x	x	x	x		x		x	x
ALC18	x	x	x	x	x	x	x		x		x	x	
MAR18	x	x	x	x	x	x	x	x	x	x	x	x	x
GAD18	x	x	x	x	x	x	x	x	x	x	x	x	x
MDE18	x	x	x	x		x	x	x	x		x	x	x
EAT18	x	x	x		x	x	x	x	x		x	x	x
PTSD18	x	x		x	x	x	x	x	x	x	x	x	x
PRODA18	x		x	x	x	x	x	x		x		x	x
PRODB18	x		x	x	x	x	x	x		x		x	x

MISSING DATA PATTERN FREQUENCIES

Pattern	Frequency	Pattern	Frequency	Pattern	Frequency
1	2040	6	3	11	1
2	1	7	8	12	3
3	2	8	1	13	1
4	1	9	2		
5	2	10	1		

COVARIANCE COVERAGE OF DATA

Minimum covariance coverage value 0.100

PROPORTION OF DATA PRESENT

Covariance Coverage

	SMK18	CD18	ADHD18	PSY18	ALC18
SMK18	0.998				
CD18	0.992	0.994			
ADHD18	0.996	0.992	0.998		
PSY18	0.997	0.994	0.997	0.999	
ALC18	0.997	0.993	0.997	0.997	0.999
MAR18	0.998	0.994	0.998	0.999	0.999
GAD18	0.998	0.994	0.998	0.999	0.999
MDE18	0.997	0.993	0.997	0.997	0.998
EAT18	0.997	0.993	0.997	0.998	0.998
PTSD18	0.997	0.993	0.997	0.998	0.998
PRODA18	0.996	0.993	0.996	0.998	0.997
PRODB18	0.996	0.993	0.996	0.998	0.997

Covariance Coverage

	MAR18	GAD18	MDE18	EAT18	PTSD18
MAR18	1.000				
GAD18	1.000	1.000			
MDE18	0.999	0.999	0.999		
EAT18	0.999	0.999	0.998	0.999	
PTSD18	0.999	0.999	0.998	0.998	0.999
PRODA18	0.998	0.998	0.997	0.997	0.997
PRODB18	0.998	0.998	0.997	0.997	0.997

Covariance Coverage

	PRODA18	PRODB18
PRODA18	0.998	
PRODB18	0.998	0.998

SAMPLE STATISTICS

ESTIMATED SAMPLE STATISTICS

Means

SMK18	CD18	ADHD18	PSY18	ALC18
0.672	2.123	5.788	0.044	1.125

Means

MAR18	GAD18	MDE18	EAT18	PTSD18
0.335	1.134	1.809	0.453	1.365

Means

PRODA18	PRODB18
0.273	0.247

Covariances

	SMK18	CD18	ADHD18	PSY18	ALC18
SMK18	2.630				
CD18	1.183	5.205			
ADHD18	1.560	4.202	18.393		
PSY18	0.044	0.076	0.167	0.094	
ALC18	0.522	1.417	2.176	0.022	2.818
MAR18	0.839	1.260	1.265	0.048	0.442
GAD18	0.292	0.672	2.373	0.109	0.471
MDE18	0.933	1.595	4.348	0.153	1.282
EAT18	0.212	0.341	1.041	0.036	0.299
PTSD18	0.938	1.135	2.751	0.158	0.660
PRODA18	0.181	0.372	0.881	0.081	0.134

PRODB18	0.150	0.437	0.892	0.069	0.217

Covariances

	MAR18	GAD18	MDE18	EAT18	PTSD18
MAR18	1.891				
GAD18	0.241	4.627			
MDE18	0.689	2.941	8.835		
EAT18	0.107	0.505	0.917	0.779	
PTSD18	0.366	1.711	3.372	0.637	11.012
PRODA18	0.201	0.488	0.772	0.177	0.882
PRODB18	0.233	0.489	0.761	0.141	0.690

Covariances

	PRODA18	PRODB18
PRODA18	0.589	
PRODB18	0.293	0.584

Correlations

	SMK18	CD18	ADHD18	PSY18	ALC18
SMK18	1.000				
CD18	0.320	1.000			
ADHD18	0.224	0.429	1.000		
PSY18	0.089	0.109	0.127	1.000	
ALC18	0.192	0.370	0.302	0.044	1.000
MAR18	0.376	0.402	0.215	0.114	0.192
GAD18	0.084	0.137	0.257	0.166	0.131
MDE18	0.194	0.235	0.341	0.168	0.257
EAT18	0.148	0.169	0.275	0.135	0.202
PTSD18	0.174	0.150	0.193	0.156	0.118
PRODA18	0.145	0.212	0.268	0.346	0.104
PRODB18	0.121	0.251	0.272	0.294	0.169

Correlations

	MAR18	GAD18	MDE18	EAT18	PTSD18
MAR18	1.000				
GAD18	0.081	1.000			
MDE18	0.169	0.460	1.000		
EAT18	0.088	0.266	0.350	1.000	
PTSD18	0.080	0.240	0.342	0.218	1.000
PRODA18	0.191	0.296	0.339	0.262	0.346
PRODB18	0.222	0.298	0.335	0.210	0.272

Correlations

	PRODA18	PRODB18
PRODA18	1.000	
PRODB18	0.500	1.000

MAXIMUM LOG-LIKELIHOOD VALUE FOR THE UNRESTRICTED (H1) MODEL IS -42633.558

UNIVARIATE SAMPLE STATISTICS

UNIVARIATE HIGHER-ORDER MOMENT DESCRIPTIVE STATISTICS

Variable/ Sample Size	Mean/ Variance	Skewness/ Kurtosis	Minimum/ Maximum	% with Min/Max	Percentiles 20%/60%	40%/80%	Median
SMK18	0.670	2.585	0.000	80.94%	0.000	0.000	0.000
2062.000	2.625	6.106	10.000	0.05%	0.000	0.000	
CD18	2.121	1.262	0.000	30.54%	0.000	1.000	1.000
2053.000	5.209	1.205	11.000	0.24%	2.000	4.000	
ADHD18	5.787	0.510	0.000	10.92%	2.000	4.000	5.000
2061.000	18.399	-0.528	18.000	0.44%	7.000	10.000	
PSY18	0.044	9.848	0.000	97.14%	0.000	0.000	0.000
2063.000	0.094	125.741	6.000	0.05%	0.000	0.000	

```
     ALC18                1.125       1.949       0.000   53.76%       0.000      0.000      0.000
            2063.000      2.818       4.436      11.000    0.10%       1.000      2.000
     MAR18                0.335       4.751       0.000   92.40%       0.000      0.000      0.000
            2066.000      1.891      23.548      10.000    0.44%       0.000      0.000
     GAD18                1.134       1.593       0.000   75.46%       0.000      0.000      0.000
            2066.000      4.627       0.931       7.000    3.05%       0.000      3.000
     MDE18                1.806       1.273       0.000   68.93%       0.000      0.000      0.000
            2063.000      8.828      -0.017       9.000    3.34%       0.000      5.000
     EAT18                0.453       2.416       0.000   71.71%       0.000      0.000      0.000
            2064.000      0.779       6.256       5.000    0.44%       0.000      1.000
     PTSD18               1.365       2.642       0.000   79.41%       0.000      0.000      0.000
            2064.000     11.014       6.296      17.000    0.24%       0.000      1.000
     PRODA18              0.273       3.450       0.000   85.55%       0.000      0.000      0.000
            2062.000      0.588      14.002       6.000    0.19%       0.000      0.000
     PRODB18              0.247       3.734       0.000   87.83%       0.000      0.000      0.000
            2062.000      0.584      15.720       6.000    0.19%       0.000      0.000


THE MODEL ESTIMATION TERMINATED NORMALLY

MODEL FIT INFORMATION

Number of Free Parameters                       39

Loglikelihood

          H0 Value                      -42854.585
          H0 Scaling Correction Factor      4.4590
            for MLR
          H1 Value                      -42633.558
          H1 Scaling Correction Factor      3.0107
            for MLR

Information Criteria

          Akaike (AIC)                   85787.170
          Bayesian (BIC)                 86006.871
          Sample-Size Adjusted BIC       85882.965
            (n* = (n + 2) / 24)

Chi-Square Test of Model Fit

          Value                            232.264*
          Degrees of Freedom                    51
          P-Value                           0.0000
          Scaling Correction Factor         1.9032
            for MLR

*   The chi-square value for MLM, MLMV, MLR, ULSMV, WLSM and WLSMV cannot be used
    for chi-square difference testing in the regular way.  MLM, MLR and WLSM
    chi-square difference testing is described on the Mplus website.  MLMV, WLSMV,
    and ULSMV difference testing is done using the DIFFTEST option.

RMSEA (Root Mean Square Error Of Approximation)

          Estimate                           0.041
          90 Percent C.I.                    0.036  0.047
          Probability RMSEA <= .05           0.995

CFI/TLI

          CFI                                0.923
          TLI                                0.900

Chi-Square Test of Model Fit for the Baseline Model

          Value                           2418.883
          Degrees of Freedom                    66
          P-Value                           0.0000
```

```
SRMR (Standardized Root Mean Square Residual)

          Value                              0.042


MODEL RESULTS
                                                    Two-Tailed
                    Estimate       S.E.  Est./S.E.    P-Value

 EXT      BY
    ADHD18             2.550      0.113     22.616      0.000
    ALC18              0.809      0.063     12.753      0.000
    MAR18              0.703      0.080      8.741      0.000
    SMK18              0.755      0.061     12.423      0.000
    CD18               1.631      0.080     20.366      0.000

 INT      BY
    GAD18              1.249      0.065     19.105      0.000
    MDE18              2.184      0.089     24.581      0.000
    EAT18              0.422      0.032     13.052      0.000
    PTSD18             1.597      0.145     10.982      0.000

 THD      BY
    PSY18              0.132      0.026      5.093      0.000
    PRODA18            0.567      0.042     13.586      0.000
    PRODB18            0.526      0.040     13.087      0.000

 EXT      WITH
    INT                0.539      0.038     14.172      0.000
    THD                0.483      0.041     11.698      0.000

 INT      WITH
    THD                0.689      0.037     18.832      0.000

 Means
    EXT                0.000      0.000    999.000    999.000
    INT                0.000      0.000    999.000    999.000
    THD                0.000      0.000    999.000    999.000

 Intercepts
    SMK18              0.672      0.044     15.379      0.000
    CD18               2.124      0.061     34.833      0.000
    ADHD18             5.789      0.105     54.980      0.000
    PSY18              0.044      0.007      6.313      0.000
    ALC18              1.125      0.042     26.782      0.000
    MAR18              0.335      0.034      9.757      0.000
    GAD18              1.134      0.052     22.005      0.000
    MDE18              1.809      0.072     25.033      0.000
    EAT18              0.453      0.022     20.715      0.000
    PTSD18             1.365      0.082     16.737      0.000
    PRODA18            0.273      0.018     15.101      0.000
    PRODB18            0.247      0.018     13.690      0.000

 Variances
    EXT                1.000      0.000    999.000    999.000
    INT                1.000      0.000    999.000    999.000
    THD                1.000      0.000    999.000    999.000

 Residual Variances
    SMK18              2.059      0.158     13.028      0.000
    CD18               2.548      0.197     12.947      0.000
    ADHD18            11.894      0.533     22.296      0.000
    PSY18              0.076      0.018      4.186      0.000
    ALC18              2.163      0.125     17.330      0.000
    MAR18              1.397      0.145      9.635      0.000
    GAD18              3.067      0.162     18.938      0.000
    MDE18              4.067      0.322     12.634      0.000
    EAT18              0.601      0.043     13.917      0.000
    PTSD18             8.462      0.532     15.895      0.000
    PRODA18            0.267      0.030      8.826      0.000
```

```
   PRODB18            0.307      0.033      9.432      0.000

QUALITY OF NUMERICAL RESULTS

     Condition Number for the Information Matrix              0.468E-03
       (ratio of smallest to largest eigenvalue)

STANDARDIZED MODEL RESULTS

STDYX Standardization
                                                    Two-Tailed
                    Estimate       S.E.  Est./S.E.    P-Value

 EXT      BY
    ADHD18             0.595      0.023     25.703      0.000
    ALC18              0.482      0.030     16.113      0.000
    MAR18              0.511      0.034     15.105      0.000
    SMK18              0.465      0.031     15.216      0.000
    CD18               0.715      0.027     26.791      0.000

 INT      BY
    GAD18              0.581      0.025     22.905      0.000
    MDE18              0.735      0.025     29.704      0.000
    EAT18              0.478      0.029     16.521      0.000
    PTSD18             0.481      0.033     14.395      0.000

 THD      BY
    PSY18              0.433      0.049      8.927      0.000
    PRODA18            0.739      0.033     22.598      0.000
    PRODB18            0.688      0.032     21.288      0.000

 EXT      WITH
    INT                0.539      0.038     14.172      0.000
    THD                0.483      0.041     11.698      0.000

 INT      WITH
    THD                0.689      0.037     18.832      0.000

 Means
    EXT                0.000      0.000    999.000    999.000
    INT                0.000      0.000    999.000    999.000
    THD                0.000      0.000    999.000    999.000

 Intercepts
    SMK18              0.414      0.014     29.282      0.000
    CD18               0.931      0.018     51.090      0.000
    ADHD18             1.350      0.023     57.504      0.000
    PSY18              0.144      0.011     12.741      0.000
    ALC18              0.670      0.015     43.283      0.000
    MAR18              0.244      0.012     20.944      0.000
    GAD18              0.527      0.014     36.592      0.000
    MDE18              0.609      0.015     39.369      0.000
    EAT18              0.513      0.013     39.864      0.000
    PTSD18             0.411      0.012     33.053      0.000
    PRODA18            0.356      0.012     29.064      0.000
    PRODB18            0.323      0.012     27.106      0.000

 Variances
    EXT                1.000      0.000    999.000    999.000
    INT                1.000      0.000    999.000    999.000
    THD                1.000      0.000    999.000    999.000

 Residual Variances
    SMK18              0.783      0.028     27.504      0.000
    CD18               0.489      0.038     12.831      0.000
    ADHD18             0.646      0.028     23.501      0.000
    PSY18              0.812      0.042     19.330      0.000
    ALC18              0.768      0.029     26.623      0.000
    MAR18              0.739      0.035     21.374      0.000
    GAD18              0.663      0.029     22.514      0.000
```

MDE18	0.460	0.036	12.665	0.000
EAT18	0.771	0.028	27.830	0.000
PTSD18	0.768	0.032	23.877	0.000
PRODA18	0.454	0.048	9.410	0.000
PRODB18	0.526	0.045	11.826	0.000

STDY Standardization

	Estimate	S.E.	Est./S.E.	Two-Tailed P-Value
**EXT BY**				
ADHD18	0.595	0.023	25.703	0.000
ALC18	0.482	0.030	16.113	0.000
MAR18	0.511	0.034	15.105	0.000
SMK18	0.465	0.031	15.216	0.000
CD18	0.715	0.027	26.791	0.000
**INT BY**				
GAD18	0.581	0.025	22.905	0.000
MDE18	0.735	0.025	29.704	0.000
EAT18	0.478	0.029	16.521	0.000
PTSD18	0.481	0.033	14.395	0.000
**THD BY**				
PSY18	0.433	0.049	8.927	0.000
PRODA18	0.739	0.033	22.598	0.000
PRODB18	0.688	0.032	21.288	0.000
**EXT WITH**				
INT	0.539	0.038	14.172	0.000
THD	0.483	0.041	11.698	0.000
**INT WITH**				
THD	0.689	0.037	18.832	0.000
**Means**				
EXT	0.000	0.000	999.000	999.000
INT	0.000	0.000	999.000	999.000
THD	0.000	0.000	999.000	999.000
**Intercepts**				
SMK18	0.414	0.014	29.282	0.000
CD18	0.931	0.018	51.090	0.000
ADHD18	1.350	0.023	57.504	0.000
PSY18	0.144	0.011	12.741	0.000
ALC18	0.670	0.015	43.283	0.000
MAR18	0.244	0.012	20.944	0.000
GAD18	0.527	0.014	36.592	0.000
MDE18	0.609	0.015	39.369	0.000
EAT18	0.513	0.013	39.864	0.000
PTSD18	0.411	0.012	33.053	0.000
PRODA18	0.356	0.012	29.064	0.000
PRODB18	0.323	0.012	27.106	0.000
**Variances**				
EXT	1.000	0.000	999.000	999.000
INT	1.000	0.000	999.000	999.000
THD	1.000	0.000	999.000	999.000
**Residual Variances**				
SMK18	0.783	0.028	27.504	0.000
CD18	0.489	0.038	12.831	0.000
ADHD18	0.646	0.028	23.501	0.000
PSY18	0.812	0.042	19.330	0.000
ALC18	0.768	0.029	26.623	0.000
MAR18	0.739	0.035	21.374	0.000
GAD18	0.663	0.029	22.514	0.000
MDE18	0.460	0.036	12.665	0.000
EAT18	0.771	0.028	27.830	0.000
PTSD18	0.768	0.032	23.877	0.000

	Estimate	S.E.	Est./S.E.	Two-Tailed P-Value
PRODA18	0.454	0.048	9.410	0.000
PRODB18	0.526	0.045	11.826	0.000

STD Standardization

	Estimate	S.E.	Est./S.E.	Two-Tailed P-Value
EXT BY				
ADHD18	2.550	0.113	22.616	0.000
ALC18	0.809	0.063	12.753	0.000
MAR18	0.703	0.080	8.741	0.000
SMK18	0.755	0.061	12.423	0.000
CD18	1.631	0.080	20.366	0.000
INT BY				
GAD18	1.249	0.065	19.105	0.000
MDE18	2.184	0.089	24.581	0.000
EAT18	0.422	0.032	13.052	0.000
PTSD18	1.597	0.145	10.982	0.000
THD BY				
PSY18	0.132	0.026	5.093	0.000
PRODA18	0.567	0.042	13.586	0.000
PRODB18	0.526	0.040	13.087	0.000
EXT WITH				
INT	0.539	0.038	14.172	0.000
THD	0.483	0.041	11.698	0.000
INT WITH				
THD	0.689	0.037	18.832	0.000
Means				
EXT	0.000	0.000	999.000	999.000
INT	0.000	0.000	999.000	999.000
THD	0.000	0.000	999.000	999.000
Intercepts				
SMK18	0.672	0.044	15.379	0.000
CD18	2.124	0.061	34.833	0.000
ADHD18	5.789	0.105	54.980	0.000
PSY18	0.044	0.007	6.313	0.000
ALC18	1.125	0.042	26.782	0.000
MAR18	0.335	0.034	9.757	0.000
GAD18	1.134	0.052	22.005	0.000
MDE18	1.809	0.072	25.033	0.000
EAT18	0.453	0.022	20.715	0.000
PTSD18	1.365	0.082	16.737	0.000
PRODA18	0.273	0.018	15.101	0.000
PRODB18	0.247	0.018	13.690	0.000
Variances				
EXT	1.000	0.000	999.000	999.000
INT	1.000	0.000	999.000	999.000
THD	1.000	0.000	999.000	999.000
Residual Variances				
SMK18	2.059	0.158	13.028	0.000
CD18	2.548	0.197	12.947	0.000
ADHD18	11.894	0.533	22.296	0.000
PSY18	0.076	0.018	4.186	0.000
ALC18	2.163	0.125	17.330	0.000
MAR18	1.397	0.145	9.635	0.000
GAD18	3.067	0.162	18.938	0.000
MDE18	4.067	0.322	12.634	0.000
EAT18	0.601	0.043	13.917	0.000
PTSD18	8.462	0.532	15.895	0.000
PRODA18	0.267	0.030	8.826	0.000
PRODB18	0.307	0.033	9.432	0.000

R-SQUARE

Observed Variable	Estimate	S.E.	Est./S.E.	Two-Tailed P-Value
SMK18	0.217	0.028	7.608	0.000
CD18	0.511	0.038	13.395	0.000
ADHD18	0.354	0.028	12.852	0.000
PSY18	0.188	0.042	4.464	0.000
ALC18	0.232	0.029	8.057	0.000
MAR18	0.261	0.035	7.552	0.000
GAD18	0.337	0.029	11.452	0.000
MDE18	0.540	0.036	14.852	0.000
EAT18	0.229	0.028	8.260	0.000
PTSD18	0.232	0.032	7.197	0.000
PRODA18	0.546	0.048	11.299	0.000
PRODB18	0.474	0.045	10.644	0.000

SUMMARY OF FACTOR SCORES

FACTOR SCORE INFORMATION (COMPLETE-DATA PATTERN)
FACTOR DETERMINACIES

EXT	0.860
INT	0.865
THD	0.863

FACTOR SCORE INFORMATION (PATTERN 2)
FACTOR DETERMINACIES

EXT	0.858
INT	0.851
THD	0.672

FACTOR SCORE INFORMATION (PATTERN 3)
FACTOR DETERMINACIES

EXT	0.860
INT	0.853
THD	0.862

FACTOR SCORE INFORMATION (PATTERN 4)
FACTOR DETERMINACIES

EXT	0.860
INT	0.853
THD	0.862

FACTOR SCORE INFORMATION (PATTERN 5)
FACTOR DETERMINACIES

EXT	0.859
INT	0.802
THD	0.858

FACTOR SCORE INFORMATION (PATTERN 6)
FACTOR DETERMINACIES

EXT	0.835
INT	0.864
THD	0.863

FACTOR SCORE INFORMATION (PATTERN 7)
FACTOR DETERMINACIES

EXT	0.802
INT	0.863
THD	0.863

FACTOR SCORE INFORMATION (PATTERN 8)

```
          FACTOR DETERMINACIES

          EXT          0.775
          INT          0.863
          THD          0.862

     FACTOR SCORE INFORMATION (PATTERN 9)
          FACTOR DETERMINACIES

          EXT          0.796
          INT          0.845
          THD          0.604

     FACTOR SCORE INFORMATION (PATTERN 10)
          FACTOR DETERMINACIES

          EXT          0.688
          INT          0.764
          THD          0.853

     FACTOR SCORE INFORMATION (PATTERN 11)
          FACTOR DETERMINACIES

          EXT          0.736
          INT          0.842
          THD          0.598

     FACTOR SCORE INFORMATION (PATTERN 12)
          FACTOR DETERMINACIES

          EXT          0.849
          INT          0.865
          THD          0.863

     FACTOR SCORE INFORMATION (PATTERN 13)
          FACTOR DETERMINACIES

          EXT          0.833
          INT          0.864
          THD          0.863

     Beginning Time:  10:12:52
        Ending Time:  10:12:53
       Elapsed Time:  00:00:01
```


```
MUTHEN & MUTHEN
3463 Stoner Ave.
Los Angeles, CA  90066

Tel: (310) 391-9971
Fax: (310) 391-8971
Web: www.StatModel.com
Support: Support@StatModel.com
```



```
Copyright (c) 1998-2021 Muthen & Muthen
```

```
Mplus VERSION 8.7
MUTHEN & MUTHEN
10/27/2022  10:02 AM

INPUT INSTRUCTIONS

  TITLE:  E-Risk P Compare (One-Factor);

  DATA:   FILE IS PCompare_Oct2020.dat;

  VARIABLE:
          NAMES ARE
              familyid atwinid rorder sex zygosity iqe5 seswq35 lowsc harm
              polyv512 aces512 famhist smk18 cd18 adhd18 psy18
              polyv18 CRP18 IL6_18 suPAR iq18 fsiq18
              alc18 mar18 gad18 mde18 eat18 ptsd18 prod18 prodA18 prodB18;

          MISSING
              ALL (999999);

          CLUSTER = familyid;

          USEVARIABLES ARE
              smk18 cd18 adhd18 psy18 alc18 mar18 gad18 mde18 eat18 PTSD18
              prodA18 prodB18;

          IDVARIABLE IS
              atwinid;

  ANALYSIS:
      TYPE      = COMPLEX;
      ESTIMATOR = MLR;
      MODEL     = NOCOVARIANCES;

  MODEL:

      P BY adhd18* alc18 mar18 smk18 cd18 gad18 mde18 eat18 PTSD18 psy18
           prodA18 prodB18;

      [P@0];
       p@1;

  OUTPUT: SAMPSTAT STANDARDIZED FSDETERMINACY PATTERNS;

  !SAVEDATA:
  !    FILE     = OneFactor.dat;
  !    SAVE     = FSCORES;
  !    MISSFLAG = 9999;

*** WARNING
  Data set contains cases with missing on all variables.
  These cases were not included in the analysis.
  Number of cases with missing on all variables:  166
   1 WARNING(S) FOUND IN THE INPUT INSTRUCTIONS

E-Risk P Compare (One-Factor);

SUMMARY OF ANALYSIS

Number of groups                                                 1
Number of observations                                        2066

Number of dependent variables                                   12
Number of independent variables                                  0
Number of continuous latent variables                            1

Observed dependent variables

  Continuous
   SMK18       CD18        ADHD18      PSY18       ALC18       MAR18
```

```
   GAD18       MDE18       EAT18       PTSD18      PRODA18     PRODB18

Continuous latent variables
   P

Variables with special functions

  Cluster variable      FAMILYID
  ID variable           ATWINID

Estimator                                                      MLR
Information matrix                                        OBSERVED
Maximum number of iterations                                  1000
Convergence criterion                                    0.500D-04
Maximum number of steepest descent iterations                   20
Maximum number of iterations for H1                           2000
Convergence criterion for H1                             0.100D-03

Input data file(s)
  PCompare_Oct2020.dat

Input data format  FREE


SUMMARY OF DATA

     Number of missing data patterns            13
     Number of clusters                       1044

SUMMARY OF MISSING DATA PATTERNS

     MISSING DATA PATTERNS (x = not missing)

           1  2  3  4  5  6  7  8  9 10 11 12 13
 SMK18     x  x  x  x  x  x  x  x  x  x  x
 CD18      x  x  x  x  x  x                 x  x
 ADHD18    x  x  x  x  x     x  x  x        x  x
 PSY18     x  x  x  x  x  x  x  x     x     x  x
 ALC18     x  x  x  x  x  x  x     x     x  x
 MAR18     x  x  x  x  x  x  x  x  x  x  x  x  x
 GAD18     x  x  x  x  x  x  x  x  x  x  x  x  x
 MDE18     x  x  x  x     x  x  x  x     x  x  x
 EAT18     x  x  x     x  x  x  x  x     x  x  x
 PTSD18    x  x     x  x  x  x  x  x  x  x  x  x
 PRODA18   x     x  x  x  x  x  x     x     x  x
 PRODB18   x     x  x  x  x  x  x     x     x  x

     MISSING DATA PATTERN FREQUENCIES

    Pattern   Frequency     Pattern   Frequency     Pattern   Frequency
          1        2040           6           3          11           1
          2           1           7           8          12           3
          3           2           8           1          13           1
          4           1           9           2
          5           2          10           1

COVARIANCE COVERAGE OF DATA

Minimum covariance coverage value   0.100

     PROPORTION OF DATA PRESENT


           Covariance Coverage
              SMK18         CD18          ADHD18        PSY18         ALC18
              ________      ________      ________      ________      ________
 SMK18          0.998
 CD18           0.992         0.994
 ADHD18         0.996         0.992         0.998
 PSY18          0.997         0.994         0.997         0.999
```

ALC18	0.997	0.993	0.997	0.997	0.999
MAR18	0.998	0.994	0.998	0.999	0.999
GAD18	0.998	0.994	0.998	0.999	0.999
MDE18	0.997	0.993	0.997	0.997	0.998
EAT18	0.997	0.993	0.997	0.998	0.998
PTSD18	0.997	0.993	0.997	0.998	0.998
PRODA18	0.996	0.993	0.996	0.998	0.997
PRODB18	0.996	0.993	0.996	0.998	0.997

Covariance Coverage

	MAR18	GAD18	MDE18	EAT18	PTSD18
MAR18	1.000				
GAD18	1.000	1.000			
MDE18	0.999	0.999	0.999		
EAT18	0.999	0.999	0.998	0.999	
PTSD18	0.999	0.999	0.998	0.998	0.999
PRODA18	0.998	0.998	0.997	0.997	0.997
PRODB18	0.998	0.998	0.997	0.997	0.997

Covariance Coverage

	PRODA18	PRODB18
PRODA18	0.998	
PRODB18	0.998	0.998

SAMPLE STATISTICS

ESTIMATED SAMPLE STATISTICS

Means

SMK18	CD18	ADHD18	PSY18	ALC18
0.672	2.123	5.788	0.044	1.125

Means

MAR18	GAD18	MDE18	EAT18	PTSD18
0.335	1.134	1.809	0.453	1.365

Means

PRODA18	PRODB18
0.273	0.247

Covariances

	SMK18	CD18	ADHD18	PSY18	ALC18
SMK18	2.630				
CD18	1.183	5.205			
ADHD18	1.560	4.202	18.393		
PSY18	0.044	0.076	0.167	0.094	
ALC18	0.522	1.417	2.176	0.022	2.818
MAR18	0.839	1.260	1.265	0.048	0.442
GAD18	0.292	0.672	2.373	0.109	0.471
MDE18	0.933	1.595	4.348	0.153	1.282
EAT18	0.212	0.341	1.041	0.036	0.299
PTSD18	0.938	1.135	2.751	0.158	0.660
PRODA18	0.181	0.372	0.881	0.081	0.134
PRODB18	0.150	0.437	0.892	0.069	0.217

Covariances

	MAR18	GAD18	MDE18	EAT18	PTSD18
MAR18	1.891				
GAD18	0.241	4.627			
MDE18	0.689	2.941	8.835		
EAT18	0.107	0.505	0.917	0.779	

PTSD18	0.366	1.711	3.372	0.637	11.012
PRODA18	0.201	0.488	0.772	0.177	0.882
PRODB18	0.233	0.489	0.761	0.141	0.690

Covariances

	PRODA18	PRODB18
PRODA18	0.589	
PRODB18	0.293	0.584

Correlations

	SMK18	CD18	ADHD18	PSY18	ALC18
SMK18	1.000				
CD18	0.320	1.000			
ADHD18	0.224	0.429	1.000		
PSY18	0.089	0.109	0.127	1.000	
ALC18	0.192	0.370	0.302	0.044	1.000
MAR18	0.376	0.402	0.215	0.114	0.192
GAD18	0.084	0.137	0.257	0.166	0.131
MDE18	0.194	0.235	0.341	0.168	0.257
EAT18	0.148	0.169	0.275	0.135	0.202
PTSD18	0.174	0.150	0.193	0.156	0.118
PRODA18	0.145	0.212	0.268	0.346	0.104
PRODB18	0.121	0.251	0.272	0.294	0.169

Correlations

	MAR18	GAD18	MDE18	EAT18	PTSD18
MAR18	1.000				
GAD18	0.081	1.000			
MDE18	0.169	0.460	1.000		
EAT18	0.088	0.266	0.350	1.000	
PTSD18	0.080	0.240	0.342	0.218	1.000
PRODA18	0.191	0.296	0.339	0.262	0.346
PRODB18	0.222	0.298	0.335	0.210	0.272

Correlations

	PRODA18	PRODB18
PRODA18	1.000	
PRODB18	0.500	1.000

MAXIMUM LOG-LIKELIHOOD VALUE FOR THE UNRESTRICTED (H1) MODEL IS -42633.558

UNIVARIATE SAMPLE STATISTICS

UNIVARIATE HIGHER-ORDER MOMENT DESCRIPTIVE STATISTICS

Variable/ Sample Size	Mean/ Variance	Skewness/ Kurtosis	Minimum/ Maximum	% with Min/Max	Percentiles 20%/60%	40%/80%	Median
SMK18	0.670	2.585	0.000	80.94%	0.000	0.000	0.000
2062.000	2.625	6.106	10.000	0.05%	0.000	0.000	
CD18	2.121	1.262	0.000	30.54%	0.000	1.000	1.000
2053.000	5.209	1.205	11.000	0.24%	2.000	4.000	
ADHD18	5.787	0.510	0.000	10.92%	2.000	4.000	5.000
2061.000	18.399	-0.528	18.000	0.44%	7.000	10.000	
PSY18	0.044	9.848	0.000	97.14%	0.000	0.000	0.000
2063.000	0.094	125.741	6.000	0.05%	0.000	0.000	
ALC18	1.125	1.949	0.000	53.76%	0.000	0.000	0.000
2063.000	2.818	4.436	11.000	0.10%	1.000	2.000	
MAR18	0.335	4.751	0.000	92.40%	0.000	0.000	0.000
2066.000	1.891	23.548	10.000	0.44%	0.000	0.000	
GAD18	1.134	1.593	0.000	75.46%	0.000	0.000	0.000
2066.000	4.627	0.931	7.000	3.05%	0.000	3.000	
MDE18	1.806	1.273	0.000	68.93%	0.000	0.000	0.000
2063.000	8.828	-0.017	9.000	3.34%	0.000	5.000	
EAT18	0.453	2.416	0.000	71.71%	0.000	0.000	0.000

```
         2064.000       0.779       6.256       5.000    0.44%       0.000      1.000
     PTSD18             1.365       2.642       0.000   79.41%       0.000      0.000      0.000
         2064.000      11.014       6.296      17.000    0.24%       0.000      1.000
     PRODA18            0.273       3.450       0.000   85.55%       0.000      0.000      0.000
         2062.000       0.588      14.002       6.000    0.19%       0.000      0.000
     PRODB18            0.247       3.734       0.000   87.83%       0.000      0.000      0.000
         2062.000       0.584      15.720       6.000    0.19%       0.000      0.000

THE MODEL ESTIMATION TERMINATED NORMALLY

MODEL FIT INFORMATION

Number of Free Parameters                       36

Loglikelihood

          H0 Value                      -43271.522
          H0 Scaling Correction Factor      4.5760
            for MLR
          H1 Value                      -42633.558
          H1 Scaling Correction Factor      3.0107
            for MLR

Information Criteria

          Akaike (AIC)                   86615.044
          Bayesian (BIC)                 86817.845
          Sample-Size Adjusted BIC       86703.470
            (n* = (n + 2) / 24)

Chi-Square Test of Model Fit

          Value                            648.605*
          Degrees of Freedom                    54
          P-Value                           0.0000
          Scaling Correction Factor         1.9672
            for MLR

*   The chi-square value for MLM, MLMV, MLR, ULSMV, WLSM and WLSMV cannot be used
    for chi-square difference testing in the regular way.  MLM, MLR and WLSM
    chi-square difference testing is described on the Mplus website.  MLMV, WLSMV,
    and ULSMV difference testing is done using the DIFFTEST option.

RMSEA (Root Mean Square Error Of Approximation)

          Estimate                           0.073
          90 Percent C.I.                    0.068  0.078
          Probability RMSEA <= .05           0.000

CFI/TLI

          CFI                                0.747
          TLI                                0.691

Chi-Square Test of Model Fit for the Baseline Model

          Value                           2418.883
          Degrees of Freedom                    66
          P-Value                           0.0000

SRMR (Standardized Root Mean Square Residual)

          Value                              0.070
```

MODEL RESULTS

	Estimate	S.E.	Est./S.E.	Two-Tailed P-Value
**P BY**				
ADHD18	2.368	0.111	21.344	0.000
ALC18	0.644	0.069	9.369	0.000
MAR18	0.516	0.076	6.773	0.000
SMK18	0.576	0.062	9.306	0.000
CD18	1.135	0.090	12.593	0.000
GAD18	1.065	0.071	15.068	0.000
MDE18	1.868	0.083	22.384	0.000
EAT18	0.398	0.031	12.729	0.000
PTSD18	1.503	0.143	10.483	0.000
PSY18	0.105	0.021	4.897	0.000
PRODA18	0.456	0.038	12.144	0.000
PRODB18	0.446	0.037	12.067	0.000
**Means**				
P	0.000	0.000	999.000	999.000
**Intercepts**				
SMK18	0.671	0.044	15.379	0.000
CD18	2.125	0.061	34.829	0.000
ADHD18	5.788	0.105	54.972	0.000
PSY18	0.044	0.007	6.312	0.000
ALC18	1.125	0.042	26.779	0.000
MAR18	0.335	0.034	9.757	0.000
GAD18	1.134	0.052	22.005	0.000
MDE18	1.809	0.072	25.026	0.000
EAT18	0.453	0.022	20.711	0.000
PTSD18	1.365	0.082	16.740	0.000
PRODA18	0.273	0.018	15.101	0.000
PRODB18	0.247	0.018	13.689	0.000
**Variances**				
P	1.000	0.000	999.000	999.000
**Residual Variances**				
SMK18	2.295	0.169	13.587	0.000
CD18	3.925	0.214	18.383	0.000
ADHD18	12.792	0.542	23.596	0.000
PSY18	0.083	0.020	4.184	0.000
ALC18	2.404	0.136	17.655	0.000
MAR18	1.625	0.185	8.770	0.000
GAD18	3.492	0.174	20.074	0.000
MDE18	5.347	0.277	19.296	0.000
EAT18	0.620	0.045	13.913	0.000
PTSD18	8.752	0.551	15.893	0.000
PRODA18	0.381	0.032	11.900	0.000
PRODB18	0.385	0.034	11.190	0.000

QUALITY OF NUMERICAL RESULTS

Condition Number for the Information Matrix 0.826E-03
(ratio of smallest to largest eigenvalue)

STANDARDIZED MODEL RESULTS

STDYX Standardization

	Estimate	S.E.	Est./S.E.	Two-Tailed P-Value
**P BY**				
ADHD18	0.552	0.023	23.516	0.000
ALC18	0.383	0.035	10.877	0.000
MAR18	0.375	0.041	9.044	0.000
SMK18	0.355	0.033	10.632	0.000
CD18	0.497	0.035	14.283	0.000
GAD18	0.495	0.029	16.941	0.000

```
    MDE18              0.628      0.023     26.791      0.000
    EAT18              0.451      0.029     15.726      0.000
    PTSD18             0.453      0.034     13.447      0.000
    PSY18              0.343      0.037      9.182      0.000
    PRODA18            0.594      0.031     19.244      0.000
    PRODB18            0.583      0.030     19.228      0.000

 Means
    P                  0.000      0.000    999.000    999.000

 Intercepts
    SMK18              0.414      0.014     29.279      0.000
    CD18               0.931      0.018     51.091      0.000
    ADHD18             1.349      0.023     57.491      0.000
    PSY18              0.144      0.011     12.742      0.000
    ALC18              0.670      0.015     43.283      0.000
    MAR18              0.244      0.012     20.944      0.000
    GAD18              0.527      0.014     36.592      0.000
    MDE18              0.609      0.015     39.369      0.000
    EAT18              0.513      0.013     39.849      0.000
    PTSD18             0.411      0.012     33.063      0.000
    PRODA18            0.356      0.012     29.058      0.000
    PRODB18            0.323      0.012     27.099      0.000

 Variances
    P                  1.000      0.000    999.000    999.000

 Residual Variances
    SMK18              0.874      0.024     36.761      0.000
    CD18               0.753      0.035     21.756      0.000
    ADHD18             0.695      0.026     26.833      0.000
    PSY18              0.882      0.026     34.388      0.000
    ALC18              0.853      0.027     31.544      0.000
    MAR18              0.859      0.031     27.599      0.000
    GAD18              0.755      0.029     26.063      0.000
    MDE18              0.605      0.029     20.534      0.000
    EAT18              0.796      0.026     30.754      0.000
    PTSD18             0.795      0.031     26.035      0.000
    PRODA18            0.647      0.037     17.662      0.000
    PRODB18            0.660      0.035     18.642      0.000

STDY Standardization

                                                    Two-Tailed
                    Estimate       S.E.  Est./S.E.    P-Value

 P        BY
    ADHD18             0.552      0.023     23.516      0.000
    ALC18              0.383      0.035     10.877      0.000
    MAR18              0.375      0.041      9.044      0.000
    SMK18              0.355      0.033     10.632      0.000
    CD18               0.497      0.035     14.283      0.000
    GAD18              0.495      0.029     16.941      0.000
    MDE18              0.628      0.023     26.791      0.000
    EAT18              0.451      0.029     15.726      0.000
    PTSD18             0.453      0.034     13.447      0.000
    PSY18              0.343      0.037      9.182      0.000
    PRODA18            0.594      0.031     19.244      0.000
    PRODB18            0.583      0.030     19.228      0.000

 Means
    P                  0.000      0.000    999.000    999.000

 Intercepts
    SMK18              0.414      0.014     29.279      0.000
    CD18               0.931      0.018     51.091      0.000
    ADHD18             1.349      0.023     57.491      0.000
    PSY18              0.144      0.011     12.742      0.000
    ALC18              0.670      0.015     43.283      0.000
    MAR18              0.244      0.012     20.944      0.000
    GAD18              0.527      0.014     36.592      0.000
```

```
    MDE18              0.609      0.015     39.369      0.000
    EAT18              0.513      0.013     39.849      0.000
    PTSD18             0.411      0.012     33.063      0.000
    PRODA18            0.356      0.012     29.058      0.000
    PRODB18            0.323      0.012     27.099      0.000

 Variances
    P                  1.000      0.000    999.000    999.000

 Residual Variances
    SMK18              0.874      0.024     36.761      0.000
    CD18               0.753      0.035     21.756      0.000
    ADHD18             0.695      0.026     26.833      0.000
    PSY18              0.882      0.026     34.388      0.000
    ALC18              0.853      0.027     31.544      0.000
    MAR18              0.859      0.031     27.599      0.000
    GAD18              0.755      0.029     26.063      0.000
    MDE18              0.605      0.029     20.534      0.000
    EAT18              0.796      0.026     30.754      0.000
    PTSD18             0.795      0.031     26.035      0.000
    PRODA18            0.647      0.037     17.662      0.000
    PRODB18            0.660      0.035     18.642      0.000

STD Standardization
                                                    Two-Tailed
                    Estimate       S.E.  Est./S.E.    P-Value

 P        BY
    ADHD18             2.368      0.111     21.344      0.000
    ALC18              0.644      0.069      9.369      0.000
    MAR18              0.516      0.076      6.773      0.000
    SMK18              0.576      0.062      9.306      0.000
    CD18               1.135      0.090     12.593      0.000
    GAD18              1.065      0.071     15.068      0.000
    MDE18              1.868      0.083     22.384      0.000
    EAT18              0.398      0.031     12.729      0.000
    PTSD18             1.503      0.143     10.483      0.000
    PSY18              0.105      0.021      4.897      0.000
    PRODA18            0.456      0.038     12.144      0.000
    PRODB18            0.446      0.037     12.067      0.000

 Means
    P                  0.000      0.000    999.000    999.000

 Intercepts
    SMK18              0.671      0.044     15.379      0.000
    CD18               2.125      0.061     34.829      0.000
    ADHD18             5.788      0.105     54.972      0.000
    PSY18              0.044      0.007      6.312      0.000
    ALC18              1.125      0.042     26.779      0.000
    MAR18              0.335      0.034      9.757      0.000
    GAD18              1.134      0.052     22.005      0.000
    MDE18              1.809      0.072     25.026      0.000
    EAT18              0.453      0.022     20.711      0.000
    PTSD18             1.365      0.082     16.740      0.000
    PRODA18            0.273      0.018     15.101      0.000
    PRODB18            0.247      0.018     13.689      0.000

 Variances
    P                  1.000      0.000    999.000    999.000

 Residual Variances
    SMK18              2.295      0.169     13.587      0.000
    CD18               3.925      0.214     18.383      0.000
    ADHD18            12.792      0.542     23.596      0.000
    PSY18              0.083      0.020      4.184      0.000
    ALC18              2.404      0.136     17.655      0.000
    MAR18              1.625      0.185      8.770      0.000
    GAD18              3.492      0.174     20.074      0.000
    MDE18              5.347      0.277     19.296      0.000
```

EAT18	0.620	0.045	13.913	0.000
PTSD18	8.752	0.551	15.893	0.000
PRODA18	0.381	0.032	11.900	0.000
PRODB18	0.385	0.034	11.190	0.000

R-SQUARE

Observed Variable	Estimate	S.E.	Est./S.E.	Two-Tailed P-Value
SMK18	0.126	0.024	5.316	0.000
CD18	0.247	0.035	7.142	0.000
ADHD18	0.305	0.026	11.758	0.000
PSY18	0.118	0.026	4.591	0.000
ALC18	0.147	0.027	5.438	0.000
MAR18	0.141	0.031	4.522	0.000
GAD18	0.245	0.029	8.471	0.000
MDE18	0.395	0.029	13.396	0.000
EAT18	0.204	0.026	7.863	0.000
PTSD18	0.205	0.031	6.724	0.000
PRODA18	0.353	0.037	9.622	0.000
PRODB18	0.340	0.035	9.614	0.000

SUMMARY OF FACTOR SCORES

FACTOR SCORE INFORMATION (COMPLETE-DATA PATTERN)
FACTOR DETERMINACIES

P 0.893

FACTOR SCORE INFORMATION (PATTERN 2)
FACTOR DETERMINACIES

P 0.861

FACTOR SCORE INFORMATION (PATTERN 3)
FACTOR DETERMINACIES

P 0.887

FACTOR SCORE INFORMATION (PATTERN 4)
FACTOR DETERMINACIES

P 0.887

FACTOR SCORE INFORMATION (PATTERN 5)
FACTOR DETERMINACIES

P 0.875

FACTOR SCORE INFORMATION (PATTERN 6)
FACTOR DETERMINACIES

P 0.882

FACTOR SCORE INFORMATION (PATTERN 7)
FACTOR DETERMINACIES

P 0.885

FACTOR SCORE INFORMATION (PATTERN 8)
FACTOR DETERMINACIES
P 0.880

FACTOR SCORE INFORMATION (PATTERN 9)
FACTOR DETERMINACIES

P 0.841

FACTOR SCORE INFORMATION (PATTERN 10)

```
     FACTOR DETERMINACIES

     P          0.822

FACTOR SCORE INFORMATION (PATTERN 11)
     FACTOR DETERMINACIES

     P          0.815

FACTOR SCORE INFORMATION (PATTERN 12)
     FACTOR DETERMINACIES

     P          0.889

FACTOR SCORE INFORMATION (PATTERN 13)
     FACTOR DETERMINACIES

     P          0.885


Beginning Time:  10:02:25
   Ending Time:  10:02:26
  Elapsed Time:  00:00:01
```

MUTHEN & MUTHEN
3463 Stoner Ave.
Los Angeles, CA 90066

Tel: (310) 391-9971
Fax: (310) 391-8971
Web: www.StatModel.com
Support: Support@StatModel.com

Copyright (c) 1998-2021 Muthen & Muthen

```
Mplus VERSION 8.7
MUTHEN & MUTHEN
10/27/2022  10:06 AM

INPUT INSTRUCTIONS

  TITLE:  E-Risk P Compare (Higher-Order Factor);

  DATA:   FILE IS PCompare_Oct2020.dat;

  VARIABLE:
          NAMES ARE
              familyid atwinid rorder sex zygosity iqe5 seswq35 lowsc harm
              polyv512 aces512 famhist smk18 cd18 adhd18 psy18
              polyv18 CRP18 IL6_18 suPAR iq18 fsiq18
              alc18 mar18 gad18 mde18 eat18 ptsd18 prod18 prodA18 prodB18;

          MISSING
              ALL (999999);

          CLUSTER = familyid;

          USEVARIABLES ARE
              smk18 cd18 adhd18 psy18 alc18 mar18 gad18 mde18 eat18 PTSD18
              prodA18 prodB18;

          IDVARIABLE IS
              atwinid;

  ANALYSIS:
      TYPE      = COMPLEX;
      ESTIMATOR = MLR;
      MODEL     = NOCOVARIANCES;

  MODEL:

      ext BY adhd18* alc18 mar18 smk18 cd18;
      int BY gad18*  mde18 eat18 PTSD18;
      thd BY psy18*  prodA18 prodB18;

      p   BY ext* int thd;

      [ext@0 int@0 thd@0 p@0];
       ext@1 int@1 thd@1 p@1;

  OUTPUT: SAMPSTAT STANDARDIZED FSDETERMINACY PATTERNS;

  !SAVEDATA:
  !    FILE     = HigherOrder.dat;
  !    SAVE     = FSCORES;
  !    MISSFLAG = 9999;

*** WARNING
  Data set contains cases with missing on all variables.
  These cases were not included in the analysis.
  Number of cases with missing on all variables:  166
   1 WARNING(S) FOUND IN THE INPUT INSTRUCTIONS

E-Risk P Compare (Higher-Order Factor);

SUMMARY OF ANALYSIS

Number of groups                                                 1
Number of observations                                        2066

Number of dependent variables                                   12
Number of independent variables                                  0
Number of continuous latent variables                            4

Observed dependent variables
```

```
  Continuous
   SMK18       CD18        ADHD18      PSY18       ALC18       MAR18
   GAD18       MDE18       EAT18       PTSD18      PRODA18     PRODB18

Continuous latent variables
   EXT         INT         THD         P

Variables with special functions

  Cluster variable      FAMILYID
  ID variable           ATWINID

Estimator                                                      MLR
Information matrix                                        OBSERVED
Maximum number of iterations                                  1000
Convergence criterion                                    0.500D-04
Maximum number of steepest descent iterations                   20
Maximum number of iterations for H1                           2000
Convergence criterion for H1                             0.100D-03

Input data file(s)
  PCompare_Oct2020.dat

Input data format  FREE

SUMMARY OF DATA

     Number of missing data patterns           13
     Number of clusters                      1044

SUMMARY OF MISSING DATA PATTERNS

     MISSING DATA PATTERNS (x = not missing)

             1  2  3  4  5  6  7  8  9 10 11 12 13
 SMK18       x  x  x  x  x  x  x  x  x  x  x
 CD18        x  x  x  x  x  x                 x  x
 ADHD18      x  x  x  x  x     x  x  x        x  x
 PSY18       x  x  x  x  x  x  x  x     x     x  x
 ALC18       x  x  x  x  x  x  x     x     x  x
 MAR18       x  x  x  x  x  x  x  x  x  x  x  x  x
 GAD18       x  x  x  x  x  x  x  x  x  x  x  x  x
 MDE18       x  x  x  x     x  x  x  x     x  x  x
 EAT18       x  x  x     x  x  x  x  x     x  x  x
 PTSD18      x  x     x  x  x  x  x  x  x  x  x  x
 PRODA18     x     x  x  x  x  x  x     x     x  x
 PRODB18     x     x  x  x  x  x  x     x     x  x

     MISSING DATA PATTERN FREQUENCIES

    Pattern   Frequency     Pattern   Frequency     Pattern   Frequency
          1        2040           6           3          11           1
          2           1           7           8          12           3
          3           2           8           1          13           1
          4           1           9           2
          5           2          10           1

COVARIANCE COVERAGE OF DATA

Minimum covariance coverage value   0.100

     PROPORTION OF DATA PRESENT

           Covariance Coverage
              SMK18         CD18          ADHD18        PSY18         ALC18
              ________      ________      ________      ________      ________
 SMK18          0.998
 CD18           0.992         0.994
 ADHD18         0.996         0.992         0.998
```

PSY18	0.997	0.994	0.997	0.999	
ALC18	0.997	0.993	0.997	0.997	0.999
MAR18	0.998	0.994	0.998	0.999	0.999
GAD18	0.998	0.994	0.998	0.999	0.999
MDE18	0.997	0.993	0.997	0.997	0.998
EAT18	0.997	0.993	0.997	0.998	0.998
PTSD18	0.997	0.993	0.997	0.998	0.998
PRODA18	0.996	0.993	0.996	0.998	0.997
PRODB18	0.996	0.993	0.996	0.998	0.997

Covariance Coverage

	MAR18	GAD18	MDE18	EAT18	PTSD18
MAR18	1.000				
GAD18	1.000	1.000			
MDE18	0.999	0.999	0.999		
EAT18	0.999	0.999	0.998	0.999	
PTSD18	0.999	0.999	0.998	0.998	0.999
PRODA18	0.998	0.998	0.997	0.997	0.997
PRODB18	0.998	0.998	0.997	0.997	0.997

Covariance Coverage

	PRODA18	PRODB18
PRODA18	0.998	
PRODB18	0.998	0.998

SAMPLE STATISTICS

ESTIMATED SAMPLE STATISTICS

Means

SMK18	CD18	ADHD18	PSY18	ALC18
0.672	2.123	5.788	0.044	1.125

Means

MAR18	GAD18	MDE18	EAT18	PTSD18
0.335	1.134	1.809	0.453	1.365

Means

PRODA18	PRODB18
0.273	0.247

Covariances

	SMK18	CD18	ADHD18	PSY18	ALC18
SMK18	2.630				
CD18	1.183	5.205			
ADHD18	1.560	4.202	18.393		
PSY18	0.044	0.076	0.167	0.094	
ALC18	0.522	1.417	2.176	0.022	2.818
MAR18	0.839	1.260	1.265	0.048	0.442
GAD18	0.292	0.672	2.373	0.109	0.471
MDE18	0.933	1.595	4.348	0.153	1.282
EAT18	0.212	0.341	1.041	0.036	0.299
PTSD18	0.938	1.135	2.751	0.158	0.660
PRODA18	0.181	0.372	0.881	0.081	0.134
PRODB18	0.150	0.437	0.892	0.069	0.217

Covariances

	MAR18	GAD18	MDE18	EAT18	PTSD18
MAR18	1.891				
GAD18	0.241	4.627			
MDE18	0.689	2.941	8.835		
EAT18	0.107	0.505	0.917	0.779	
PTSD18	0.366	1.711	3.372	0.637	11.012

PRODA18	0.201	0.488	0.772	0.177	0.882
PRODB18	0.233	0.489	0.761	0.141	0.690

Covariances

	PRODA18	PRODB18
PRODA18	0.589	
PRODB18	0.293	0.584

Correlations

	SMK18	CD18	ADHD18	PSY18	ALC18
SMK18	1.000				
CD18	0.320	1.000			
ADHD18	0.224	0.429	1.000		
PSY18	0.089	0.109	0.127	1.000	
ALC18	0.192	0.370	0.302	0.044	1.000
MAR18	0.376	0.402	0.215	0.114	0.192
GAD18	0.084	0.137	0.257	0.166	0.131
MDE18	0.194	0.235	0.341	0.168	0.257
EAT18	0.148	0.169	0.275	0.135	0.202
PTSD18	0.174	0.150	0.193	0.156	0.118
PRODA18	0.145	0.212	0.268	0.346	0.104
PRODB18	0.121	0.251	0.272	0.294	0.169

Correlations

	MAR18	GAD18	MDE18	EAT18	PTSD18
MAR18	1.000				
GAD18	0.081	1.000			
MDE18	0.169	0.460	1.000		
EAT18	0.088	0.266	0.350	1.000	
PTSD18	0.080	0.240	0.342	0.218	1.000
PRODA18	0.191	0.296	0.339	0.262	0.346
PRODB18	0.222	0.298	0.335	0.210	0.272

Correlations

	PRODA18	PRODB18
PRODA18	1.000	
PRODB18	0.500	1.000

MAXIMUM LOG-LIKELIHOOD VALUE FOR THE UNRESTRICTED (H1) MODEL IS -42633.558

UNIVARIATE SAMPLE STATISTICS

UNIVARIATE HIGHER-ORDER MOMENT DESCRIPTIVE STATISTICS

Variable/ Sample Size	Mean/ Variance	Skewness/ Kurtosis	Minimum/ Maximum	% with Min/Max	Percentiles 20%/60%	40%/80%	Median
SMK18	0.670	2.585	0.000	80.94%	0.000	0.000	0.000
2062.000	2.625	6.106	10.000	0.05%	0.000	0.000	
CD18	2.121	1.262	0.000	30.54%	0.000	1.000	1.000
2053.000	5.209	1.205	11.000	0.24%	2.000	4.000	
ADHD18	5.787	0.510	0.000	10.92%	2.000	4.000	5.000
2061.000	18.399	-0.528	18.000	0.44%	7.000	10.000	
PSY18	0.044	9.848	0.000	97.14%	0.000	0.000	0.000
2063.000	0.094	125.741	6.000	0.05%	0.000	0.000	
ALC18	1.125	1.949	0.000	53.76%	0.000	0.000	0.000
2063.000	2.818	4.436	11.000	0.10%	1.000	2.000	
MAR18	0.335	4.751	0.000	92.40%	0.000	0.000	0.000
2066.000	1.891	23.548	10.000	0.44%	0.000	0.000	
GAD18	1.134	1.593	0.000	75.46%	0.000	0.000	0.000
2066.000	4.627	0.931	7.000	3.05%	0.000	3.000	
MDE18	1.806	1.273	0.000	68.93%	0.000	0.000	0.000
2063.000	8.828	-0.017	9.000	3.34%	0.000	5.000	
EAT18	0.453	2.416	0.000	71.71%	0.000	0.000	0.000
2064.000	0.779	6.256	5.000	0.44%	0.000	1.000	
PTSD18	1.365	2.642	0.000	79.41%	0.000	0.000	0.000

```
          2064.000      11.014       6.296      17.000    0.24%       0.000      1.000
     PRODA18                 0.273       3.450       0.000   85.55%       0.000      0.000      0.000
          2062.000       0.588      14.002       6.000    0.19%       0.000      0.000
     PRODB18                 0.247       3.734       0.000   87.83%       0.000      0.000      0.000
          2062.000       0.584      15.720       6.000    0.19%       0.000      0.000
```

```
THE MODEL ESTIMATION TERMINATED NORMALLY

MODEL FIT INFORMATION

Number of Free Parameters                       39

Loglikelihood

          H0 Value                      -42854.585
          H0 Scaling Correction Factor      4.4590
            for MLR
          H1 Value                      -42633.558
          H1 Scaling Correction Factor      3.0107
            for MLR

Information Criteria

          Akaike (AIC)                   85787.170
          Bayesian (BIC)                 86006.871
          Sample-Size Adjusted BIC       85882.965
            (n* = (n + 2) / 24)

Chi-Square Test of Model Fit

          Value                            232.263*
          Degrees of Freedom                    51
          P-Value                           0.0000
          Scaling Correction Factor         1.9032
            for MLR

*   The chi-square value for MLM, MLMV, MLR, ULSMV, WLSM and WLSMV cannot be used
    for chi-square difference testing in the regular way.  MLM, MLR and WLSM
    chi-square difference testing is described on the Mplus website.  MLMV, WLSMV,
    and ULSMV difference testing is done using the DIFFTEST option.

RMSEA (Root Mean Square Error Of Approximation)

          Estimate                           0.041
          90 Percent C.I.                    0.036  0.047
          Probability RMSEA <= .05           0.995

CFI/TLI

          CFI                                0.923
          TLI                                0.900

Chi-Square Test of Model Fit for the Baseline Model

          Value                           2418.883
          Degrees of Freedom                    66
          P-Value                           0.0000

SRMR (Standardized Root Mean Square Residual)

          Value                              0.042

MODEL RESULTS
                                                    Two-Tailed
                    Estimate       S.E.  Est./S.E.    P-Value

 EXT      BY
    ADHD18             2.013      0.103     19.498      0.000
    ALC18              0.639      0.052     12.390      0.000
    MAR18              0.554      0.068      8.175      0.000
```

```
   SMK18              0.596      0.055     10.866      0.000
   CD18               1.287      0.091     14.110      0.000

 INT      BY
   GAD18              0.599      0.102      5.859      0.000
   MDE18              1.048      0.187      5.597      0.000
   EAT18              0.203      0.034      5.971      0.000
   PTSD18             0.766      0.121      6.329      0.000

 THD      BY
   PSY18              0.082      0.018      4.537      0.000
   PRODA18            0.351      0.041      8.543      0.000
   PRODB18            0.325      0.031     10.653      0.000

 P        BY
   EXT                0.778      0.086      9.088      0.000
   INT                1.829      0.385      4.751      0.000
   THD                1.270      0.164      7.761      0.000

 Means
   P                  0.000      0.000    999.000    999.000

 Intercepts
   SMK18              0.672      0.044     15.379      0.000
   CD18               2.124      0.061     34.833      0.000
   ADHD18             5.789      0.105     54.980      0.000
   PSY18              0.044      0.007      6.313      0.000
   ALC18              1.125      0.042     26.782      0.000
   MAR18              0.335      0.034      9.757      0.000
   GAD18              1.134      0.052     22.005      0.000
   MDE18              1.809      0.072     25.033      0.000
   EAT18              0.453      0.022     20.715      0.000
   PTSD18             1.365      0.082     16.737      0.000
   PRODA18            0.273      0.018     15.101      0.000
   PRODB18            0.247      0.018     13.690      0.000
   EXT                0.000      0.000    999.000    999.000
   INT                0.000      0.000    999.000    999.000
   THD                0.000      0.000    999.000    999.000

 Variances
   P                  1.000      0.000    999.000    999.000

 Residual Variances
   SMK18              2.059      0.158     13.028      0.000
   CD18               2.548      0.197     12.947      0.000
   ADHD18            11.894      0.533     22.296      0.000
   PSY18              0.076      0.018      4.186      0.000
   ALC18              2.163      0.125     17.330      0.000
   MAR18              1.397      0.145      9.635      0.000
   GAD18              3.067      0.162     18.938      0.000
   MDE18              4.067      0.322     12.634      0.000
   EAT18              0.601      0.043     13.917      0.000
   PTSD18             8.462      0.532     15.895      0.000
   PRODA18            0.267      0.030      8.826      0.000
   PRODB18            0.307      0.033      9.432      0.000
   EXT                1.000      0.000    999.000    999.000
   INT                1.000      0.000    999.000    999.000
   THD                1.000      0.000    999.000    999.000

QUALITY OF NUMERICAL RESULTS

     Condition Number for the Information Matrix              0.619E-04
       (ratio of smallest to largest eigenvalue)

STANDARDIZED MODEL RESULTS

STDYX Standardization

                                                    Two-Tailed
                    Estimate       S.E.  Est./S.E.    P-Value
```

```
 EXT      BY
    ADHD18             0.595      0.023     25.704      0.000
    ALC18              0.482      0.030     16.113      0.000
    MAR18              0.511      0.034     15.105      0.000
    SMK18              0.465      0.031     15.216      0.000
    CD18               0.715      0.027     26.791      0.000

 INT      BY
    GAD18              0.581      0.025     22.905      0.000
    MDE18              0.735      0.025     29.704      0.000
    EAT18              0.478      0.029     16.521      0.000
    PTSD18             0.481      0.033     14.395      0.000

 THD      BY
    PSY18              0.433      0.049      8.927      0.000
    PRODA18            0.739      0.033     22.598      0.000
    PRODB18            0.688      0.032     21.288      0.000

 P        BY
    EXT                0.614      0.042     14.592      0.000
    INT                0.877      0.043     20.644      0.000
    THD                0.786      0.039     20.280      0.000

 Means
    P                  0.000      0.000    999.000    999.000

 Intercepts
    SMK18              0.414      0.014     29.282      0.000
    CD18               0.931      0.018     51.090      0.000
    ADHD18             1.350      0.023     57.504      0.000
    PSY18              0.144      0.011     12.741      0.000
    ALC18              0.670      0.015     43.283      0.000
    MAR18              0.244      0.012     20.944      0.000
    GAD18              0.527      0.014     36.592      0.000
    MDE18              0.609      0.015     39.369      0.000
    EAT18              0.513      0.013     39.864      0.000
    PTSD18             0.411      0.012     33.053      0.000
    PRODA18            0.356      0.012     29.064      0.000
    PRODB18            0.323      0.012     27.106      0.000
    EXT                0.000      0.000    999.000    999.000
    INT                0.000      0.000    999.000    999.000
    THD                0.000      0.000    999.000    999.000

 Variances
    P                  1.000      0.000    999.000    999.000

 Residual Variances
    SMK18              0.783      0.028     27.504      0.000
    CD18               0.489      0.038     12.831      0.000
    ADHD18             0.646      0.028     23.501      0.000
    PSY18              0.812      0.042     19.330      0.000
    ALC18              0.768      0.029     26.623      0.000
    MAR18              0.739      0.035     21.374      0.000
    GAD18              0.663      0.029     22.514      0.000
    MDE18              0.460      0.036     12.665      0.000
    EAT18              0.771      0.028     27.830      0.000
    PTSD18             0.768      0.032     23.877      0.000
    PRODA18            0.454      0.048      9.410      0.000
    PRODB18            0.526      0.045     11.826      0.000
    EXT                0.623      0.052     12.047      0.000
    INT                0.230      0.075      3.085      0.002
    THD                0.383      0.061      6.287      0.000

STDY Standardization

                                                    Two-Tailed
                    Estimate       S.E.  Est./S.E.    P-Value

 EXT      BY
    ADHD18             0.595      0.023     25.704      0.000
```

```
    ALC18              0.482      0.030     16.113      0.000
    MAR18              0.511      0.034     15.105      0.000
    SMK18              0.465      0.031     15.216      0.000
    CD18               0.715      0.027     26.791      0.000

 INT      BY
    GAD18              0.581      0.025     22.905      0.000
    MDE18              0.735      0.025     29.704      0.000
    EAT18              0.478      0.029     16.521      0.000
    PTSD18             0.481      0.033     14.395      0.000

 THD      BY
    PSY18              0.433      0.049      8.927      0.000
    PRODA18            0.739      0.033     22.598      0.000
    PRODB18            0.688      0.032     21.288      0.000

 P        BY
    EXT                0.614      0.042     14.592      0.000
    INT                0.877      0.043     20.644      0.000
    THD                0.786      0.039     20.280      0.000

 Means
    P                  0.000      0.000    999.000    999.000

 Intercepts
    SMK18              0.414      0.014     29.282      0.000
    CD18               0.931      0.018     51.090      0.000
    ADHD18             1.350      0.023     57.504      0.000
    PSY18              0.144      0.011     12.741      0.000
    ALC18              0.670      0.015     43.283      0.000
    MAR18              0.244      0.012     20.944      0.000
    GAD18              0.527      0.014     36.592      0.000
    MDE18              0.609      0.015     39.369      0.000
    EAT18              0.513      0.013     39.864      0.000
    PTSD18             0.411      0.012     33.053      0.000
    PRODA18            0.356      0.012     29.064      0.000
    PRODB18            0.323      0.012     27.106      0.000
    EXT                0.000      0.000    999.000    999.000
    INT                0.000      0.000    999.000    999.000
    THD                0.000      0.000    999.000    999.000

 Variances
    P                  1.000      0.000    999.000    999.000

 Residual Variances
    SMK18              0.783      0.028     27.504      0.000
    CD18               0.489      0.038     12.831      0.000
    ADHD18             0.646      0.028     23.501      0.000
    PSY18              0.812      0.042     19.330      0.000
    ALC18              0.768      0.029     26.623      0.000
    MAR18              0.739      0.035     21.374      0.000
    GAD18              0.663      0.029     22.514      0.000
    MDE18              0.460      0.036     12.665      0.000
    EAT18              0.771      0.028     27.830      0.000
    PTSD18             0.768      0.032     23.877      0.000
    PRODA18            0.454      0.048      9.410      0.000
    PRODB18            0.526      0.045     11.826      0.000
    EXT                0.623      0.052     12.047      0.000
    INT                0.230      0.075      3.085      0.002
    THD                0.383      0.061      6.287      0.000

STD Standardization

                                                    Two-Tailed
                    Estimate       S.E.  Est./S.E.    P-Value

 EXT      BY
    ADHD18             2.550      0.113     22.616      0.000
    ALC18              0.809      0.063     12.753      0.000
    MAR18              0.703      0.080      8.741      0.000
    SMK18              0.755      0.061     12.423      0.000
```

CD18	1.631	0.080	20.366	0.000
**INT BY**				
GAD18	1.249	0.065	19.105	0.000
MDE18	2.184	0.089	24.581	0.000
EAT18	0.422	0.032	13.052	0.000
PTSD18	1.597	0.145	10.982	0.000
**THD BY**				
PSY18	0.132	0.026	5.093	0.000
PRODA18	0.567	0.042	13.586	0.000
PRODB18	0.526	0.040	13.087	0.000
**P BY**				
EXT	0.614	0.042	14.592	0.000
INT	0.877	0.043	20.644	0.000
THD	0.786	0.039	20.280	0.000
**Means**				
P	0.000	0.000	999.000	999.000
**Intercepts**				
SMK18	0.672	0.044	15.379	0.000
CD18	2.124	0.061	34.833	0.000
ADHD18	5.789	0.105	54.980	0.000
PSY18	0.044	0.007	6.313	0.000
ALC18	1.125	0.042	26.782	0.000
MAR18	0.335	0.034	9.757	0.000
GAD18	1.134	0.052	22.005	0.000
MDE18	1.809	0.072	25.033	0.000
EAT18	0.453	0.022	20.715	0.000
PTSD18	1.365	0.082	16.737	0.000
PRODA18	0.273	0.018	15.101	0.000
PRODB18	0.247	0.018	13.690	0.000
EXT	0.000	0.000	999.000	999.000
INT	0.000	0.000	999.000	999.000
THD	0.000	0.000	999.000	999.000
**Variances**				
P	1.000	0.000	999.000	999.000
**Residual Variances**				
SMK18	2.059	0.158	13.028	0.000
CD18	2.548	0.197	12.947	0.000
ADHD18	11.894	0.533	22.296	0.000
PSY18	0.076	0.018	4.186	0.000
ALC18	2.163	0.125	17.330	0.000
MAR18	1.397	0.145	9.635	0.000
GAD18	3.067	0.162	18.938	0.000
MDE18	4.067	0.322	12.634	0.000
EAT18	0.601	0.043	13.917	0.000
PTSD18	8.462	0.532	15.895	0.000
PRODA18	0.267	0.030	8.826	0.000
PRODB18	0.307	0.033	9.432	0.000
EXT	0.623	0.052	12.047	0.000
INT	0.230	0.075	3.085	0.002
THD	0.383	0.061	6.287	0.000

R-SQUARE

Observed Variable	Estimate	S.E.	Est./S.E.	Two-Tailed P-Value
SMK18	0.217	0.028	7.608	0.000
CD18	0.511	0.038	13.395	0.000
ADHD18	0.354	0.028	12.852	0.000
PSY18	0.188	0.042	4.464	0.000
ALC18	0.232	0.029	8.057	0.000
MAR18	0.261	0.035	7.552	0.000
GAD18	0.337	0.029	11.452	0.000

MDE18	0.540	0.036	14.852	0.000
EAT18	0.229	0.028	8.260	0.000
PTSD18	0.232	0.032	7.197	0.000
PRODA18	0.546	0.048	11.299	0.000
PRODB18	0.474	0.045	10.644	0.000

Latent Variable	Estimate	S.E.	Est./S.E.	Two-Tailed P-Value
EXT	0.377	0.052	7.296	0.000
INT	0.770	0.075	10.322	0.000
THD	0.617	0.061	10.140	0.000

SUMMARY OF FACTOR SCORES

FACTOR SCORE INFORMATION (COMPLETE-DATA PATTERN)

FACTOR DETERMINACIES

EXT	0.860
INT	0.865
THD	0.863
P	0.834

FACTOR SCORE INFORMATION (PATTERN 2)

FACTOR DETERMINACIES

EXT	0.858
INT	0.851
THD	0.672
P	0.789

FACTOR SCORE INFORMATION (PATTERN 3)

FACTOR DETERMINACIES

EXT	0.860
INT	0.853
THD	0.862
P	0.828

FACTOR SCORE INFORMATION (PATTERN 4)

FACTOR DETERMINACIES

EXT	0.860
INT	0.853
THD	0.862
P	0.828

FACTOR SCORE INFORMATION (PATTERN 5)

FACTOR DETERMINACIES

EXT	0.859
INT	0.802
THD	0.858
P	0.803

FACTOR SCORE INFORMATION (PATTERN 6)

FACTOR DETERMINACIES

EXT	0.835
INT	0.864
THD	0.863
P	0.832

FACTOR SCORE INFORMATION (PATTERN 7)

FACTOR DETERMINACIES

EXT	0.802
INT	0.863
THD	0.863
P	0.829

```
     FACTOR SCORE INFORMATION (PATTERN 8)
           FACTOR DETERMINACIES

           EXT        0.775
           INT        0.863
           THD        0.862
           P          0.827

     FACTOR SCORE INFORMATION (PATTERN 9)
           FACTOR DETERMINACIES

           EXT        0.796
           INT        0.845
           THD        0.604
           P          0.768

     FACTOR SCORE INFORMATION (PATTERN 10)
           FACTOR DETERMINACIES

           EXT        0.688
           INT        0.764
           THD        0.853
           P          0.770

     FACTOR SCORE INFORMATION (PATTERN 11)
           FACTOR DETERMINACIES

           EXT        0.736
           INT        0.842
           THD        0.598
           P          0.761

     FACTOR SCORE INFORMATION (PATTERN 12)
           FACTOR DETERMINACIES

           EXT        0.849
           INT        0.865
           THD        0.863
           P          0.833

     FACTOR SCORE INFORMATION (PATTERN 13)
           FACTOR DETERMINACIES

           EXT        0.833
           INT        0.864
           THD        0.863
           P          0.832

     Beginning Time:  10:06:00
        Ending Time:  10:06:00
       Elapsed Time:  00:00:00

MUTHEN & MUTHEN
3463 Stoner Ave.
Los Angeles, CA  90066

Tel: (310) 391-9971
Fax: (310) 391-8971
Web: www.StatModel.com
Support: Support@StatModel.com
```

```
Copyright (c) 1998-2021 Muthen & Muthen
Mplus VERSION 8.7
MUTHEN & MUTHEN
10/27/2022   9:57 AM

INPUT INSTRUCTIONS

  TITLE:  E-Risk P Compare (Bifactor - Orthogonal);

  DATA:   FILE IS PCompare_Oct2020.dat;

  VARIABLE:
          NAMES ARE
              familyid atwinid rorder sex zygosity iqe5 seswq35 lowsc harm
              polyv512 aces512 famhist smk18 cd18 adhd18 psy18
              polyv18 CRP18 IL6_18 suPAR iq18 fsiq18
              alc18 mar18 gad18 mde18 eat18 ptsd18 prod18 prodA18 prodB18;

          MISSING
              ALL (999999);

          CLUSTER = familyid;

          USEVARIABLES ARE
              smk18 cd18 adhd18 psy18 alc18 mar18 gad18 mde18 eat18 PTSD18
              prodA18 prodB18;

          IDVARIABLE IS
              atwinid;

  ANALYSIS:
      TYPE      = COMPLEX;
      ESTIMATOR = MLR;
      MODEL     = NOCOVARIANCES;

  MODEL:

      ext BY adhd18* alc18 mar18 smk18 cd18;
      int BY gad18*  mde18 eat18 PTSD18;
      thd BY psy18*  prodA18 prodB18;

      p   BY adhd18* alc18 mar18 smk18 cd18 gad18 mde18 eat18 PTSD18 psy18
             prodA18 prodB18;

      [ext@0 int@0 thd@0 p@0];
       ext@1 int@1 thd@1 p@1;

      ext WITH int@0 thd@0;
      int WITH thd@0;
      P   WITH ext@0 int@0 thd@0;

  OUTPUT: SAMPSTAT STANDARDIZED FSDETERMINACY PATTERNS;

  !SAVEDATA:
  !    FILE     = BifactorOrtho.dat;
  !    SAVE     = FSCORES;
  !    MISSFLAG = 9999;

*** WARNING
  Data set contains cases with missing on all variables.
  These cases were not included in the analysis.
  Number of cases with missing on all variables:  166
   1 WARNING(S) FOUND IN THE INPUT INSTRUCTIONS

E-Risk P Compare (Bifactor - Orthogonal);

SUMMARY OF ANALYSIS

Number of groups                                                 1
Number of observations                                        2066
```

Number of dependent variables	12
Number of independent variables	0
Number of continuous latent variables	4

Observed dependent variables

Continuous

SMK18	CD18	ADHD18	PSY18	ALC18	MAR18
GAD18	MDE18	EAT18	PTSD18	PRODA18	PRODB18

Continuous latent variables

EXT	INT	THD	P

Variables with special functions

Cluster variable	FAMILYID
ID variable	ATWINID

Estimator	MLR
Information matrix	OBSERVED
Maximum number of iterations	1000
Convergence criterion	0.500D-04
Maximum number of steepest descent iterations	20
Maximum number of iterations for H1	2000
Convergence criterion for H1	0.100D-03

Input data file(s)
PCompare_Oct2020.dat

Input data format FREE

SUMMARY OF DATA

Number of missing data patterns	13
Number of clusters	1044

SUMMARY OF MISSING DATA PATTERNS

MISSING DATA PATTERNS (x = not missing)

	1	2	3	4	5	6	7	8	9	10	11	12	13
SMK18	x	x	x	x	x	x	x	x	x	x	x		
CD18	x	x	x	x	x	x						x	x
ADHD18	x	x	x	x	x		x	x	x			x	x
PSY18	x	x	x	x	x	x	x	x		x		x	x
ALC18	x	x	x	x	x	x	x		x		x	x	
MAR18	x	x	x	x	x	x	x	x	x	x	x	x	x
GAD18	x	x	x	x	x	x	x	x	x	x	x	x	x
MDE18	x	x	x	x		x	x	x	x		x	x	x
EAT18	x	x	x		x	x	x	x	x		x	x	x
PTSD18	x	x		x	x	x	x	x	x	x	x	x	x
PRODA18	x		x	x	x	x	x	x		x		x	x
PRODB18	x		x	x	x	x	x	x		x		x	x

MISSING DATA PATTERN FREQUENCIES

Pattern	Frequency	Pattern	Frequency	Pattern	Frequency
1	2040	6	3	11	1
2	1	7	8	12	3
3	2	8	1	13	1
4	1	9	2		
5	2	10	1		

COVARIANCE COVERAGE OF DATA

Minimum covariance coverage value 0.100

PROPORTION OF DATA PRESENT

Covariance Coverage

	SMK18	CD18	ADHD18	PSY18	ALC18
SMK18	0.998				
CD18	0.992	0.994			
ADHD18	0.996	0.992	0.998		
PSY18	0.997	0.994	0.997	0.999	
ALC18	0.997	0.993	0.997	0.997	0.999
MAR18	0.998	0.994	0.998	0.999	0.999
GAD18	0.998	0.994	0.998	0.999	0.999
MDE18	0.997	0.993	0.997	0.997	0.998
EAT18	0.997	0.993	0.997	0.998	0.998
PTSD18	0.997	0.993	0.997	0.998	0.998
PRODA18	0.996	0.993	0.996	0.998	0.997
PRODB18	0.996	0.993	0.996	0.998	0.997

Covariance Coverage

	MAR18	GAD18	MDE18	EAT18	PTSD18
MAR18	1.000				
GAD18	1.000	1.000			
MDE18	0.999	0.999	0.999		
EAT18	0.999	0.999	0.998	0.999	
PTSD18	0.999	0.999	0.998	0.998	0.999
PRODA18	0.998	0.998	0.997	0.997	0.997
PRODB18	0.998	0.998	0.997	0.997	0.997

Covariance Coverage

	PRODA18	PRODB18
PRODA18	0.998	
PRODB18	0.998	0.998

SAMPLE STATISTICS

ESTIMATED SAMPLE STATISTICS

Means

SMK18	CD18	ADHD18	PSY18	ALC18
0.672	2.123	5.788	0.044	1.125

Means

MAR18	GAD18	MDE18	EAT18	PTSD18
0.335	1.134	1.809	0.453	1.365

Means

PRODA18	PRODB18
0.273	0.247

Covariances

	SMK18	CD18	ADHD18	PSY18	ALC18
SMK18	2.630				
CD18	1.183	5.205			
ADHD18	1.560	4.202	18.393		
PSY18	0.044	0.076	0.167	0.094	
ALC18	0.522	1.417	2.176	0.022	2.818
MAR18	0.839	1.260	1.265	0.048	0.442
GAD18	0.292	0.672	2.373	0.109	0.471
MDE18	0.933	1.595	4.348	0.153	1.282
EAT18	0.212	0.341	1.041	0.036	0.299
PTSD18	0.938	1.135	2.751	0.158	0.660
PRODA18	0.181	0.372	0.881	0.081	0.134
PRODB18	0.150	0.437	0.892	0.069	0.217

Covariances

	MAR18	GAD18	MDE18	EAT18	PTSD18
MAR18	1.891				
GAD18	0.241	4.627			
MDE18	0.689	2.941	8.835		
EAT18	0.107	0.505	0.917	0.779	
PTSD18	0.366	1.711	3.372	0.637	11.012
PRODA18	0.201	0.488	0.772	0.177	0.882
PRODB18	0.233	0.489	0.761	0.141	0.690

Covariances

	PRODA18	PRODB18
PRODA18	0.589	
PRODB18	0.293	0.584

Correlations

	SMK18	CD18	ADHD18	PSY18	ALC18
SMK18	1.000				
CD18	0.320	1.000			
ADHD18	0.224	0.429	1.000		
PSY18	0.089	0.109	0.127	1.000	
ALC18	0.192	0.370	0.302	0.044	1.000
MAR18	0.376	0.402	0.215	0.114	0.192
GAD18	0.084	0.137	0.257	0.166	0.131
MDE18	0.194	0.235	0.341	0.168	0.257
EAT18	0.148	0.169	0.275	0.135	0.202
PTSD18	0.174	0.150	0.193	0.156	0.118
PRODA18	0.145	0.212	0.268	0.346	0.104
PRODB18	0.121	0.251	0.272	0.294	0.169

Correlations

	MAR18	GAD18	MDE18	EAT18	PTSD18
MAR18	1.000				
GAD18	0.081	1.000			
MDE18	0.169	0.460	1.000		
EAT18	0.088	0.266	0.350	1.000	
PTSD18	0.080	0.240	0.342	0.218	1.000
PRODA18	0.191	0.296	0.339	0.262	0.346
PRODB18	0.222	0.298	0.335	0.210	0.272

Correlations

	PRODA18	PRODB18
PRODA18	1.000	
PRODB18	0.500	1.000

MAXIMUM LOG-LIKELIHOOD VALUE FOR THE UNRESTRICTED (H1) MODEL IS -42633.558

UNIVARIATE SAMPLE STATISTICS

UNIVARIATE HIGHER-ORDER MOMENT DESCRIPTIVE STATISTICS

Variable/ Sample Size	Mean/ Variance	Skewness/ Kurtosis	Minimum/ Maximum	% with Min/Max	Percentiles 20%/60%	40%/80%	Median
SMK18	0.670	2.585	0.000	80.94%	0.000	0.000	0.000
2062.000	2.625	6.106	10.000	0.05%	0.000	0.000	
CD18	2.121	1.262	0.000	30.54%	0.000	1.000	1.000
2053.000	5.209	1.205	11.000	0.24%	2.000	4.000	
ADHD18	5.787	0.510	0.000	10.92%	2.000	4.000	5.000
2061.000	18.399	-0.528	18.000	0.44%	7.000	10.000	
PSY18	0.044	9.848	0.000	97.14%	0.000	0.000	0.000
2063.000	0.094	125.741	6.000	0.05%	0.000	0.000	
ALC18	1.125	1.949	0.000	53.76%	0.000	0.000	0.000
2063.000	2.818	4.436	11.000	0.10%	1.000	2.000	
MAR18	0.335	4.751	0.000	92.40%	0.000	0.000	0.000
2066.000	1.891	23.548	10.000	0.44%	0.000	0.000	

```
     GAD18               1.134       1.593       0.000   75.46%       0.000      0.000      0.000
          2066.000       4.627       0.931       7.000    3.05%       0.000      3.000
     MDE18               1.806       1.273       0.000   68.93%       0.000      0.000      0.000
          2063.000       8.828      -0.017       9.000    3.34%       0.000      5.000
     EAT18               0.453       2.416       0.000   71.71%       0.000      0.000      0.000
          2064.000       0.779       6.256       5.000    0.44%       0.000      1.000
     PTSD18              1.365       2.642       0.000   79.41%       0.000      0.000      0.000
          2064.000      11.014       6.296      17.000    0.24%       0.000      1.000
     PRODA18             0.273       3.450       0.000   85.55%       0.000      0.000      0.000
          2062.000       0.588      14.002       6.000    0.19%       0.000      0.000
     PRODB18             0.247       3.734       0.000   87.83%       0.000      0.000      0.000
          2062.000       0.584      15.720       6.000    0.19%       0.000      0.000

THE MODEL ESTIMATION TERMINATED NORMALLY

MODEL FIT INFORMATION

Number of Free Parameters                       48

Loglikelihood

          H0 Value                      -42782.474
          H0 Scaling Correction Factor      4.1385
            for MLR
          H1 Value                      -42633.558
          H1 Scaling Correction Factor      3.0107
            for MLR

Information Criteria

          Akaike (AIC)                   85660.948
          Bayesian (BIC)                 85931.350
          Sample-Size Adjusted BIC       85778.850
            (n* = (n + 2) / 24)

Chi-Square Test of Model Fit

          Value                            172.973*
          Degrees of Freedom                    42
          P-Value                           0.0000
          Scaling Correction Factor         1.7218
            for MLR

*   The chi-square value for MLM, MLMV, MLR, ULSMV, WLSM and WLSMV cannot be used
    for chi-square difference testing in the regular way.  MLM, MLR and WLSM
    chi-square difference testing is described on the Mplus website.  MLMV, WLSMV,
    and ULSMV difference testing is done using the DIFFTEST option.

RMSEA (Root Mean Square Error Of Approximation)

          Estimate                           0.039
          90 Percent C.I.                    0.033  0.045
          Probability RMSEA <= .05           0.999

CFI/TLI

          CFI                                0.944
          TLI                                0.913

Chi-Square Test of Model Fit for the Baseline Model

          Value                           2418.883
          Degrees of Freedom                    66
          P-Value                           0.0000

SRMR (Standardized Root Mean Square Residual)

          Value                              0.031

MODEL RESULTS
```

	Estimate	S.E.	Est./S.E.	Two-Tailed P-Value
EXT BY				
ADHD18	1.389	0.145	9.587	0.000
ALC18	0.583	0.067	8.695	0.000
MAR18	0.644	0.097	6.660	0.000
SMK18	0.618	0.100	6.193	0.000
CD18	1.543	0.125	12.362	0.000
INT BY				
GAD18	0.495	0.917	0.540	0.589
MDE18	1.735	2.952	0.588	0.557
EAT18	0.068	0.162	0.422	0.673
PTSD18	0.130	0.161	0.804	0.421
THD BY				
PSY18	0.113	0.027	4.144	0.000
PRODA18	0.389	0.070	5.543	0.000
PRODB18	0.298	0.057	5.209	0.000
P BY				
ADHD18	2.153	0.125	17.209	0.000
ALC18	0.534	0.069	7.684	0.000
MAR18	0.343	0.072	4.765	0.000
SMK18	0.430	0.067	6.454	0.000
CD18	0.838	0.087	9.637	0.000
GAD18	1.083	0.098	11.091	0.000
MDE18	1.923	0.098	19.627	0.000
EAT18	0.417	0.049	8.573	0.000
PTSD18	1.622	0.184	8.819	0.000
PSY18	0.086	0.018	4.868	0.000
PRODA18	0.433	0.038	11.496	0.000
PRODB18	0.408	0.039	10.448	0.000
EXT WITH				
INT	0.000	0.000	999.000	999.000
THD	0.000	0.000	999.000	999.000
P	0.000	0.000	999.000	999.000
INT WITH				
THD	0.000	0.000	999.000	999.000
P	0.000	0.000	999.000	999.000
P WITH				
THD	0.000	0.000	999.000	999.000
Means				
EXT	0.000	0.000	999.000	999.000
INT	0.000	0.000	999.000	999.000
THD	0.000	0.000	999.000	999.000
P	0.000	0.000	999.000	999.000
Intercepts				
SMK18	0.672	0.044	15.381	0.000
CD18	2.123	0.061	34.826	0.000
ADHD18	5.788	0.105	54.976	0.000
PSY18	0.044	0.007	6.312	0.000
ALC18	1.125	0.042	26.783	0.000
MAR18	0.335	0.034	9.757	0.000
GAD18	1.134	0.052	22.005	0.000
MDE18	1.810	0.072	25.030	0.000
EAT18	0.453	0.022	20.714	0.000
PTSD18	1.365	0.082	16.739	0.000
PRODA18	0.273	0.018	15.100	0.000
PRODB18	0.247	0.018	13.687	0.000
Variances				
EXT	1.000	0.000	999.000	999.000

```
   INT               1.000      0.000    999.000    999.000
   THD               1.000      0.000    999.000    999.000
   P                 1.000      0.000    999.000    999.000

 Residual Variances
   SMK18             2.062      0.178     11.579      0.000
   CD18              2.123      0.335      6.339      0.000
   ADHD18           11.831      0.483     24.506      0.000
   PSY18             0.073      0.018      4.131      0.000
   ALC18             2.193      0.125     17.527      0.000
   MAR18             1.359      0.149      9.136      0.000
   GAD18             3.208      0.765      4.191      0.000
   MDE18             2.125     10.158      0.209      0.834
   EAT18             0.601      0.045     13.295      0.000
   PTSD18            8.364      0.630     13.280      0.000
   PRODA18           0.250      0.046      5.375      0.000
   PRODB18           0.328      0.040      8.281      0.000

QUALITY OF NUMERICAL RESULTS

     Condition Number for the Information Matrix              0.334E-06
       (ratio of smallest to largest eigenvalue)

STANDARDIZED MODEL RESULTS

STDYX Standardization
                                                        Two-Tailed
                    Estimate       S.E.  Est./S.E.    P-Value

 EXT      BY
   ADHD18            0.324      0.033      9.863      0.000
   ALC18             0.348      0.037      9.314      0.000
   MAR18             0.468      0.056      8.407      0.000
   SMK18             0.381      0.060      6.332      0.000
   CD18              0.676      0.052     12.950      0.000

 INT      BY
   GAD18             0.230      0.426      0.541      0.589
   MDE18             0.584      0.993      0.588      0.557
   EAT18             0.077      0.184      0.422      0.673
   PTSD18            0.039      0.049      0.804      0.422

 THD      BY
   PSY18             0.369      0.068      5.400      0.000
   PRODA18           0.508      0.085      5.986      0.000
   PRODB18           0.390      0.073      5.370      0.000

 P        BY
   ADHD18            0.502      0.028     18.030      0.000
   ALC18             0.318      0.037      8.486      0.000
   MAR18             0.249      0.046      5.368      0.000
   SMK18             0.265      0.038      6.916      0.000
   CD18              0.367      0.036     10.338      0.000
   GAD18             0.504      0.044     11.544      0.000
   MDE18             0.647      0.030     21.832      0.000
   EAT18             0.472      0.051      9.294      0.000
   PTSD18            0.489      0.048     10.250      0.000
   PSY18             0.280      0.033      8.451      0.000
   PRODA18           0.564      0.035     16.113      0.000
   PRODB18           0.535      0.036     14.809      0.000

 EXT      WITH
   INT               0.000      0.000    999.000    999.000
   THD               0.000      0.000    999.000    999.000
   P                 0.000      0.000    999.000    999.000

 INT      WITH
   THD               0.000      0.000    999.000    999.000
   P                 0.000      0.000    999.000    999.000
```

```
 P        WITH
    THD                0.000      0.000    999.000    999.000

 Means
    EXT                0.000      0.000    999.000    999.000
    INT                0.000      0.000    999.000    999.000
    THD                0.000      0.000    999.000    999.000
    P                  0.000      0.000    999.000    999.000

 Intercepts
    SMK18              0.414      0.014     29.283      0.000
    CD18               0.931      0.018     51.084      0.000
    ADHD18             1.350      0.023     57.498      0.000
    PSY18              0.144      0.011     12.740      0.000
    ALC18              0.670      0.015     43.284      0.000
    MAR18              0.244      0.012     20.944      0.000
    GAD18              0.527      0.014     36.593      0.000
    MDE18              0.609      0.015     39.361      0.000
    EAT18              0.513      0.013     39.853      0.000
    PTSD18             0.411      0.012     33.061      0.000
    PRODA18            0.356      0.012     29.048      0.000
    PRODB18            0.323      0.012     27.094      0.000

 Variances
    EXT                1.000      0.000    999.000    999.000
    INT                1.000      0.000    999.000    999.000
    THD                1.000      0.000    999.000    999.000
    P                  1.000      0.000    999.000    999.000

 Residual Variances
    SMK18              0.784      0.039     20.172      0.000
    CD18               0.408      0.064      6.404      0.000
    ADHD18             0.643      0.024     27.169      0.000
    PSY18              0.785      0.057     13.682      0.000
    ALC18              0.778      0.028     28.142      0.000
    MAR18              0.719      0.045     15.859      0.000
    GAD18              0.693      0.166      4.170      0.000
    MDE18              0.241      1.149      0.209      0.834
    EAT18              0.771      0.031     24.621      0.000
    PTSD18             0.760      0.044     17.127      0.000
    PRODA18            0.424      0.082      5.158      0.000
    PRODB18            0.562      0.057      9.892      0.000

STDY Standardization

                                                    Two-Tailed
                    Estimate       S.E.  Est./S.E.    P-Value

 EXT      BY
    ADHD18             0.324      0.033      9.863      0.000
    ALC18              0.348      0.037      9.314      0.000
    MAR18              0.468      0.056      8.407      0.000
    SMK18              0.381      0.060      6.332      0.000
    CD18               0.676      0.052     12.950      0.000

 INT      BY
    GAD18              0.230      0.426      0.541      0.589
    MDE18              0.584      0.993      0.588      0.557
    EAT18              0.077      0.184      0.422      0.673
    PTSD18             0.039      0.049      0.804      0.422

 THD      BY
    PSY18              0.369      0.068      5.400      0.000
    PRODA18            0.508      0.085      5.986      0.000
    PRODB18            0.390      0.073      5.370      0.000

 P        BY
    ADHD18             0.502      0.028     18.030      0.000
    ALC18              0.318      0.037      8.486      0.000
    MAR18              0.249      0.046      5.368      0.000
    SMK18              0.265      0.038      6.916      0.000
```

```
    CD18               0.367      0.036     10.338      0.000
    GAD18              0.504      0.044     11.544      0.000
    MDE18              0.647      0.030     21.832      0.000
    EAT18              0.472      0.051      9.294      0.000
    PTSD18             0.489      0.048     10.250      0.000
    PSY18              0.280      0.033      8.451      0.000
    PRODA18            0.564      0.035     16.113      0.000
    PRODB18            0.535      0.036     14.809      0.000

 EXT      WITH
    INT                0.000      0.000    999.000    999.000
    THD                0.000      0.000    999.000    999.000
    P                  0.000      0.000    999.000    999.000

 INT      WITH
    THD                0.000      0.000    999.000    999.000
    P                  0.000      0.000    999.000    999.000

 P        WITH
    THD                0.000      0.000    999.000    999.000

 Means
    EXT                0.000      0.000    999.000    999.000
    INT                0.000      0.000    999.000    999.000
    THD                0.000      0.000    999.000    999.000
    P                  0.000      0.000    999.000    999.000

 Intercepts
    SMK18              0.414      0.014     29.283      0.000
    CD18               0.931      0.018     51.084      0.000
    ADHD18             1.350      0.023     57.498      0.000
    PSY18              0.144      0.011     12.740      0.000
    ALC18              0.670      0.015     43.284      0.000
    MAR18              0.244      0.012     20.944      0.000
    GAD18              0.527      0.014     36.593      0.000
    MDE18              0.609      0.015     39.361      0.000
    EAT18              0.513      0.013     39.853      0.000
    PTSD18             0.411      0.012     33.061      0.000
    PRODA18            0.356      0.012     29.048      0.000
    PRODB18            0.323      0.012     27.094      0.000

 Variances
    EXT                1.000      0.000    999.000    999.000
    INT                1.000      0.000    999.000    999.000
    THD                1.000      0.000    999.000    999.000
    P                  1.000      0.000    999.000    999.000

 Residual Variances
    SMK18              0.784      0.039     20.172      0.000
    CD18               0.408      0.064      6.404      0.000
    ADHD18             0.643      0.024     27.169      0.000
    PSY18              0.785      0.057     13.682      0.000
    ALC18              0.778      0.028     28.142      0.000
    MAR18              0.719      0.045     15.859      0.000
    GAD18              0.693      0.166      4.170      0.000
    MDE18              0.241      1.149      0.209      0.834
    EAT18              0.771      0.031     24.621      0.000
    PTSD18             0.760      0.044     17.127      0.000
    PRODA18            0.424      0.082      5.158      0.000
    PRODB18            0.562      0.057      9.892      0.000

STD Standardization

                                                    Two-Tailed
                    Estimate       S.E.  Est./S.E.    P-Value

 EXT      BY
    ADHD18             1.389      0.145      9.587      0.000
    ALC18              0.583      0.067      8.695      0.000
    MAR18              0.644      0.097      6.660      0.000
    SMK18              0.618      0.100      6.193      0.000
```

```
   CD18               1.543      0.125     12.362      0.000

INT      BY
   GAD18              0.495      0.917      0.540      0.589
   MDE18              1.735      2.952      0.588      0.557
   EAT18              0.068      0.162      0.422      0.673
   PTSD18             0.130      0.161      0.804      0.421

THD      BY
   PSY18              0.113      0.027      4.144      0.000
   PRODA18            0.389      0.070      5.543      0.000
   PRODB18            0.298      0.057      5.209      0.000

P        BY
   ADHD18             2.153      0.125     17.209      0.000
   ALC18              0.534      0.069      7.684      0.000
   MAR18              0.343      0.072      4.765      0.000
   SMK18              0.430      0.067      6.454      0.000
   CD18               0.838      0.087      9.637      0.000
   GAD18              1.083      0.098     11.091      0.000
   MDE18              1.923      0.098     19.627      0.000
   EAT18              0.417      0.049      8.573      0.000
   PTSD18             1.622      0.184      8.819      0.000
   PSY18              0.086      0.018      4.868      0.000
   PRODA18            0.433      0.038     11.496      0.000
   PRODB18            0.408      0.039     10.448      0.000

EXT      WITH
   INT                0.000      0.000    999.000    999.000
   THD                0.000      0.000    999.000    999.000
   P                  0.000      0.000    999.000    999.000

INT      WITH
   THD                0.000      0.000    999.000    999.000
   P                  0.000      0.000    999.000    999.000

P        WITH
   THD                0.000      0.000    999.000    999.000

Means
   EXT                0.000      0.000    999.000    999.000
   INT                0.000      0.000    999.000    999.000
   THD                0.000      0.000    999.000    999.000
   P                  0.000      0.000    999.000    999.000

Intercepts
   SMK18              0.672      0.044     15.381      0.000
   CD18               2.123      0.061     34.826      0.000
   ADHD18             5.788      0.105     54.976      0.000
   PSY18              0.044      0.007      6.312      0.000
   ALC18              1.125      0.042     26.783      0.000
   MAR18              0.335      0.034      9.757      0.000
   GAD18              1.134      0.052     22.005      0.000
   MDE18              1.810      0.072     25.030      0.000
   EAT18              0.453      0.022     20.714      0.000
   PTSD18             1.365      0.082     16.739      0.000
   PRODA18            0.273      0.018     15.100      0.000
   PRODB18            0.247      0.018     13.687      0.000

Variances
   EXT                1.000      0.000    999.000    999.000
   INT                1.000      0.000    999.000    999.000
   THD                1.000      0.000    999.000    999.000
   P                  1.000      0.000    999.000    999.000

Residual Variances
   SMK18              2.062      0.178     11.579      0.000
   CD18               2.123      0.335      6.339      0.000
   ADHD18            11.831      0.483     24.506      0.000
   PSY18              0.073      0.018      4.131      0.000
```

ALC18	2.193	0.125	17.527	0.000
MAR18	1.359	0.149	9.136	0.000
GAD18	3.208	0.765	4.191	0.000
MDE18	2.125	10.158	0.209	0.834
EAT18	0.601	0.045	13.295	0.000
PTSD18	8.364	0.630	13.280	0.000
PRODA18	0.250	0.046	5.375	0.000
PRODB18	0.328	0.040	8.281	0.000

R-SQUARE

Observed Variable	Estimate	S.E.	Est./S.E.	Two-Tailed P-Value
SMK18	0.216	0.039	5.543	0.000
CD18	0.592	0.064	9.298	0.000
ADHD18	0.357	0.024	15.074	0.000
PSY18	0.215	0.057	3.741	0.000
ALC18	0.222	0.028	8.030	0.000
MAR18	0.281	0.045	6.205	0.000
GAD18	0.307	0.166	1.844	0.065
MDE18	0.759	1.149	0.661	0.509
EAT18	0.229	0.031	7.310	0.000
PTSD18	0.240	0.044	5.422	0.000
PRODA18	0.576	0.082	7.006	0.000
PRODB18	0.438	0.057	7.703	0.000

SUMMARY OF FACTOR SCORES

FACTOR SCORE INFORMATION (COMPLETE-DATA PATTERN)

FACTOR DETERMINACIES

EXT	0.777
INT	0.673
THD	0.648
P	0.847

FACTOR SCORE INFORMATION (PATTERN 2)

FACTOR DETERMINACIES

EXT	0.770
INT	0.650
THD	0.379
P	0.812

FACTOR SCORE INFORMATION (PATTERN 3)

FACTOR DETERMINACIES

EXT	0.774
INT	0.666
THD	0.641
P	0.833

FACTOR SCORE INFORMATION (PATTERN 4)

FACTOR DETERMINACIES

EXT	0.774
INT	0.670
THD	0.643
P	0.836

FACTOR SCORE INFORMATION (PATTERN 5)

FACTOR DETERMINACIES

EXT	0.771
INT	0.252
THD	0.636
P	0.820

FACTOR SCORE INFORMATION (PATTERN 6)

```
           FACTOR DETERMINACIES

           EXT        0.772
           INT        0.666
           THD        0.643
           P          0.836

     FACTOR SCORE INFORMATION (PATTERN 7)
           FACTOR DETERMINACIES

           EXT        0.637
           INT        0.672
           THD        0.647
           P          0.846

     FACTOR SCORE INFORMATION (PATTERN 8)
           FACTOR DETERMINACIES

           EXT        0.603
           INT        0.671
           THD        0.646
           P          0.844

     FACTOR SCORE INFORMATION (PATTERN 9)
           FACTOR DETERMINACIES

           EXT        0.630
           INT        0.645
           THD        0.000
           P          0.805

     FACTOR SCORE INFORMATION (PATTERN 10)
           FACTOR DETERMINACIES

           EXT        0.560
           INT        0.244
           THD        0.615
           P          0.769

     FACTOR SCORE INFORMATION (PATTERN 11)
           FACTOR DETERMINACIES

           EXT        0.605
           INT        0.632
           THD        0.000
           P          0.784

     FACTOR SCORE INFORMATION (PATTERN 12)
           FACTOR DETERMINACIES

           EXT        0.764
           INT        0.672
           THD        0.647
           P          0.846

     FACTOR SCORE INFORMATION (PATTERN 13)
           FACTOR DETERMINACIES

           EXT        0.754
           INT        0.671
           THD        0.647
           P          0.845

     Beginning Time:  09:57:11
        Ending Time:  09:57:11
       Elapsed Time:  00:00:00

MUTHEN & MUTHEN
3463 Stoner Ave.
Los Angeles, CA  90066
```

```
Mplus VERSION 8.7
MUTHEN & MUTHEN
10/27/2022   9:59 AM

INPUT INSTRUCTIONS

  TITLE:  E-Risk P Compare (Bifactor - Oblique);

  DATA:   FILE IS PCompare_Oct2020.dat;

  VARIABLE:
          NAMES ARE
              familyid atwinid rorder sex zygosity iqe5 seswq35 lowsc harm
              polyv512 aces512 famhist smk18 cd18 adhd18 psy18
              polyv18 CRP18 IL6_18 suPAR iq18 fsiq18
              alc18 mar18 gad18 mde18 eat18 ptsd18 prod18 prodA18 prodB18;

          MISSING
              ALL (999999);

          CLUSTER = familyid;

          USEVARIABLES ARE
              smk18 cd18 adhd18 psy18 alc18 mar18 gad18 mde18 eat18 PTSD18
              prodA18 prodB18;

          IDVARIABLE IS
              atwinid;

  ANALYSIS:
      TYPE      = COMPLEX;
      ESTIMATOR = MLR;
      MODEL     = NOCOVARIANCES;

  MODEL:

      ext BY adhd18* alc18 mar18 smk18 cd18;
      int BY gad18*  mde18 eat18 PTSD18;
      thd BY psy18*  prodA18 prodB18;

      p   BY adhd18* alc18 mar18 smk18 cd18 gad18 mde18 eat18 PTSD18 psy18
             prodA18 prodB18;

      [ext@0 int@0 thd@0 p@0];
       ext@1 int@1 thd@1 p@1;

      ext WITH int thd;
      int WITH thd;
      P   WITH ext@0 int@0 thd@0;

  OUTPUT: SAMPSTAT STANDARDIZED FSDETERMINACY PATTERNS;

  !SAVEDATA:
  !    FILE     = BifactorOblique.dat;
  !    SAVE     = FSCORES;
  !    MISSFLAG = 9999;

*** WARNING
  Data set contains cases with missing on all variables.
  These cases were not included in the analysis.
  Number of cases with missing on all variables:  166
   1 WARNING(S) FOUND IN THE INPUT INSTRUCTIONS

E-Risk P Compare (Bifactor - Oblique);

SUMMARY OF ANALYSIS

Number of groups                                                 1
Number of observations                                        2066
```

Number of dependent variables 12
Number of independent variables 0
Number of continuous latent variables 4

Observed dependent variables

Continuous

SMK18	CD18	ADHD18	PSY18	ALC18	MAR18
GAD18	MDE18	EAT18	PTSD18	PRODA18	PRODB18

Continuous latent variables

EXT	INT	THD	P

Variables with special functions

Cluster variable FAMILYID
ID variable ATWINID

Estimator	MLR
Information matrix	OBSERVED
Maximum number of iterations	1000
Convergence criterion	0.500D-04
Maximum number of steepest descent iterations	20
Maximum number of iterations for H1	2000
Convergence criterion for H1	0.100D-03

Input data file(s)
PCompare_Oct2020.dat

Input data format FREE

SUMMARY OF DATA

Number of missing data patterns 13
Number of clusters 1044

SUMMARY OF MISSING DATA PATTERNS

MISSING DATA PATTERNS (x = not missing)

	1	2	3	4	5	6	7	8	9	10	11	12	13
SMK18	x	x	x	x	x	x	x	x	x	x	x		
CD18	x	x	x	x	x	x						x	x
ADHD18	x	x	x	x	x		x	x	x			x	x
PSY18	x	x	x	x	x	x	x	x		x		x	x
ALC18	x	x	x	x	x	x	x		x		x	x	
MAR18	x	x	x	x	x	x	x	x	x	x	x	x	x
GAD18	x	x	x	x	x	x	x	x	x	x	x	x	x
MDE18	x	x	x	x		x	x	x	x		x	x	x
EAT18	x	x	x		x	x	x	x	x		x	x	x
PTSD18	x	x		x	x	x	x	x	x	x	x	x	x
PRODA18	x		x	x	x	x	x	x		x		x	x
PRODB18	x		x	x	x	x	x	x		x		x	x

MISSING DATA PATTERN FREQUENCIES

Pattern	Frequency	Pattern	Frequency	Pattern	Frequency
1	2040	6	3	11	1
2	1	7	8	12	3
3	2	8	1	13	1
4	1	9	2		
5	2	10	1		

COVARIANCE COVERAGE OF DATA

Minimum covariance coverage value 0.100

PROPORTION OF DATA PRESENT

Covariance Coverage

	SMK18	CD18	ADHD18	PSY18	ALC18
SMK18	0.998				
CD18	0.992	0.994			
ADHD18	0.996	0.992	0.998		
PSY18	0.997	0.994	0.997	0.999	
ALC18	0.997	0.993	0.997	0.997	0.999
MAR18	0.998	0.994	0.998	0.999	0.999
GAD18	0.998	0.994	0.998	0.999	0.999
MDE18	0.997	0.993	0.997	0.997	0.998
EAT18	0.997	0.993	0.997	0.998	0.998
PTSD18	0.997	0.993	0.997	0.998	0.998
PRODA18	0.996	0.993	0.996	0.998	0.997
PRODB18	0.996	0.993	0.996	0.998	0.997

Covariance Coverage

	MAR18	GAD18	MDE18	EAT18	PTSD18
MAR18	1.000				
GAD18	1.000	1.000			
MDE18	0.999	0.999	0.999		
EAT18	0.999	0.999	0.998	0.999	
PTSD18	0.999	0.999	0.998	0.998	0.999
PRODA18	0.998	0.998	0.997	0.997	0.997
PRODB18	0.998	0.998	0.997	0.997	0.997

Covariance Coverage

	PRODA18	PRODB18
PRODA18	0.998	
PRODB18	0.998	0.998

SAMPLE STATISTICS

ESTIMATED SAMPLE STATISTICS

Means

SMK18	CD18	ADHD18	PSY18	ALC18
0.672	2.123	5.788	0.044	1.125

Means

MAR18	GAD18	MDE18	EAT18	PTSD18
0.335	1.134	1.809	0.453	1.365

Means

PRODA18	PRODB18
0.273	0.247

Covariances

	SMK18	CD18	ADHD18	PSY18	ALC18
SMK18	2.630				
CD18	1.183	5.205			
ADHD18	1.560	4.202	18.393		
PSY18	0.044	0.076	0.167	0.094	
ALC18	0.522	1.417	2.176	0.022	2.818
MAR18	0.839	1.260	1.265	0.048	0.442
GAD18	0.292	0.672	2.373	0.109	0.471
MDE18	0.933	1.595	4.348	0.153	1.282
EAT18	0.212	0.341	1.041	0.036	0.299
PTSD18	0.938	1.135	2.751	0.158	0.660
PRODA18	0.181	0.372	0.881	0.081	0.134
PRODB18	0.150	0.437	0.892	0.069	0.217

Covariances

	MAR18	GAD18	MDE18	EAT18	PTSD18
MAR18	1.891				
GAD18	0.241	4.627			
MDE18	0.689	2.941	8.835		
EAT18	0.107	0.505	0.917	0.779	
PTSD18	0.366	1.711	3.372	0.637	11.012
PRODA18	0.201	0.488	0.772	0.177	0.882
PRODB18	0.233	0.489	0.761	0.141	0.690

Covariances

	PRODA18	PRODB18
PRODA18	0.589	
PRODB18	0.293	0.584

Correlations

	SMK18	CD18	ADHD18	PSY18	ALC18
SMK18	1.000				
CD18	0.320	1.000			
ADHD18	0.224	0.429	1.000		
PSY18	0.089	0.109	0.127	1.000	
ALC18	0.192	0.370	0.302	0.044	1.000
MAR18	0.376	0.402	0.215	0.114	0.192
GAD18	0.084	0.137	0.257	0.166	0.131
MDE18	0.194	0.235	0.341	0.168	0.257
EAT18	0.148	0.169	0.275	0.135	0.202
PTSD18	0.174	0.150	0.193	0.156	0.118
PRODA18	0.145	0.212	0.268	0.346	0.104
PRODB18	0.121	0.251	0.272	0.294	0.169

Correlations

	MAR18	GAD18	MDE18	EAT18	PTSD18
MAR18	1.000				
GAD18	0.081	1.000			
MDE18	0.169	0.460	1.000		
EAT18	0.088	0.266	0.350	1.000	
PTSD18	0.080	0.240	0.342	0.218	1.000
PRODA18	0.191	0.296	0.339	0.262	0.346
PRODB18	0.222	0.298	0.335	0.210	0.272

Correlations

	PRODA18	PRODB18
PRODA18	1.000	
PRODB18	0.500	1.000

MAXIMUM LOG-LIKELIHOOD VALUE FOR THE UNRESTRICTED (H1) MODEL IS -42633.558

UNIVARIATE SAMPLE STATISTICS

UNIVARIATE HIGHER-ORDER MOMENT DESCRIPTIVE STATISTICS

Variable/ Sample Size	Mean/ Variance	Skewness/ Kurtosis	Minimum/ Maximum	% with Min/Max	Percentiles 20%/60%	40%/80%	Median
SMK18	0.670	2.585	0.000	80.94%	0.000	0.000	0.000
2062.000	2.625	6.106	10.000	0.05%	0.000	0.000	
CD18	2.121	1.262	0.000	30.54%	0.000	1.000	1.000
2053.000	5.209	1.205	11.000	0.24%	2.000	4.000	
ADHD18	5.787	0.510	0.000	10.92%	2.000	4.000	5.000
2061.000	18.399	-0.528	18.000	0.44%	7.000	10.000	
PSY18	0.044	9.848	0.000	97.14%	0.000	0.000	0.000
2063.000	0.094	125.741	6.000	0.05%	0.000	0.000	
ALC18	1.125	1.949	0.000	53.76%	0.000	0.000	0.000
2063.000	2.818	4.436	11.000	0.10%	1.000	2.000	
MAR18	0.335	4.751	0.000	92.40%	0.000	0.000	0.000
2066.000	1.891	23.548	10.000	0.44%	0.000	0.000	

```
     GAD18                1.134       1.593       0.000   75.46%       0.000      0.000      0.000
           2066.000       4.627       0.931       7.000    3.05%       0.000      3.000
     MDE18                1.806       1.273       0.000   68.93%       0.000      0.000      0.000
           2063.000       8.828      -0.017       9.000    3.34%       0.000      5.000
     EAT18                0.453       2.416       0.000   71.71%       0.000      0.000      0.000
           2064.000       0.779       6.256       5.000    0.44%       0.000      1.000
     PTSD18               1.365       2.642       0.000   79.41%       0.000      0.000      0.000
           2064.000      11.014       6.296      17.000    0.24%       0.000      1.000
     PRODA18              0.273       3.450       0.000   85.55%       0.000      0.000      0.000
           2062.000       0.588      14.002       6.000    0.19%       0.000      0.000
     PRODB18              0.247       3.734       0.000   87.83%       0.000      0.000      0.000
           2062.000       0.584      15.720       6.000    0.19%       0.000      0.000

THE MODEL ESTIMATION TERMINATED NORMALLY

MODEL FIT INFORMATION

Number of Free Parameters                       51

Loglikelihood

          H0 Value                      -42736.080
          H0 Scaling Correction Factor      3.9118
            for MLR
          H1 Value                      -42633.558
          H1 Scaling Correction Factor      3.0107
            for MLR

Information Criteria

          Akaike (AIC)                   85574.160
          Bayesian (BIC)                 85861.461
          Sample-Size Adjusted BIC       85699.430
            (n* = (n + 2) / 24)

Chi-Square Test of Model Fit

          Value                            111.897*
          Degrees of Freedom                    39
          P-Value                           0.0000
          Scaling Correction Factor         1.8324
            for MLR

*   The chi-square value for MLM, MLMV, MLR, ULSMV, WLSM and WLSMV cannot be used
    for chi-square difference testing in the regular way.  MLM, MLR and WLSM
    chi-square difference testing is described on the Mplus website.  MLMV, WLSMV,
    and ULSMV difference testing is done using the DIFFTEST option.

RMSEA (Root Mean Square Error Of Approximation)

          Estimate                           0.030
          90 Percent C.I.                    0.024  0.037
          Probability RMSEA <= .05           1.000

CFI/TLI

          CFI                                0.969
          TLI                                0.948

Chi-Square Test of Model Fit for the Baseline Model

          Value                           2418.883
          Degrees of Freedom                    66
          P-Value                           0.0000

SRMR (Standardized Root Mean Square Residual)

          Value                              0.025

MODEL RESULTS
```

	Estimate	S.E.	Est./S.E.	Two-Tailed P-Value
EXT BY				
ADHD18	-0.334	0.837	-0.399	0.690
ALC18	0.013	0.208	0.063	0.950
MAR18	0.969	0.219	4.423	0.000
SMK18	0.517	0.198	2.616	0.009
CD18	0.509	0.394	1.290	0.197
INT BY				
GAD18	1.130	0.088	12.898	0.000
MDE18	1.621	0.115	14.067	0.000
EAT18	0.283	0.048	5.889	0.000
PTSD18	1.316	0.177	7.450	0.000
THD BY				
PSY18	0.129	0.028	4.666	0.000
PRODA18	0.526	0.048	11.032	0.000
PRODB18	0.423	0.045	9.418	0.000
P BY				
ADHD18	2.955	0.148	19.986	0.000
ALC18	0.835	0.066	12.683	0.000
MAR18	0.537	0.271	1.985	0.047
SMK18	0.622	0.146	4.255	0.000
CD18	1.454	0.155	9.362	0.000
GAD18	0.658	0.075	8.723	0.000
MDE18	1.397	0.096	14.572	0.000
EAT18	0.314	0.040	7.881	0.000
PTSD18	0.920	0.114	8.043	0.000
PSY18	0.048	0.013	3.689	0.000
PRODA18	0.254	0.033	7.690	0.000
PRODB18	0.285	0.038	7.463	0.000
EXT WITH				
INT	-0.101	0.214	-0.474	0.636
THD	0.133	0.165	0.807	0.420
P	0.000	0.000	999.000	999.000
INT WITH				
THD	0.577	0.060	9.661	0.000
P	0.000	0.000	999.000	999.000
P WITH				
THD	0.000	0.000	999.000	999.000
Means				
EXT	0.000	0.000	999.000	999.000
INT	0.000	0.000	999.000	999.000
THD	0.000	0.000	999.000	999.000
P	0.000	0.000	999.000	999.000
Intercepts				
SMK18	0.672	0.044	15.386	0.000
CD18	2.123	0.061	34.838	0.000
ADHD18	5.787	0.105	54.966	0.000
PSY18	0.044	0.007	6.313	0.000
ALC18	1.125	0.042	26.783	0.000
MAR18	0.335	0.034	9.757	0.000
GAD18	1.134	0.052	22.005	0.000
MDE18	1.810	0.072	25.033	0.000
EAT18	0.453	0.022	20.713	0.000
PTSD18	1.365	0.082	16.739	0.000
PRODA18	0.273	0.018	15.099	0.000
PRODB18	0.247	0.018	13.688	0.000
Variances				
EXT	1.000	0.000	999.000	999.000
INT	1.000	0.000	999.000	999.000

```
   THD                1.000      0.000    999.000    999.000
   P                  1.000      0.000    999.000    999.000

 Residual Variances
   SMK18              1.977      0.172     11.507      0.000
   CD18               2.833      0.189     14.951      0.000
   ADHD18             9.548      0.750     12.729      0.000
   PSY18              0.075      0.018      4.233      0.000
   ALC18              2.120      0.130     16.341      0.000
   MAR18              0.664      0.296      2.246      0.025
   GAD18              2.916      0.181     16.115      0.000
   MDE18              4.259      0.321     13.258      0.000
   EAT18              0.600      0.043     14.088      0.000
   PTSD18             8.433      0.543     15.539      0.000
   PRODA18            0.247      0.038      6.527      0.000
   PRODB18            0.324      0.036      9.030      0.000

QUALITY OF NUMERICAL RESULTS

     Condition Number for the Information Matrix              0.190E-04
       (ratio of smallest to largest eigenvalue)

STANDARDIZED MODEL RESULTS

STDYX Standardization
                                                    Two-Tailed
                    Estimate       S.E.  Est./S.E.    P-Value

 EXT      BY
   ADHD18            -0.078      0.195     -0.399      0.690
   ALC18              0.008      0.124      0.063      0.950
   MAR18              0.704      0.154      4.584      0.000
   SMK18              0.319      0.121      2.631      0.009
   CD18               0.223      0.173      1.292      0.196

 INT      BY
   GAD18              0.526      0.038     13.839      0.000
   MDE18              0.545      0.037     14.747      0.000
   EAT18              0.321      0.052      6.153      0.000
   PTSD18             0.397      0.047      8.521      0.000

 THD      BY
   PSY18              0.421      0.055      7.607      0.000
   PRODA18            0.686      0.048     14.439      0.000
   PRODB18            0.554      0.052     10.750      0.000

 P        BY
   ADHD18             0.689      0.031     21.899      0.000
   ALC18              0.498      0.032     15.656      0.000
   MAR18              0.390      0.192      2.032      0.042
   SMK18              0.384      0.089      4.328      0.000
   CD18               0.637      0.065      9.779      0.000
   GAD18              0.306      0.034      9.043      0.000
   MDE18              0.470      0.030     15.690      0.000
   EAT18              0.356      0.042      8.452      0.000
   PTSD18             0.277      0.031      8.849      0.000
   PSY18              0.156      0.037      4.194      0.000
   PRODA18            0.331      0.037      9.024      0.000
   PRODB18            0.372      0.041      9.021      0.000

 EXT      WITH
   INT               -0.101      0.214     -0.474      0.636
   THD                0.133      0.165      0.807      0.420
   P                  0.000      0.000    999.000    999.000

 INT      WITH
   THD                0.577      0.060      9.661      0.000
   P                  0.000      0.000    999.000    999.000

 P        WITH
```

```
    THD                0.000      0.000    999.000    999.000

 Means
    EXT                0.000      0.000    999.000    999.000
    INT                0.000      0.000    999.000    999.000
    THD                0.000      0.000    999.000    999.000
    P                  0.000      0.000    999.000    999.000

 Intercepts
    SMK18              0.414      0.014     29.295      0.000
    CD18               0.931      0.018     51.082      0.000
    ADHD18             1.349      0.023     57.498      0.000
    PSY18              0.144      0.011     12.732      0.000
    ALC18              0.670      0.015     43.280      0.000
    MAR18              0.244      0.012     20.944      0.000
    GAD18              0.527      0.014     36.592      0.000
    MDE18              0.609      0.015     39.370      0.000
    EAT18              0.513      0.013     39.857      0.000
    PTSD18             0.411      0.012     33.053      0.000
    PRODA18            0.356      0.012     29.058      0.000
    PRODB18            0.323      0.012     27.102      0.000

 Variances
    EXT                1.000      0.000    999.000    999.000
    INT                1.000      0.000    999.000    999.000
    THD                1.000      0.000    999.000    999.000
    P                  1.000      0.000    999.000    999.000

 Residual Variances
    SMK18              0.751      0.039     19.477      0.000
    CD18               0.544      0.035     15.415      0.000
    ADHD18             0.519      0.041     12.690      0.000
    PSY18              0.799      0.047     16.952      0.000
    ALC18              0.752      0.032     23.861      0.000
    MAR18              0.351      0.155      2.267      0.023
    GAD18              0.630      0.035     17.861      0.000
    MDE18              0.482      0.036     13.325      0.000
    EAT18              0.770      0.027     28.674      0.000
    PTSD18             0.766      0.037     20.830      0.000
    PRODA18            0.420      0.065      6.458      0.000
    PRODB18            0.555      0.048     11.600      0.000

STDY Standardization

                                                    Two-Tailed
                    Estimate       S.E.  Est./S.E.    P-Value

 EXT      BY
    ADHD18            -0.078      0.195     -0.399      0.690
    ALC18              0.008      0.124      0.063      0.950
    MAR18              0.704      0.154      4.584      0.000
    SMK18              0.319      0.121      2.631      0.009
    CD18               0.223      0.173      1.292      0.196

 INT      BY
    GAD18              0.526      0.038     13.839      0.000
    MDE18              0.545      0.037     14.747      0.000
    EAT18              0.321      0.052      6.153      0.000
    PTSD18             0.397      0.047      8.521      0.000

 THD      BY
    PSY18              0.421      0.055      7.607      0.000
    PRODA18            0.686      0.048     14.439      0.000
    PRODB18            0.554      0.052     10.750      0.000

 P        BY
    ADHD18             0.689      0.031     21.899      0.000
    ALC18              0.498      0.032     15.656      0.000
    MAR18              0.390      0.192      2.032      0.042
    SMK18              0.384      0.089      4.328      0.000
    CD18               0.637      0.065      9.779      0.000
```

```
   GAD18              0.306      0.034      9.043      0.000
   MDE18              0.470      0.030     15.690      0.000
   EAT18              0.356      0.042      8.452      0.000
   PTSD18             0.277      0.031      8.849      0.000
   PSY18              0.156      0.037      4.194      0.000
   PRODA18            0.331      0.037      9.024      0.000
   PRODB18            0.372      0.041      9.021      0.000

 EXT      WITH
   INT               -0.101      0.214     -0.474      0.636
   THD                0.133      0.165      0.807      0.420
   P                  0.000      0.000    999.000    999.000

 INT      WITH
   THD                0.577      0.060      9.661      0.000
   P                  0.000      0.000    999.000    999.000

 P        WITH
   THD                0.000      0.000    999.000    999.000

 Means
   EXT                0.000      0.000    999.000    999.000
   INT                0.000      0.000    999.000    999.000
   THD                0.000      0.000    999.000    999.000
   P                  0.000      0.000    999.000    999.000

 Intercepts
   SMK18              0.414      0.014     29.295      0.000
   CD18               0.931      0.018     51.082      0.000
   ADHD18             1.349      0.023     57.498      0.000
   PSY18              0.144      0.011     12.732      0.000
   ALC18              0.670      0.015     43.280      0.000
   MAR18              0.244      0.012     20.944      0.000
   GAD18              0.527      0.014     36.592      0.000
   MDE18              0.609      0.015     39.370      0.000
   EAT18              0.513      0.013     39.857      0.000
   PTSD18             0.411      0.012     33.053      0.000
   PRODA18            0.356      0.012     29.058      0.000
   PRODB18            0.323      0.012     27.102      0.000

 Variances
   EXT                1.000      0.000    999.000    999.000
   INT                1.000      0.000    999.000    999.000
   THD                1.000      0.000    999.000    999.000
   P                  1.000      0.000    999.000    999.000

 Residual Variances
   SMK18              0.751      0.039     19.477      0.000
   CD18               0.544      0.035     15.415      0.000
   ADHD18             0.519      0.041     12.690      0.000
   PSY18              0.799      0.047     16.952      0.000
   ALC18              0.752      0.032     23.861      0.000
   MAR18              0.351      0.155      2.267      0.023
   GAD18              0.630      0.035     17.861      0.000
   MDE18              0.482      0.036     13.325      0.000
   EAT18              0.770      0.027     28.674      0.000
   PTSD18             0.766      0.037     20.830      0.000
   PRODA18            0.420      0.065      6.458      0.000
   PRODB18            0.555      0.048     11.600      0.000

STD Standardization

                                                    Two-Tailed
                   Estimate       S.E.  Est./S.E.    P-Value

 EXT      BY
   ADHD18            -0.334      0.837     -0.399      0.690
   ALC18              0.013      0.208      0.063      0.950
   MAR18              0.969      0.219      4.423      0.000
   SMK18              0.517      0.198      2.616      0.009
   CD18               0.509      0.394      1.290      0.197
```

```
INT      BY
   GAD18             1.130      0.088     12.898      0.000
   MDE18             1.621      0.115     14.067      0.000
   EAT18             0.283      0.048      5.889      0.000
   PTSD18            1.316      0.177      7.450      0.000

THD      BY
   PSY18             0.129      0.028      4.666      0.000
   PRODA18           0.526      0.048     11.032      0.000
   PRODB18           0.423      0.045      9.418      0.000

P        BY
   ADHD18            2.955      0.148     19.986      0.000
   ALC18             0.835      0.066     12.683      0.000
   MAR18             0.537      0.271      1.985      0.047
   SMK18             0.622      0.146      4.255      0.000
   CD18              1.454      0.155      9.362      0.000
   GAD18             0.658      0.075      8.723      0.000
   MDE18             1.397      0.096     14.572      0.000
   EAT18             0.314      0.040      7.881      0.000
   PTSD18            0.920      0.114      8.043      0.000
   PSY18             0.048      0.013      3.689      0.000
   PRODA18           0.254      0.033      7.690      0.000
   PRODB18           0.285      0.038      7.463      0.000

EXT      WITH
   INT              -0.101      0.214     -0.474      0.636
   THD               0.133      0.165      0.807      0.420
   P                 0.000      0.000    999.000    999.000

INT      WITH
   THD               0.577      0.060      9.661      0.000
   P                 0.000      0.000    999.000    999.000

P        WITH
   THD               0.000      0.000    999.000    999.000

Means
   EXT               0.000      0.000    999.000    999.000
   INT               0.000      0.000    999.000    999.000
   THD               0.000      0.000    999.000    999.000
   P                 0.000      0.000    999.000    999.000

Intercepts
   SMK18             0.672      0.044     15.386      0.000
   CD18              2.123      0.061     34.838      0.000
   ADHD18            5.787      0.105     54.966      0.000
   PSY18             0.044      0.007      6.313      0.000
   ALC18             1.125      0.042     26.783      0.000
   MAR18             0.335      0.034      9.757      0.000
   GAD18             1.134      0.052     22.005      0.000
   MDE18             1.810      0.072     25.033      0.000
   EAT18             0.453      0.022     20.713      0.000
   PTSD18            1.365      0.082     16.739      0.000
   PRODA18           0.273      0.018     15.099      0.000
   PRODB18           0.247      0.018     13.688      0.000

Variances
   EXT               1.000      0.000    999.000    999.000
   INT               1.000      0.000    999.000    999.000
   THD               1.000      0.000    999.000    999.000
   P                 1.000      0.000    999.000    999.000

Residual Variances
   SMK18             1.977      0.172     11.507      0.000
   CD18              2.833      0.189     14.951      0.000
   ADHD18            9.548      0.750     12.729      0.000
   PSY18             0.075      0.018      4.233      0.000
   ALC18             2.120      0.130     16.341      0.000
```

MAR18	0.664	0.296	2.246	0.025
GAD18	2.916	0.181	16.115	0.000
MDE18	4.259	0.321	13.258	0.000
EAT18	0.600	0.043	14.088	0.000
PTSD18	8.433	0.543	15.539	0.000
PRODA18	0.247	0.038	6.527	0.000
PRODB18	0.324	0.036	9.030	0.000

R-SQUARE

Observed Variable	Estimate	S.E.	Est./S.E.	Two-Tailed P-Value
SMK18	0.249	0.039	6.444	0.000
CD18	0.456	0.035	12.914	0.000
ADHD18	0.481	0.041	11.753	0.000
PSY18	0.201	0.047	4.276	0.000
ALC18	0.248	0.032	7.852	0.000
MAR18	0.649	0.155	4.187	0.000
GAD18	0.370	0.035	10.477	0.000
MDE18	0.518	0.036	14.326	0.000
EAT18	0.230	0.027	8.548	0.000
PTSD18	0.234	0.037	6.370	0.000
PRODA18	0.580	0.065	8.916	0.000
PRODB18	0.445	0.048	9.310	0.000

SUMMARY OF FACTOR SCORES

FACTOR SCORE INFORMATION (COMPLETE-DATA PATTERN)

FACTOR DETERMINACIES

EXT	0.762
INT	0.754
THD	0.793
P	0.851

FACTOR SCORE INFORMATION (PATTERN 2)

FACTOR DETERMINACIES

EXT	0.761
INT	0.737
THD	0.545
P	0.849

FACTOR SCORE INFORMATION (PATTERN 3)

FACTOR DETERMINACIES

EXT	0.762
INT	0.737
THD	0.792
P	0.851

FACTOR SCORE INFORMATION (PATTERN 4)

FACTOR DETERMINACIES

EXT	0.761
INT	0.747
THD	0.793
P	0.849

FACTOR SCORE INFORMATION (PATTERN 5)

FACTOR DETERMINACIES

EXT	0.759
INT	0.696
THD	0.791
P	0.845

FACTOR SCORE INFORMATION (PATTERN 6)

FACTOR DETERMINACIES

```
          EXT        0.740
          INT        0.740
          THD        0.779
          P          0.787

     FACTOR SCORE INFORMATION (PATTERN 7)
          FACTOR DETERMINACIES

          EXT        0.761
          INT        0.746
          THD        0.787
          P          0.821

     FACTOR SCORE INFORMATION (PATTERN 8)
          FACTOR DETERMINACIES

          EXT        0.759
          INT        0.740
          THD        0.782
          P          0.797

     FACTOR SCORE INFORMATION (PATTERN 9)
          FACTOR DETERMINACIES

          EXT        0.760
          INT        0.721
          THD        0.421
          P          0.817

     FACTOR SCORE INFORMATION (PATTERN 10)
          FACTOR DETERMINACIES

          EXT        0.721
          INT        0.653
          THD        0.755
          P          0.594

     FACTOR SCORE INFORMATION (PATTERN 11)
          FACTOR DETERMINACIES

          EXT        0.739
          INT        0.700
          THD        0.398
          P          0.712

     FACTOR SCORE INFORMATION (PATTERN 12)
          FACTOR DETERMINACIES

          EXT        0.754
          INT        0.753
          THD        0.792
          P          0.847

     FACTOR SCORE INFORMATION (PATTERN 13)
          FACTOR DETERMINACIES

          EXT        0.752
          INT        0.749
          THD        0.789
          P          0.830

     Beginning Time:  09:59:25
        Ending Time:  09:59:25
       Elapsed Time:  00:00:00

MUTHEN & MUTHEN
3463 Stoner Ave.
Los Angeles, CA  90066
```

```
Mplus VERSION 8.7
MUTHEN & MUTHEN
10/27/2022   9:54 AM

INPUT INSTRUCTIONS

  TITLE:  E-Risk P Compare (Bifactor - Externalizing);

  DATA:   FILE IS PCompare_Oct2020.dat;

  VARIABLE:
          NAMES ARE
              familyid atwinid rorder sex zygosity iqe5 seswq35 lowsc harm
              polyv512 aces512 famhist smk18 cd18 adhd18 psy18
              polyv18 CRP18 IL6_18 suPAR iq18 fsiq18
              alc18 mar18 gad18 mde18 eat18 ptsd18 prod18 prodA18 prodB18;

          MISSING
              ALL (999999);

          CLUSTER = familyid;

          USEVARIABLES ARE
              smk18 cd18 adhd18 psy18 alc18 mar18 gad18 mde18 eat18 PTSD18
              prodA18 prodB18;

          IDVARIABLE IS
              atwinid;

  ANALYSIS:
      TYPE      = COMPLEX;
      ESTIMATOR = MLR;
      MODEL     = NOCOVARIANCES;

  MODEL:

      int BY gad18*  mde18 eat18 PTSD18;
      thd BY psy18*  prodA18 prodB18;

      p   BY adhd18* alc18 mar18 smk18 cd18 gad18 mde18 eat18 PTSD18 psy18
             prodA18 prodB18;

      [int@0 thd@0 p@0];
       int@1 thd@1 p@1;

      int WITH thd@0;
      P   WITH int@0 thd@0;

  OUTPUT: SAMPSTAT STANDARDIZED FSDETERMINACY PATTERNS;

  !SAVEDATA:
  !    FILE     = BifactorS-Ext.dat;
  !    SAVE     = FSCORES;
  !    MISSFLAG = 9999;

*** WARNING
  Data set contains cases with missing on all variables.
  These cases were not included in the analysis.
  Number of cases with missing on all variables:  166
   1 WARNING(S) FOUND IN THE INPUT INSTRUCTIONS

E-Risk P Compare (Bifactor - Externalizing);

SUMMARY OF ANALYSIS

Number of groups                                                 1
Number of observations                                        2066

Number of dependent variables                                   12
Number of independent variables                                  0
```

Number of continuous latent variables 3

Observed dependent variables

Continuous

SMK18	CD18	ADHD18	PSY18	ALC18	MAR18
GAD18	MDE18	EAT18	PTSD18	PRODA18	PRODB18

Continuous latent variables

INT THD P

Variables with special functions

Cluster variable FAMILYID
ID variable ATWINID

Estimator	MLR
Information matrix	OBSERVED
Maximum number of iterations	1000
Convergence criterion	0.500D-04
Maximum number of steepest descent iterations	20
Maximum number of iterations for H1	2000
Convergence criterion for H1	0.100D-03

Input data file(s)
PCompare_Oct2020.dat

Input data format FREE

SUMMARY OF DATA

Number of missing data patterns 13
Number of clusters 1044

SUMMARY OF MISSING DATA PATTERNS

MISSING DATA PATTERNS (x = not missing)

	1	2	3	4	5	6	7	8	9	10	11	12	13
SMK18	x	x	x	x	x	x	x	x	x	x	x		
CD18	x	x	x	x	x	x						x	x
ADHD18	x	x	x	x	x		x	x	x			x	x
PSY18	x	x	x	x	x	x	x	x		x		x	x
ALC18	x	x	x	x	x	x	x		x		x	x	
MAR18	x	x	x	x	x	x	x	x	x	x	x	x	x
GAD18	x	x	x	x	x	x	x	x	x	x	x	x	x
MDE18	x	x	x	x		x	x	x	x		x	x	x
EAT18	x	x	x		x	x	x	x	x		x	x	x
PTSD18	x	x		x	x	x	x	x	x	x	x	x	x
PRODA18	x		x	x	x	x	x	x		x		x	x
PRODB18	x		x	x	x	x	x	x		x		x	x

MISSING DATA PATTERN FREQUENCIES

Pattern	Frequency	Pattern	Frequency	Pattern	Frequency
1	2040	6	3	11	1
2	1	7	8	12	3
3	2	8	1	13	1
4	1	9	2		
5	2	10	1		

COVARIANCE COVERAGE OF DATA

Minimum covariance coverage value 0.100

PROPORTION OF DATA PRESENT

Covariance Coverage

SMK18	CD18	ADHD18	PSY18	ALC18

SMK18	0.998				
CD18	0.992	0.994			
ADHD18	0.996	0.992	0.998		
PSY18	0.997	0.994	0.997	0.999	
ALC18	0.997	0.993	0.997	0.997	0.999
MAR18	0.998	0.994	0.998	0.999	0.999
GAD18	0.998	0.994	0.998	0.999	0.999
MDE18	0.997	0.993	0.997	0.997	0.998
EAT18	0.997	0.993	0.997	0.998	0.998
PTSD18	0.997	0.993	0.997	0.998	0.998
PRODA18	0.996	0.993	0.996	0.998	0.997
PRODB18	0.996	0.993	0.996	0.998	0.997

Covariance Coverage

	MAR18	GAD18	MDE18	EAT18	PTSD18
MAR18	1.000				
GAD18	1.000	1.000			
MDE18	0.999	0.999	0.999		
EAT18	0.999	0.999	0.998	0.999	
PTSD18	0.999	0.999	0.998	0.998	0.999
PRODA18	0.998	0.998	0.997	0.997	0.997
PRODB18	0.998	0.998	0.997	0.997	0.997

Covariance Coverage

	PRODA18	PRODB18
PRODA18	0.998	
PRODB18	0.998	0.998

SAMPLE STATISTICS

ESTIMATED SAMPLE STATISTICS

Means

SMK18	CD18	ADHD18	PSY18	ALC18
0.672	2.123	5.788	0.044	1.125

Means

MAR18	GAD18	MDE18	EAT18	PTSD18
0.335	1.134	1.809	0.453	1.365

Means

PRODA18	PRODB18
0.273	0.247

Covariances

	SMK18	CD18	ADHD18	PSY18	ALC18
SMK18	2.630				
CD18	1.183	5.205			
ADHD18	1.560	4.202	18.393		
PSY18	0.044	0.076	0.167	0.094	
ALC18	0.522	1.417	2.176	0.022	2.818
MAR18	0.839	1.260	1.265	0.048	0.442
GAD18	0.292	0.672	2.373	0.109	0.471
MDE18	0.933	1.595	4.348	0.153	1.282
EAT18	0.212	0.341	1.041	0.036	0.299
PTSD18	0.938	1.135	2.751	0.158	0.660
PRODA18	0.181	0.372	0.881	0.081	0.134
PRODB18	0.150	0.437	0.892	0.069	0.217

Covariances

	MAR18	GAD18	MDE18	EAT18	PTSD18
MAR18	1.891				
GAD18	0.241	4.627			

MDE18	0.689	2.941	8.835		
EAT18	0.107	0.505	0.917	0.779	
PTSD18	0.366	1.711	3.372	0.637	11.012
PRODA18	0.201	0.488	0.772	0.177	0.882
PRODB18	0.233	0.489	0.761	0.141	0.690

Covariances

	PRODA18	PRODB18
PRODA18	0.589	
PRODB18	0.293	0.584

Correlations

	SMK18	CD18	ADHD18	PSY18	ALC18
SMK18	1.000				
CD18	0.320	1.000			
ADHD18	0.224	0.429	1.000		
PSY18	0.089	0.109	0.127	1.000	
ALC18	0.192	0.370	0.302	0.044	1.000
MAR18	0.376	0.402	0.215	0.114	0.192
GAD18	0.084	0.137	0.257	0.166	0.131
MDE18	0.194	0.235	0.341	0.168	0.257
EAT18	0.148	0.169	0.275	0.135	0.202
PTSD18	0.174	0.150	0.193	0.156	0.118
PRODA18	0.145	0.212	0.268	0.346	0.104
PRODB18	0.121	0.251	0.272	0.294	0.169

Correlations

	MAR18	GAD18	MDE18	EAT18	PTSD18
MAR18	1.000				
GAD18	0.081	1.000			
MDE18	0.169	0.460	1.000		
EAT18	0.088	0.266	0.350	1.000	
PTSD18	0.080	0.240	0.342	0.218	1.000
PRODA18	0.191	0.296	0.339	0.262	0.346
PRODB18	0.222	0.298	0.335	0.210	0.272

Correlations

	PRODA18	PRODB18
PRODA18	1.000	
PRODB18	0.500	1.000

MAXIMUM LOG-LIKELIHOOD VALUE FOR THE UNRESTRICTED (H1) MODEL IS -42633.558

UNIVARIATE SAMPLE STATISTICS

UNIVARIATE HIGHER-ORDER MOMENT DESCRIPTIVE STATISTICS

Variable/ Sample Size	Mean/ Variance	Skewness/ Kurtosis	Minimum/ Maximum	% with Min/Max	Percentiles 20%/60%	40%/80%	Median
SMK18	0.670	2.585	0.000	80.94%	0.000	0.000	0.000
2062.000	2.625	6.106	10.000	0.05%	0.000	0.000	
CD18	2.121	1.262	0.000	30.54%	0.000	1.000	1.000
2053.000	5.209	1.205	11.000	0.24%	2.000	4.000	
ADHD18	5.787	0.510	0.000	10.92%	2.000	4.000	5.000
2061.000	18.399	-0.528	18.000	0.44%	7.000	10.000	
PSY18	0.044	9.848	0.000	97.14%	0.000	0.000	0.000
2063.000	0.094	125.741	6.000	0.05%	0.000	0.000	
ALC18	1.125	1.949	0.000	53.76%	0.000	0.000	0.000
2063.000	2.818	4.436	11.000	0.10%	1.000	2.000	
MAR18	0.335	4.751	0.000	92.40%	0.000	0.000	0.000
2066.000	1.891	23.548	10.000	0.44%	0.000	0.000	
GAD18	1.134	1.593	0.000	75.46%	0.000	0.000	0.000
2066.000	4.627	0.931	7.000	3.05%	0.000	3.000	
MDE18	1.806	1.273	0.000	68.93%	0.000	0.000	0.000

```
        2063.000       8.828      -0.017       9.000    3.34%       0.000      5.000
     EAT18             0.453       2.416       0.000   71.71%       0.000      0.000      0.000
        2064.000       0.779       6.256       5.000    0.44%       0.000      1.000
     PTSD18            1.365       2.642       0.000   79.41%       0.000      0.000      0.000
        2064.000      11.014       6.296      17.000    0.24%       0.000      1.000
     PRODA18           0.273       3.450       0.000   85.55%       0.000      0.000      0.000
        2062.000       0.588      14.002       6.000    0.19%       0.000      0.000
     PRODB18           0.247       3.734       0.000   87.83%       0.000      0.000      0.000
        2062.000       0.584      15.720       6.000    0.19%       0.000      0.000

THE MODEL ESTIMATION TERMINATED NORMALLY

MODEL FIT INFORMATION

Number of Free Parameters                       43

Loglikelihood

          H0 Value                      -42955.153
          H0 Scaling Correction Factor      4.2678
            for MLR
          H1 Value                      -42633.558
          H1 Scaling Correction Factor      3.0107
            for MLR

Information Criteria

          Akaike (AIC)                   85996.306
          Bayesian (BIC)                 86238.541
          Sample-Size Adjusted BIC       86101.926
            (n* = (n + 2) / 24)

Chi-Square Test of Model Fit

          Value                            345.687*
          Degrees of Freedom                    47
          P-Value                           0.0000
          Scaling Correction Factor         1.8606
            for MLR

*   The chi-square value for MLM, MLMV, MLR, ULSMV, WLSM and WLSMV cannot be used
    for chi-square difference testing in the regular way.  MLM, MLR and WLSM
    chi-square difference testing is described on the Mplus website.  MLMV, WLSMV,
    and ULSMV difference testing is done using the DIFFTEST option.

RMSEA (Root Mean Square Error Of Approximation)

          Estimate                           0.055
          90 Percent C.I.                    0.050  0.061
          Probability RMSEA <= .05           0.048

CFI/TLI

          CFI                                0.873
          TLI                                0.822

Chi-Square Test of Model Fit for the Baseline Model

          Value                           2418.883
          Degrees of Freedom                    66
          P-Value                           0.0000

SRMR (Standardized Root Mean Square Residual)

          Value                              0.060

MODEL RESULTS
                                                    Two-Tailed
                    Estimate       S.E.  Est./S.E.    P-Value
```

INT BY				
GAD18	1.064	0.099	10.773	0.000
MDE18	1.820	0.139	13.073	0.000
EAT18	0.263	0.036	7.285	0.000
PTSD18	0.993	0.142	6.999	0.000
THD BY				
PSY18	0.123	0.027	4.508	0.000
PRODA18	0.486	0.065	7.513	0.000
PRODB18	0.377	0.051	7.453	0.000
P BY				
ADHD18	2.628	0.107	24.537	0.000
ALC18	0.796	0.063	12.575	0.000
MAR18	0.666	0.081	8.269	0.000
SMK18	0.728	0.060	12.095	0.000
CD18	1.511	0.086	17.589	0.000
GAD18	0.689	0.077	8.914	0.000
MDE18	1.441	0.100	14.347	0.000
EAT18	0.314	0.031	10.206	0.000
PTSD18	1.069	0.134	7.950	0.000
PSY18	0.066	0.016	4.202	0.000
PRODA18	0.323	0.034	9.546	0.000
PRODB18	0.339	0.036	9.538	0.000
INT WITH				
THD	0.000	0.000	999.000	999.000
P	0.000	0.000	999.000	999.000
P WITH				
THD	0.000	0.000	999.000	999.000
Means				
INT	0.000	0.000	999.000	999.000
THD	0.000	0.000	999.000	999.000
P	0.000	0.000	999.000	999.000
Intercepts				
SMK18	0.672	0.044	15.379	0.000
CD18	2.125	0.061	34.837	0.000
ADHD18	5.789	0.105	54.979	0.000
PSY18	0.044	0.007	6.313	0.000
ALC18	1.125	0.042	26.783	0.000
MAR18	0.335	0.034	9.757	0.000
GAD18	1.134	0.052	22.005	0.000
MDE18	1.809	0.072	25.018	0.000
EAT18	0.452	0.022	20.714	0.000
PTSD18	1.365	0.082	16.739	0.000
PRODA18	0.273	0.018	15.100	0.000
PRODB18	0.247	0.018	13.689	0.000
Variances				
INT	1.000	0.000	999.000	999.000
THD	1.000	0.000	999.000	999.000
P	1.000	0.000	999.000	999.000
Residual Variances				
SMK18	2.098	0.159	13.215	0.000
CD18	2.927	0.206	14.237	0.000
ADHD18	11.489	0.519	22.149	0.000
PSY18	0.074	0.018	4.115	0.000
ALC18	2.185	0.125	17.541	0.000
MAR18	1.447	0.152	9.491	0.000
GAD18	3.020	0.201	15.038	0.000
MDE18	3.443	0.454	7.585	0.000
EAT18	0.611	0.043	14.143	0.000
PTSD18	8.882	0.562	15.798	0.000
PRODA18	0.248	0.051	4.862	0.000
PRODB18	0.327	0.042	7.761	0.000

QUALITY OF NUMERICAL RESULTS

Condition Number for the Information Matrix 0.129E-03
(ratio of smallest to largest eigenvalue)

STANDARDIZED MODEL RESULTS

STDYX Standardization

	Estimate	S.E.	Est./S.E.	Two-Tailed P-Value
INT BY				
GAD18	0.495	0.044	11.344	0.000
MDE18	0.612	0.045	13.542	0.000
EAT18	0.298	0.038	7.837	0.000
PTSD18	0.299	0.039	7.600	0.000
THD BY				
PSY18	0.403	0.067	6.039	0.000
PRODA18	0.634	0.074	8.589	0.000
PRODB18	0.493	0.061	8.081	0.000
P BY				
ADHD18	0.613	0.022	28.252	0.000
ALC18	0.474	0.030	15.884	0.000
MAR18	0.485	0.036	13.437	0.000
SMK18	0.449	0.030	14.767	0.000
CD18	0.662	0.030	22.058	0.000
GAD18	0.320	0.034	9.297	0.000
MDE18	0.485	0.031	15.640	0.000
EAT18	0.356	0.031	11.462	0.000
PTSD18	0.322	0.036	9.020	0.000
PSY18	0.214	0.032	6.800	0.000
PRODA18	0.421	0.035	12.110	0.000
PRODB18	0.444	0.034	13.045	0.000
INT WITH				
THD	0.000	0.000	999.000	999.000
P	0.000	0.000	999.000	999.000
P WITH				
THD	0.000	0.000	999.000	999.000
Means				
INT	0.000	0.000	999.000	999.000
THD	0.000	0.000	999.000	999.000
P	0.000	0.000	999.000	999.000
Intercepts				
SMK18	0.414	0.014	29.282	0.000
CD18	0.931	0.018	51.092	0.000
ADHD18	1.350	0.023	57.498	0.000
PSY18	0.144	0.011	12.743	0.000
ALC18	0.670	0.015	43.288	0.000
MAR18	0.244	0.012	20.944	0.000
GAD18	0.527	0.014	36.592	0.000
MDE18	0.609	0.015	39.347	0.000
EAT18	0.513	0.013	39.859	0.000
PTSD18	0.411	0.012	33.062	0.000
PRODA18	0.356	0.012	29.063	0.000
PRODB18	0.323	0.012	27.106	0.000
Variances				
INT	1.000	0.000	999.000	999.000
THD	1.000	0.000	999.000	999.000
P	1.000	0.000	999.000	999.000
Residual Variances				
SMK18	0.798	0.027	29.227	0.000
CD18	0.562	0.040	14.141	0.000

ADHD18	0.624	0.027	23.491	0.000
PSY18	0.792	0.054	14.803	0.000
ALC18	0.775	0.028	27.386	0.000
MAR18	0.765	0.035	21.879	0.000
GAD18	0.653	0.040	16.271	0.000
MDE18	0.390	0.052	7.486	0.000
EAT18	0.785	0.026	30.665	0.000
PTSD18	0.807	0.027	29.844	0.000
PRODA18	0.421	0.090	4.684	0.000
PRODB18	0.560	0.062	8.998	0.000

STDY Standardization

	Estimate	S.E.	Est./S.E.	Two-Tailed P-Value
INT BY				
GAD18	0.495	0.044	11.344	0.000
MDE18	0.612	0.045	13.542	0.000
EAT18	0.298	0.038	7.837	0.000
PTSD18	0.299	0.039	7.600	0.000
THD BY				
PSY18	0.403	0.067	6.039	0.000
PRODA18	0.634	0.074	8.589	0.000
PRODB18	0.493	0.061	8.081	0.000
P BY				
ADHD18	0.613	0.022	28.252	0.000
ALC18	0.474	0.030	15.884	0.000
MAR18	0.485	0.036	13.437	0.000
SMK18	0.449	0.030	14.767	0.000
CD18	0.662	0.030	22.058	0.000
GAD18	0.320	0.034	9.297	0.000
MDE18	0.485	0.031	15.640	0.000
EAT18	0.356	0.031	11.462	0.000
PTSD18	0.322	0.036	9.020	0.000
PSY18	0.214	0.032	6.800	0.000
PRODA18	0.421	0.035	12.110	0.000
PRODB18	0.444	0.034	13.045	0.000
INT WITH				
THD	0.000	0.000	999.000	999.000
P	0.000	0.000	999.000	999.000
P WITH				
THD	0.000	0.000	999.000	999.000
Means				
INT	0.000	0.000	999.000	999.000
THD	0.000	0.000	999.000	999.000
P	0.000	0.000	999.000	999.000
Intercepts				
SMK18	0.414	0.014	29.282	0.000
CD18	0.931	0.018	51.092	0.000
ADHD18	1.350	0.023	57.498	0.000
PSY18	0.144	0.011	12.743	0.000
ALC18	0.670	0.015	43.288	0.000
MAR18	0.244	0.012	20.944	0.000
GAD18	0.527	0.014	36.592	0.000
MDE18	0.609	0.015	39.347	0.000
EAT18	0.513	0.013	39.859	0.000
PTSD18	0.411	0.012	33.062	0.000
PRODA18	0.356	0.012	29.063	0.000
PRODB18	0.323	0.012	27.106	0.000
Variances				
INT	1.000	0.000	999.000	999.000
THD	1.000	0.000	999.000	999.000
P	1.000	0.000	999.000	999.000

```
 Residual Variances
    SMK18              0.798      0.027     29.227      0.000
    CD18               0.562      0.040     14.141      0.000
    ADHD18             0.624      0.027     23.491      0.000
    PSY18              0.792      0.054     14.803      0.000
    ALC18              0.775      0.028     27.386      0.000
    MAR18              0.765      0.035     21.879      0.000
    GAD18              0.653      0.040     16.271      0.000
    MDE18              0.390      0.052      7.486      0.000
    EAT18              0.785      0.026     30.665      0.000
    PTSD18             0.807      0.027     29.844      0.000
    PRODA18            0.421      0.090      4.684      0.000
    PRODB18            0.560      0.062      8.998      0.000

STD Standardization
                                                    Two-Tailed
                    Estimate       S.E.  Est./S.E.    P-Value

 INT      BY
    GAD18              1.064      0.099     10.773      0.000
    MDE18              1.820      0.139     13.073      0.000
    EAT18              0.263      0.036      7.285      0.000
    PTSD18             0.993      0.142      6.999      0.000

 THD      BY
    PSY18              0.123      0.027      4.508      0.000
    PRODA18            0.486      0.065      7.513      0.000
    PRODB18            0.377      0.051      7.453      0.000

 P        BY
    ADHD18             2.628      0.107     24.537      0.000
    ALC18              0.796      0.063     12.575      0.000
    MAR18              0.666      0.081      8.269      0.000
    SMK18              0.728      0.060     12.095      0.000
    CD18               1.511      0.086     17.589      0.000
    GAD18              0.689      0.077      8.914      0.000
    MDE18              1.441      0.100     14.347      0.000
    EAT18              0.314      0.031     10.206      0.000
    PTSD18             1.069      0.134      7.950      0.000
    PSY18              0.066      0.016      4.202      0.000
    PRODA18            0.323      0.034      9.546      0.000
    PRODB18            0.339      0.036      9.538      0.000

 INT      WITH
    THD                0.000      0.000    999.000    999.000
    P                  0.000      0.000    999.000    999.000

 P        WITH
    THD                0.000      0.000    999.000    999.000

 Means
    INT                0.000      0.000    999.000    999.000
    THD                0.000      0.000    999.000    999.000
    P                  0.000      0.000    999.000    999.000

 Intercepts
    SMK18              0.672      0.044     15.379      0.000
    CD18               2.125      0.061     34.837      0.000
    ADHD18             5.789      0.105     54.979      0.000
    PSY18              0.044      0.007      6.313      0.000
    ALC18              1.125      0.042     26.783      0.000
    MAR18              0.335      0.034      9.757      0.000
    GAD18              1.134      0.052     22.005      0.000
    MDE18              1.809      0.072     25.018      0.000
    EAT18              0.452      0.022     20.714      0.000
    PTSD18             1.365      0.082     16.739      0.000
    PRODA18            0.273      0.018     15.100      0.000
    PRODB18            0.247      0.018     13.689      0.000

 Variances
```

```
    INT                1.000      0.000    999.000    999.000
    THD                1.000      0.000    999.000    999.000
    P                  1.000      0.000    999.000    999.000

 Residual Variances
    SMK18              2.098      0.159     13.215      0.000
    CD18               2.927      0.206     14.237      0.000
    ADHD18            11.489      0.519     22.149      0.000
    PSY18              0.074      0.018      4.115      0.000
    ALC18              2.185      0.125     17.541      0.000
    MAR18              1.447      0.152      9.491      0.000
    GAD18              3.020      0.201     15.038      0.000
    MDE18              3.443      0.454      7.585      0.000
    EAT18              0.611      0.043     14.143      0.000
    PTSD18             8.882      0.562     15.798      0.000
    PRODA18            0.248      0.051      4.862      0.000
    PRODB18            0.327      0.042      7.761      0.000

R-SQUARE

    Observed                                        Two-Tailed
    Variable        Estimate       S.E.  Est./S.E.    P-Value

    SMK18              0.202      0.027      7.383      0.000
    CD18               0.438      0.040     11.029      0.000
    ADHD18             0.376      0.027     14.126      0.000
    PSY18              0.208      0.054      3.887      0.000
    ALC18              0.225      0.028      7.942      0.000
    MAR18              0.235      0.035      6.718      0.000
    GAD18              0.347      0.040      8.656      0.000
    MDE18              0.610      0.052     11.717      0.000
    EAT18              0.215      0.026      8.423      0.000
    PTSD18             0.193      0.027      7.153      0.000
    PRODA18            0.579      0.090      6.441      0.000
    PRODB18            0.440      0.062      7.080      0.000

SUMMARY OF FACTOR SCORES

     FACTOR SCORE INFORMATION (COMPLETE-DATA PATTERN)
           FACTOR DETERMINACIES

           INT        0.741
           THD        0.753
           P          0.866

     FACTOR SCORE INFORMATION (PATTERN 2)
           FACTOR DETERMINACIES

           INT        0.738
           THD        0.409
           P          0.855

     FACTOR SCORE INFORMATION (PATTERN 3)
           FACTOR DETERMINACIES

           INT        0.733
           THD        0.752
           P          0.864

     FACTOR SCORE INFORMATION (PATTERN 4)
           FACTOR DETERMINACIES

           INT        0.733
           THD        0.752
           P          0.864

     FACTOR SCORE INFORMATION (PATTERN 5)
           FACTOR DETERMINACIES

           INT        0.591
```

```
           THD        0.752
           P          0.861

     FACTOR SCORE INFORMATION (PATTERN 6)
           FACTOR DETERMINACIES

           INT        0.734
           THD        0.747
           P          0.840

     FACTOR SCORE INFORMATION (PATTERN 7)
           FACTOR DETERMINACIES

           INT        0.731
           THD        0.745
           P          0.830

     FACTOR SCORE INFORMATION (PATTERN 8)
           FACTOR DETERMINACIES

           INT        0.726
           THD        0.741
           P          0.811

     FACTOR SCORE INFORMATION (PATTERN 9)
           FACTOR DETERMINACIES

           INT        0.725
           THD        0.000
           P          0.808

     FACTOR SCORE INFORMATION (PATTERN 10)
           FACTOR DETERMINACIES

           INT        0.546
           THD        0.723
           P          0.723

     FACTOR SCORE INFORMATION (PATTERN 11)
           FACTOR DETERMINACIES

           INT        0.710
           THD        0.000
           P          0.749

     FACTOR SCORE INFORMATION (PATTERN 12)
           FACTOR DETERMINACIES

           INT        0.738
           THD        0.751
           P          0.856

     FACTOR SCORE INFORMATION (PATTERN 13)
           FACTOR DETERMINACIES

           INT        0.735
           THD        0.748
           P          0.843

     Beginning Time:  09:54:46
        Ending Time:  09:54:46
       Elapsed Time:  00:00:00
```

MUTHEN & MUTHEN
3463 Stoner Ave.
Los Angeles, CA 90066
Tel: (310) 391-9971
Fax: (310) 391-8971
Web: www.StatModel.com
Support: Support@StatModel.com Copyright (c) 1998-2021 Muthen & Muthen

```
Mplus VERSION 8.7
MUTHEN & MUTHEN
10/27/2022   9:53 AM

INPUT INSTRUCTIONS

  TITLE:  E-Risk P Compare (Bifactor, -Internalizing);

  DATA:   FILE IS PCompare_Oct2020.dat;

  VARIABLE:
          NAMES ARE
              familyid atwinid rorder sex zygosity iqe5 seswq35 lowsc harm
              polyv512 aces512 famhist smk18 cd18 adhd18 psy18
              polyv18 CRP18 IL6_18 suPAR iq18 fsiq18
              alc18 mar18 gad18 mde18 eat18 ptsd18 prod18 prodA18 prodB18;

          MISSING
              ALL (999999);

          CLUSTER = familyid;

          USEVARIABLES ARE
              smk18 cd18 adhd18 psy18 alc18 mar18 gad18 mde18 eat18 PTSD18
              prodA18 prodB18;

          IDVARIABLE IS
              atwinid;

  ANALYSIS:
      TYPE      = COMPLEX;
      ESTIMATOR = MLR;
      MODEL     = NOCOVARIANCES;

  MODEL:

      ext BY adhd18* alc18 mar18 smk18 cd18;
      thd BY psy18*  prodA18 prodB18;

      p   BY adhd18* alc18 mar18 smk18 cd18 gad18 mde18 eat18 PTSD18 psy18
             prodA18 prodB18;

      [ext@0 thd@0 p@0];
       ext@1 thd@1 p@1;

      ext WITH thd@0;
      P   WITH ext@0 thd@0;

  OUTPUT: SAMPSTAT STANDARDIZED FSDETERMINACY PATTERNS;

  !SAVEDATA:
  !    FILE     = BifactorS-Int.dat;
  !    SAVE     = FSCORES;
  !    MISSFLAG = 9999;

*** WARNING
  Data set contains cases with missing on all variables.
  These cases were not included in the analysis.
  Number of cases with missing on all variables:  166
   1 WARNING(S) FOUND IN THE INPUT INSTRUCTIONS

E-Risk P Compare (Bifactor, -Internalizing);

SUMMARY OF ANALYSIS

Number of groups                                                 1
Number of observations                                        2066

Number of dependent variables                                   12
Number of independent variables                                  0
```

Number of continuous latent variables 3

Observed dependent variables

Continuous

SMK18	CD18	ADHD18	PSY18	ALC18	MAR18
GAD18	MDE18	EAT18	PTSD18	PRODA18	PRODB18

Continuous latent variables

EXT	THD	P

Variables with special functions

Cluster variable	FAMILYID
ID variable	ATWINID

Estimator	MLR
Information matrix	OBSERVED
Maximum number of iterations	1000
Convergence criterion	0.500D-04
Maximum number of steepest descent iterations	20
Maximum number of iterations for H1	2000
Convergence criterion for H1	0.100D-03

Input data file(s)
PCompare_Oct2020.dat

Input data format FREE

SUMMARY OF DATA

Number of missing data patterns	13
Number of clusters	1044

SUMMARY OF MISSING DATA PATTERNS

MISSING DATA PATTERNS (x = not missing)

	1	2	3	4	5	6	7	8	9	10	11	12	13
SMK18	x	x	x	x	x	x	x	x	x	x	x		
CD18	x	x	x	x	x	x						x	x
ADHD18	x	x	x	x	x		x	x	x			x	x
PSY18	x	x	x	x	x	x	x	x		x		x	x
ALC18	x	x	x	x	x	x	x		x		x	x	
MAR18	x	x	x	x	x	x	x	x	x	x	x	x	x
GAD18	x	x	x	x	x	x	x	x	x	x	x	x	x
MDE18	x	x	x	x		x	x	x	x		x	x	x
EAT18	x	x	x		x	x	x	x	x		x	x	x
PTSD18	x	x		x	x	x	x	x	x	x	x	x	x
PRODA18	x		x	x	x	x	x	x		x		x	x
PRODB18	x		x	x	x	x	x	x		x		x	x

MISSING DATA PATTERN FREQUENCIES

Pattern	Frequency	Pattern	Frequency	Pattern	Frequency
1	2040	6	3	11	1
2	1	7	8	12	3
3	2	8	1	13	1
4	1	9	2		
5	2	10	1		

COVARIANCE COVERAGE OF DATA

Minimum covariance coverage value 0.100

PROPORTION OF DATA PRESENT

Covariance Coverage

SMK18	CD18	ADHD18	PSY18	ALC18

	SMK18	CD18	ADHD18	PSY18	ALC18
SMK18	0.998				
CD18	0.992	0.994			
ADHD18	0.996	0.992	0.998		
PSY18	0.997	0.994	0.997	0.999	
ALC18	0.997	0.993	0.997	0.997	0.999
MAR18	0.998	0.994	0.998	0.999	0.999
GAD18	0.998	0.994	0.998	0.999	0.999
MDE18	0.997	0.993	0.997	0.997	0.998
EAT18	0.997	0.993	0.997	0.998	0.998
PTSD18	0.997	0.993	0.997	0.998	0.998
PRODA18	0.996	0.993	0.996	0.998	0.997
PRODB18	0.996	0.993	0.996	0.998	0.997

Covariance Coverage

	MAR18	GAD18	MDE18	EAT18	PTSD18
MAR18	1.000				
GAD18	1.000	1.000			
MDE18	0.999	0.999	0.999		
EAT18	0.999	0.999	0.998	0.999	
PTSD18	0.999	0.999	0.998	0.998	0.999
PRODA18	0.998	0.998	0.997	0.997	0.997
PRODB18	0.998	0.998	0.997	0.997	0.997

Covariance Coverage

	PRODA18	PRODB18
PRODA18	0.998	
PRODB18	0.998	0.998

SAMPLE STATISTICS

ESTIMATED SAMPLE STATISTICS

Means

SMK18	CD18	ADHD18	PSY18	ALC18
0.672	2.123	5.788	0.044	1.125

Means

MAR18	GAD18	MDE18	EAT18	PTSD18
0.335	1.134	1.809	0.453	1.365

Means

PRODA18	PRODB18
0.273	0.247

Covariances

	SMK18	CD18	ADHD18	PSY18	ALC18
SMK18	2.630				
CD18	1.183	5.205			
ADHD18	1.560	4.202	18.393		
PSY18	0.044	0.076	0.167	0.094	
ALC18	0.522	1.417	2.176	0.022	2.818
MAR18	0.839	1.260	1.265	0.048	0.442
GAD18	0.292	0.672	2.373	0.109	0.471
MDE18	0.933	1.595	4.348	0.153	1.282
EAT18	0.212	0.341	1.041	0.036	0.299
PTSD18	0.938	1.135	2.751	0.158	0.660
PRODA18	0.181	0.372	0.881	0.081	0.134
PRODB18	0.150	0.437	0.892	0.069	0.217

Covariances

	MAR18	GAD18	MDE18	EAT18	PTSD18
MAR18	1.891				
GAD18	0.241	4.627			

MDE18	0.689	2.941	8.835		
EAT18	0.107	0.505	0.917	0.779	
PTSD18	0.366	1.711	3.372	0.637	11.012
PRODA18	0.201	0.488	0.772	0.177	0.882
PRODB18	0.233	0.489	0.761	0.141	0.690

Covariances

	PRODA18	PRODB18
PRODA18	0.589	
PRODB18	0.293	0.584

Correlations

	SMK18	CD18	ADHD18	PSY18	ALC18
SMK18	1.000				
CD18	0.320	1.000			
ADHD18	0.224	0.429	1.000		
PSY18	0.089	0.109	0.127	1.000	
ALC18	0.192	0.370	0.302	0.044	1.000
MAR18	0.376	0.402	0.215	0.114	0.192
GAD18	0.084	0.137	0.257	0.166	0.131
MDE18	0.194	0.235	0.341	0.168	0.257
EAT18	0.148	0.169	0.275	0.135	0.202
PTSD18	0.174	0.150	0.193	0.156	0.118
PRODA18	0.145	0.212	0.268	0.346	0.104
PRODB18	0.121	0.251	0.272	0.294	0.169

Correlations

	MAR18	GAD18	MDE18	EAT18	PTSD18
MAR18	1.000				
GAD18	0.081	1.000			
MDE18	0.169	0.460	1.000		
EAT18	0.088	0.266	0.350	1.000	
PTSD18	0.080	0.240	0.342	0.218	1.000
PRODA18	0.191	0.296	0.339	0.262	0.346
PRODB18	0.222	0.298	0.335	0.210	0.272

Correlations

	PRODA18	PRODB18
PRODA18	1.000	
PRODB18	0.500	1.000

MAXIMUM LOG-LIKELIHOOD VALUE FOR THE UNRESTRICTED (H1) MODEL IS -42633.558

UNIVARIATE SAMPLE STATISTICS

UNIVARIATE HIGHER-ORDER MOMENT DESCRIPTIVE STATISTICS

Variable/ Sample Size	Mean/ Variance	Skewness/ Kurtosis	Minimum/ Maximum	% with Min/Max	Percentiles 20%/60%	40%/80%	Median
SMK18	0.670	2.585	0.000	80.94%	0.000	0.000	0.000
2062.000	2.625	6.106	10.000	0.05%	0.000	0.000	
CD18	2.121	1.262	0.000	30.54%	0.000	1.000	1.000
2053.000	5.209	1.205	11.000	0.24%	2.000	4.000	
ADHD18	5.787	0.510	0.000	10.92%	2.000	4.000	5.000
2061.000	18.399	-0.528	18.000	0.44%	7.000	10.000	
PSY18	0.044	9.848	0.000	97.14%	0.000	0.000	0.000
2063.000	0.094	125.741	6.000	0.05%	0.000	0.000	
ALC18	1.125	1.949	0.000	53.76%	0.000	0.000	0.000
2063.000	2.818	4.436	11.000	0.10%	1.000	2.000	
MAR18	0.335	4.751	0.000	92.40%	0.000	0.000	0.000
2066.000	1.891	23.548	10.000	0.44%	0.000	0.000	
GAD18	1.134	1.593	0.000	75.46%	0.000	0.000	0.000
2066.000	4.627	0.931	7.000	3.05%	0.000	3.000	
MDE18	1.806	1.273	0.000	68.93%	0.000	0.000	0.000
2063.000	8.828	-0.017	9.000	3.34%	0.000	5.000	

```
     EAT18                0.453       2.416       0.000   71.71%       0.000      0.000      0.000
            2064.000      0.779       6.256       5.000    0.44%       0.000      1.000
     PTSD18               1.365       2.642       0.000   79.41%       0.000      0.000      0.000
            2064.000     11.014       6.296      17.000    0.24%       0.000      1.000
     PRODA18              0.273       3.450       0.000   85.55%       0.000      0.000      0.000
            2062.000      0.588      14.002       6.000    0.19%       0.000      0.000
     PRODB18              0.247       3.734       0.000   87.83%       0.000      0.000      0.000
            2062.000      0.584      15.720       6.000    0.19%       0.000      0.000

THE MODEL ESTIMATION TERMINATED NORMALLY

MODEL FIT INFORMATION

Number of Free Parameters                       44

Loglikelihood

          H0 Value                      -42800.304
          H0 Scaling Correction Factor      4.2348
            for MLR
          H1 Value                      -42633.558
          H1 Scaling Correction Factor      3.0107
            for MLR

Information Criteria

          Akaike (AIC)                   85688.608
          Bayesian (BIC)                 85936.476
          Sample-Size Adjusted BIC       85796.684
            (n* = (n + 2) / 24)

Chi-Square Test of Model Fit

          Value                            181.255*
          Degrees of Freedom                    46
          P-Value                           0.0000
          Scaling Correction Factor         1.8399
            for MLR

*   The chi-square value for MLM, MLMV, MLR, ULSMV, WLSM and WLSMV cannot be used
    for chi-square difference testing in the regular way.  MLM, MLR and WLSM
    chi-square difference testing is described on the Mplus website.  MLMV, WLSMV,
    and ULSMV difference testing is done using the DIFFTEST option.

RMSEA (Root Mean Square Error Of Approximation)

          Estimate                           0.038
          90 Percent C.I.                    0.032  0.044
          Probability RMSEA <= .05           1.000

CFI/TLI

          CFI                                0.943
          TLI                                0.918

Chi-Square Test of Model Fit for the Baseline Model

          Value                           2418.883
          Degrees of Freedom                    66
          P-Value                           0.0000

SRMR (Standardized Root Mean Square Residual)

          Value                              0.035


MODEL RESULTS
                                                    Two-Tailed
                    Estimate       S.E.  Est./S.E.    P-Value
```

```
EXT      BY
   ADHD18             1.489      0.126     11.834      0.000
   ALC18              0.597      0.062      9.598      0.000
   MAR18              0.657      0.088      7.424      0.000
   SMK18              0.630      0.087      7.261      0.000
   CD18               1.583      0.108     14.641      0.000

THD      BY
   PSY18              0.115      0.026      4.391      0.000
   PRODA18            0.423      0.066      6.379      0.000
   PRODB18            0.329      0.055      5.991      0.000

P        BY
   ADHD18             2.066      0.109     18.870      0.000
   ALC18              0.525      0.062      8.424      0.000
   MAR18              0.312      0.062      5.031      0.000
   SMK18              0.407      0.061      6.644      0.000
   CD18               0.773      0.076     10.201      0.000
   GAD18              1.237      0.065     18.967      0.000
   MDE18              2.152      0.082     26.304      0.000
   EAT18              0.428      0.032     13.339      0.000
   PTSD18             1.588      0.142     11.154      0.000
   PSY18              0.081      0.017      4.844      0.000
   PRODA18            0.402      0.034     11.695      0.000
   PRODB18            0.382      0.035     10.954      0.000

EXT      WITH
   THD                0.000      0.000    999.000    999.000
   P                  0.000      0.000    999.000    999.000

P        WITH
   THD                0.000      0.000    999.000    999.000

Means
   EXT                0.000      0.000    999.000    999.000
   THD                0.000      0.000    999.000    999.000
   P                  0.000      0.000    999.000    999.000

Intercepts
   SMK18              0.672      0.044     15.381      0.000
   CD18               2.123      0.061     34.828      0.000
   ADHD18             5.788      0.105     54.973      0.000
   PSY18              0.044      0.007      6.313      0.000
   ALC18              1.125      0.042     26.782      0.000
   MAR18              0.335      0.034      9.757      0.000
   GAD18              1.134      0.052     22.005      0.000
   MDE18              1.810      0.072     25.030      0.000
   EAT18              0.453      0.022     20.713      0.000
   PTSD18             1.365      0.082     16.737      0.000
   PRODA18            0.273      0.018     15.101      0.000
   PRODB18            0.247      0.018     13.689      0.000

Variances
   EXT                1.000      0.000    999.000    999.000
   THD                1.000      0.000    999.000    999.000
   P                  1.000      0.000    999.000    999.000

Residual Variances
   SMK18              2.065      0.174     11.870      0.000
   CD18               2.103      0.301      6.982      0.000
   ADHD18            11.909      0.453     26.278      0.000
   PSY18              0.074      0.018      4.140      0.000
   ALC18              2.187      0.125     17.552      0.000
   MAR18              1.362      0.147      9.291      0.000
   GAD18              3.096      0.161     19.288      0.000
   MDE18              4.206      0.283     14.842      0.000
   EAT18              0.595      0.043     13.971      0.000
   PTSD18             8.490      0.533     15.917      0.000
   PRODA18            0.248      0.048      5.157      0.000
   PRODB18            0.330      0.041      7.973      0.000
```

```
QUALITY OF NUMERICAL RESULTS

     Condition Number for the Information Matrix              0.174E-03
       (ratio of smallest to largest eigenvalue)

STANDARDIZED MODEL RESULTS

STDYX Standardization
                                                           Two-Tailed
                    Estimate       S.E.  Est./S.E.    P-Value

 EXT      BY
    ADHD18             0.347      0.028     12.275      0.000
    ALC18              0.356      0.034     10.503      0.000
    MAR18              0.478      0.047     10.088      0.000
    SMK18              0.389      0.052      7.500      0.000
    CD18               0.694      0.044     15.837      0.000

 THD      BY
    PSY18              0.376      0.063      5.980      0.000
    PRODA18            0.551      0.078      7.068      0.000
    PRODB18            0.431      0.068      6.306      0.000

 P        BY
    ADHD18             0.482      0.024     20.199      0.000
    ALC18              0.313      0.033      9.471      0.000
    MAR18              0.227      0.040      5.747      0.000
    SMK18              0.251      0.035      7.185      0.000
    CD18               0.339      0.031     10.946      0.000
    GAD18              0.575      0.025     22.778      0.000
    MDE18              0.724      0.022     32.979      0.000
    EAT18              0.485      0.028     17.119      0.000
    PTSD18             0.479      0.033     14.696      0.000
    PSY18              0.263      0.031      8.404      0.000
    PRODA18            0.525      0.031     16.791      0.000
    PRODB18            0.500      0.031     16.163      0.000

 EXT      WITH
    THD                0.000      0.000    999.000    999.000
    P                  0.000      0.000    999.000    999.000

 P        WITH
    THD                0.000      0.000    999.000    999.000

 Means
    EXT                0.000      0.000    999.000    999.000
    THD                0.000      0.000    999.000    999.000
    P                  0.000      0.000    999.000    999.000

 Intercepts
    SMK18              0.414      0.014     29.283      0.000
    CD18               0.931      0.018     51.084      0.000
    ADHD18             1.350      0.023     57.496      0.000
    PSY18              0.144      0.011     12.741      0.000
    ALC18              0.670      0.015     43.282      0.000
    MAR18              0.244      0.012     20.944      0.000
    GAD18              0.527      0.014     36.592      0.000
    MDE18              0.609      0.015     39.369      0.000
    EAT18              0.513      0.013     39.853      0.000
    PTSD18             0.411      0.012     33.051      0.000
    PRODA18            0.356      0.012     29.061      0.000
    PRODB18            0.323      0.012     27.104      0.000

 Variances
    EXT                1.000      0.000    999.000    999.000
    THD                1.000      0.000    999.000    999.000
    P                  1.000      0.000    999.000    999.000

 Residual Variances
    SMK18              0.786      0.036     21.656      0.000
```

```
    CD18               0.404      0.057      7.042      0.000
    ADHD18             0.647      0.022     29.228      0.000
    PSY18              0.789      0.054     14.532      0.000
    ALC18              0.776      0.027     28.335      0.000
    MAR18              0.720      0.042     17.296      0.000
    GAD18              0.669      0.029     23.029      0.000
    MDE18              0.476      0.032     14.977      0.000
    EAT18              0.764      0.028     27.763      0.000
    PTSD18             0.771      0.031     24.735      0.000
    PRODA18            0.421      0.085      4.939      0.000
    PRODB18            0.565      0.060      9.419      0.000

STDY Standardization
                                                    Two-Tailed
                    Estimate       S.E.  Est./S.E.    P-Value

 EXT      BY
    ADHD18             0.347      0.028     12.275      0.000
    ALC18              0.356      0.034     10.503      0.000
    MAR18              0.478      0.047     10.088      0.000
    SMK18              0.389      0.052      7.500      0.000
    CD18               0.694      0.044     15.837      0.000

 THD      BY
    PSY18              0.376      0.063      5.980      0.000
    PRODA18            0.551      0.078      7.068      0.000
    PRODB18            0.431      0.068      6.306      0.000

 P        BY
    ADHD18             0.482      0.024     20.199      0.000
    ALC18              0.313      0.033      9.471      0.000
    MAR18              0.227      0.040      5.747      0.000
    SMK18              0.251      0.035      7.185      0.000
    CD18               0.339      0.031     10.946      0.000
    GAD18              0.575      0.025     22.778      0.000
    MDE18              0.724      0.022     32.979      0.000
    EAT18              0.485      0.028     17.119      0.000
    PTSD18             0.479      0.033     14.696      0.000
    PSY18              0.263      0.031      8.404      0.000
    PRODA18            0.525      0.031     16.791      0.000
    PRODB18            0.500      0.031     16.163      0.000

 EXT      WITH
    THD                0.000      0.000    999.000    999.000
    P                  0.000      0.000    999.000    999.000

 P        WITH
    THD                0.000      0.000    999.000    999.000

 Means
    EXT                0.000      0.000    999.000    999.000
    THD                0.000      0.000    999.000    999.000
    P                  0.000      0.000    999.000    999.000

 Intercepts
    SMK18              0.414      0.014     29.283      0.000
    CD18               0.931      0.018     51.084      0.000
    ADHD18             1.350      0.023     57.496      0.000
    PSY18              0.144      0.011     12.741      0.000
    ALC18              0.670      0.015     43.282      0.000
    MAR18              0.244      0.012     20.944      0.000
    GAD18              0.527      0.014     36.592      0.000
    MDE18              0.609      0.015     39.369      0.000
    EAT18              0.513      0.013     39.853      0.000
    PTSD18             0.411      0.012     33.051      0.000
    PRODA18            0.356      0.012     29.061      0.000
    PRODB18            0.323      0.012     27.104      0.000

 Variances
    EXT                1.000      0.000    999.000    999.000
```

```
   THD                1.000      0.000    999.000    999.000
   P                  1.000      0.000    999.000    999.000

 Residual Variances
   SMK18              0.786      0.036     21.656      0.000
   CD18               0.404      0.057      7.042      0.000
   ADHD18             0.647      0.022     29.228      0.000
   PSY18              0.789      0.054     14.532      0.000
   ALC18              0.776      0.027     28.335      0.000
   MAR18              0.720      0.042     17.296      0.000
   GAD18              0.669      0.029     23.029      0.000
   MDE18              0.476      0.032     14.977      0.000
   EAT18              0.764      0.028     27.763      0.000
   PTSD18             0.771      0.031     24.735      0.000
   PRODA18            0.421      0.085      4.939      0.000
   PRODB18            0.565      0.060      9.419      0.000

STD Standardization

                                                  Two-Tailed
                    Estimate       S.E.  Est./S.E.    P-Value

 EXT      BY
   ADHD18             1.489      0.126     11.834      0.000
   ALC18              0.597      0.062      9.598      0.000
   MAR18              0.657      0.088      7.424      0.000
   SMK18              0.630      0.087      7.261      0.000
   CD18               1.583      0.108     14.641      0.000

 THD      BY
   PSY18              0.115      0.026      4.391      0.000
   PRODA18            0.423      0.066      6.379      0.000
   PRODB18            0.329      0.055      5.991      0.000

 P        BY
   ADHD18             2.066      0.109     18.870      0.000
   ALC18              0.525      0.062      8.424      0.000
   MAR18              0.312      0.062      5.031      0.000
   SMK18              0.407      0.061      6.644      0.000
   CD18               0.773      0.076     10.201      0.000
   GAD18              1.237      0.065     18.967      0.000
   MDE18              2.152      0.082     26.304      0.000
   EAT18              0.428      0.032     13.339      0.000
   PTSD18             1.588      0.142     11.154      0.000
   PSY18              0.081      0.017      4.844      0.000
   PRODA18            0.402      0.034     11.695      0.000
   PRODB18            0.382      0.035     10.954      0.000

 EXT      WITH
   THD                0.000      0.000    999.000    999.000
   P                  0.000      0.000    999.000    999.000

 P        WITH
   THD                0.000      0.000    999.000    999.000

 Means
   EXT                0.000      0.000    999.000    999.000
   THD                0.000      0.000    999.000    999.000
   P                  0.000      0.000    999.000    999.000

 Intercepts
   SMK18              0.672      0.044     15.381      0.000
   CD18               2.123      0.061     34.828      0.000
   ADHD18             5.788      0.105     54.973      0.000
   PSY18              0.044      0.007      6.313      0.000
   ALC18              1.125      0.042     26.782      0.000
   MAR18              0.335      0.034      9.757      0.000
   GAD18              1.134      0.052     22.005      0.000
   MDE18              1.810      0.072     25.030      0.000
   EAT18              0.453      0.022     20.713      0.000
   PTSD18             1.365      0.082     16.737      0.000
```

PRODA18	0.273	0.018	15.101	0.000
PRODB18	0.247	0.018	13.689	0.000

Variances

EXT	1.000	0.000	999.000	999.000
THD	1.000	0.000	999.000	999.000
P	1.000	0.000	999.000	999.000

Residual Variances

SMK18	2.065	0.174	11.870	0.000
CD18	2.103	0.301	6.982	0.000
ADHD18	11.909	0.453	26.278	0.000
PSY18	0.074	0.018	4.140	0.000
ALC18	2.187	0.125	17.552	0.000
MAR18	1.362	0.147	9.291	0.000
GAD18	3.096	0.161	19.288	0.000
MDE18	4.206	0.283	14.842	0.000
EAT18	0.595	0.043	13.971	0.000
PTSD18	8.490	0.533	15.917	0.000
PRODA18	0.248	0.048	5.157	0.000
PRODB18	0.330	0.041	7.973	0.000

R-SQUARE

Observed Variable	Estimate	S.E.	Est./S.E.	Two-Tailed P-Value
SMK18	0.214	0.036	5.907	0.000
CD18	0.596	0.057	10.387	0.000
ADHD18	0.353	0.022	15.915	0.000
PSY18	0.211	0.054	3.884	0.000
ALC18	0.224	0.027	8.184	0.000
MAR18	0.280	0.042	6.717	0.000
GAD18	0.331	0.029	11.389	0.000
MDE18	0.524	0.032	16.489	0.000
EAT18	0.236	0.028	8.560	0.000
PTSD18	0.229	0.031	7.348	0.000
PRODA18	0.579	0.085	6.784	0.000
PRODB18	0.435	0.060	7.262	0.000

SUMMARY OF FACTOR SCORES

FACTOR SCORE INFORMATION (COMPLETE-DATA PATTERN)
FACTOR DETERMINACIES

EXT	0.794
THD	0.694
P	0.871

FACTOR SCORE INFORMATION (PATTERN 2)
FACTOR DETERMINACIES

EXT	0.791
THD	0.386
P	0.853

FACTOR SCORE INFORMATION (PATTERN 3)
FACTOR DETERMINACIES

EXT	0.792
THD	0.691
P	0.860

FACTOR SCORE INFORMATION (PATTERN 4)
FACTOR DETERMINACIES

EXT	0.792
THD	0.690
P	0.860

```
     FACTOR SCORE INFORMATION (PATTERN 5)
           FACTOR DETERMINACIES

           EXT        0.785
           THD        0.676
           P          0.820

     FACTOR SCORE INFORMATION (PATTERN 6)
           FACTOR DETERMINACIES

           EXT        0.787
           THD        0.692
           P          0.865

     FACTOR SCORE INFORMATION (PATTERN 7)
           FACTOR DETERMINACIES

           EXT        0.656
           THD        0.694
           P          0.871

     FACTOR SCORE INFORMATION (PATTERN 8)
           FACTOR DETERMINACIES

           EXT        0.621
           THD        0.694
           P          0.869

     FACTOR SCORE INFORMATION (PATTERN 9)
           FACTOR DETERMINACIES

           EXT        0.652
           THD        0.000
           P          0.849

     FACTOR SCORE INFORMATION (PATTERN 10)
           FACTOR DETERMINACIES

           EXT        0.569
           THD        0.659
           P          0.768

     FACTOR SCORE INFORMATION (PATTERN 11)
           FACTOR DETERMINACIES

           EXT        0.619
           THD        0.000
           P          0.839

     FACTOR SCORE INFORMATION (PATTERN 12)
           FACTOR DETERMINACIES

           EXT        0.782
           THD        0.694
           P          0.871

     FACTOR SCORE INFORMATION (PATTERN 13)
           FACTOR DETERMINACIES

           EXT        0.772
           THD        0.694
           P          0.869

     Beginning Time:  09:53:04
        Ending Time:  09:53:05
       Elapsed Time:  00:00:01

MUTHEN & MUTHEN
3463 Stoner Ave.
Los Angeles, CA  90066
```

```
Mplus VERSION 8.7
MUTHEN & MUTHEN
10/27/2022  10:07 AM

INPUT INSTRUCTIONS

  TITLE:  E-Risk P Compare (Bifactor - Thought Disorders);

  DATA:   FILE IS PCompare_Oct2020.dat;

  VARIABLE:
          NAMES ARE
              familyid atwinid rorder sex zygosity iqe5 seswq35 lowsc harm
              polyv512 aces512 famhist smk18 cd18 adhd18 psy18
              polyv18 CRP18 IL6_18 suPAR iq18 fsiq18
              alc18 mar18 gad18 mde18 eat18 ptsd18 prod18 prodA18 prodB18;

          MISSING
              ALL (999999);

          CLUSTER = familyid;

          USEVARIABLES ARE
              smk18 cd18 adhd18 psy18 alc18 mar18 gad18 mde18 eat18 PTSD18
              prodA18 prodB18;

          IDVARIABLE IS
              atwinid;

  ANALYSIS:
      TYPE      = COMPLEX;
      ESTIMATOR = MLR;
      MODEL     = NOCOVARIANCES;

  MODEL:

      ext BY adhd18* alc18 mar18 smk18 cd18;
      int BY gad18*  mde18 eat18 PTSD18;

      p   BY adhd18* alc18 mar18 smk18 cd18 gad18 mde18 eat18 PTSD18 psy18
             prodA18 prodB18;

      [ext@0 int@0 p@0];
       ext@1 int@1 p@1;

      ext WITH int@0;
      P   WITH ext@0 int@0;

  OUTPUT: SAMPSTAT STANDARDIZED FSDETERMINACY PATTERNS;

  !SAVEDATA:
  !    FILE     = BifactorS-ThD.dat;
  !    SAVE     = FSCORES;
  !    MISSFLAG = 9999;

*** WARNING
  Data set contains cases with missing on all variables.
  These cases were not included in the analysis.
  Number of cases with missing on all variables:  166
   1 WARNING(S) FOUND IN THE INPUT INSTRUCTIONS

E-Risk P Compare (Bifactor - Thought Disorders);

SUMMARY OF ANALYSIS

Number of groups                                                 1
Number of observations                                        2066

Number of dependent variables                                   12
```

```
Number of independent variables                                 0
Number of continuous latent variables                           3

Observed dependent variables

  Continuous
   SMK18       CD18        ADHD18      PSY18       ALC18       MAR18
   GAD18       MDE18       EAT18       PTSD18      PRODA18     PRODB18

Continuous latent variables
   EXT         INT         P

Variables with special functions

  Cluster variable      FAMILYID
  ID variable           ATWINID

Estimator                                                     MLR
Information matrix                                       OBSERVED
Maximum number of iterations                                 1000
Convergence criterion                                   0.500D-04
Maximum number of steepest descent iterations                  20
Maximum number of iterations for H1                          2000
Convergence criterion for H1                            0.100D-03

Input data file(s)
  PCompare_Oct2020.dat

Input data format  FREE

SUMMARY OF DATA

     Number of missing data patterns            13
     Number of clusters                       1044

SUMMARY OF MISSING DATA PATTERNS

     MISSING DATA PATTERNS (x = not missing)

           1  2  3  4  5  6  7  8  9 10 11 12 13
 SMK18     x  x  x  x  x  x  x  x  x  x  x
 CD18      x  x  x  x  x  x                 x  x
 ADHD18    x  x  x  x  x     x  x  x        x  x
 PSY18     x  x  x  x  x  x  x  x     x     x  x
 ALC18     x  x  x  x  x  x  x     x     x  x
 MAR18     x  x  x  x  x  x  x  x  x  x  x  x  x
 GAD18     x  x  x  x  x  x  x  x  x  x  x  x  x
 MDE18     x  x  x  x     x  x  x  x     x  x  x
 EAT18     x  x  x     x  x  x  x  x     x  x  x
 PTSD18    x  x     x  x  x  x  x  x  x  x  x  x
 PRODA18   x     x  x  x  x  x  x     x     x  x
 PRODB18   x     x  x  x  x  x  x     x     x  x

     MISSING DATA PATTERN FREQUENCIES

    Pattern   Frequency     Pattern   Frequency     Pattern   Frequency
          1        2040           6           3          11           1
          2           1           7           8          12           3
          3           2           8           1          13           1
          4           1           9           2
          5           2          10           1

COVARIANCE COVERAGE OF DATA

Minimum covariance coverage value   0.100

     PROPORTION OF DATA PRESENT

           Covariance Coverage
              SMK18         CD18          ADHD18        PSY18         ALC18
```

SMK18	0.998				
CD18	0.992	0.994			
ADHD18	0.996	0.992	0.998		
PSY18	0.997	0.994	0.997	0.999	
ALC18	0.997	0.993	0.997	0.997	0.999
MAR18	0.998	0.994	0.998	0.999	0.999
GAD18	0.998	0.994	0.998	0.999	0.999
MDE18	0.997	0.993	0.997	0.997	0.998
EAT18	0.997	0.993	0.997	0.998	0.998
PTSD18	0.997	0.993	0.997	0.998	0.998
PRODA18	0.996	0.993	0.996	0.998	0.997
PRODB18	0.996	0.993	0.996	0.998	0.997

Covariance Coverage

	MAR18	GAD18	MDE18	EAT18	PTSD18
MAR18	1.000				
GAD18	1.000	1.000			
MDE18	0.999	0.999	0.999		
EAT18	0.999	0.999	0.998	0.999	
PTSD18	0.999	0.999	0.998	0.998	0.999
PRODA18	0.998	0.998	0.997	0.997	0.997
PRODB18	0.998	0.998	0.997	0.997	0.997

Covariance Coverage

	PRODA18	PRODB18
PRODA18	0.998	
PRODB18	0.998	0.998

SAMPLE STATISTICS

ESTIMATED SAMPLE STATISTICS

Means

SMK18	CD18	ADHD18	PSY18	ALC18
0.672	2.123	5.788	0.044	1.125

Means

MAR18	GAD18	MDE18	EAT18	PTSD18
0.335	1.134	1.809	0.453	1.365

Means

PRODA18	PRODB18
0.273	0.247

Covariances

	SMK18	CD18	ADHD18	PSY18	ALC18
SMK18	2.630				
CD18	1.183	5.205			
ADHD18	1.560	4.202	18.393		
PSY18	0.044	0.076	0.167	0.094	
ALC18	0.522	1.417	2.176	0.022	2.818
MAR18	0.839	1.260	1.265	0.048	0.442
GAD18	0.292	0.672	2.373	0.109	0.471
MDE18	0.933	1.595	4.348	0.153	1.282
EAT18	0.212	0.341	1.041	0.036	0.299
PTSD18	0.938	1.135	2.751	0.158	0.660
PRODA18	0.181	0.372	0.881	0.081	0.134
PRODB18	0.150	0.437	0.892	0.069	0.217

Covariances

MAR18	GAD18	MDE18	EAT18	PTSD18

MAR18	1.891				
GAD18	0.241	4.627			
MDE18	0.689	2.941	8.835		
EAT18	0.107	0.505	0.917	0.779	
PTSD18	0.366	1.711	3.372	0.637	11.012
PRODA18	0.201	0.488	0.772	0.177	0.882
PRODB18	0.233	0.489	0.761	0.141	0.690

Covariances

	PRODA18	PRODB18
PRODA18	0.589	
PRODB18	0.293	0.584

Correlations

	SMK18	CD18	ADHD18	PSY18	ALC18
SMK18	1.000				
CD18	0.320	1.000			
ADHD18	0.224	0.429	1.000		
PSY18	0.089	0.109	0.127	1.000	
ALC18	0.192	0.370	0.302	0.044	1.000
MAR18	0.376	0.402	0.215	0.114	0.192
GAD18	0.084	0.137	0.257	0.166	0.131
MDE18	0.194	0.235	0.341	0.168	0.257
EAT18	0.148	0.169	0.275	0.135	0.202
PTSD18	0.174	0.150	0.193	0.156	0.118
PRODA18	0.145	0.212	0.268	0.346	0.104
PRODB18	0.121	0.251	0.272	0.294	0.169

Correlations

	MAR18	GAD18	MDE18	EAT18	PTSD18
MAR18	1.000				
GAD18	0.081	1.000			
MDE18	0.169	0.460	1.000		
EAT18	0.088	0.266	0.350	1.000	
PTSD18	0.080	0.240	0.342	0.218	1.000
PRODA18	0.191	0.296	0.339	0.262	0.346
PRODB18	0.222	0.298	0.335	0.210	0.272

Correlations

	PRODA18	PRODB18
PRODA18	1.000	
PRODB18	0.500	1.000

MAXIMUM LOG-LIKELIHOOD VALUE FOR THE UNRESTRICTED (H1) MODEL IS -42633.558

UNIVARIATE SAMPLE STATISTICS

UNIVARIATE HIGHER-ORDER MOMENT DESCRIPTIVE STATISTICS

Variable/ Sample Size	Mean/ Variance	Skewness/ Kurtosis	Minimum/ Maximum	% with Min/Max	Percentiles 20%/60%	40%/80%	Median
SMK18	0.670	2.585	0.000	80.94%	0.000	0.000	0.000
2062.000	2.625	6.106	10.000	0.05%	0.000	0.000	
CD18	2.121	1.262	0.000	30.54%	0.000	1.000	1.000
2053.000	5.209	1.205	11.000	0.24%	2.000	4.000	
ADHD18	5.787	0.510	0.000	10.92%	2.000	4.000	5.000
2061.000	18.399	-0.528	18.000	0.44%	7.000	10.000	
PSY18	0.044	9.848	0.000	97.14%	0.000	0.000	0.000
2063.000	0.094	125.741	6.000	0.05%	0.000	0.000	
ALC18	1.125	1.949	0.000	53.76%	0.000	0.000	0.000
2063.000	2.818	4.436	11.000	0.10%	1.000	2.000	
MAR18	0.335	4.751	0.000	92.40%	0.000	0.000	0.000
2066.000	1.891	23.548	10.000	0.44%	0.000	0.000	
GAD18	1.134	1.593	0.000	75.46%	0.000	0.000	0.000

```
          2066.000       4.627       0.931       7.000    3.05%       0.000      3.000
     MDE18               1.806       1.273       0.000   68.93%       0.000      0.000      0.000
          2063.000       8.828      -0.017       9.000    3.34%       0.000      5.000
     EAT18               0.453       2.416       0.000   71.71%       0.000      0.000      0.000
          2064.000       0.779       6.256       5.000    0.44%       0.000      1.000
     PTSD18              1.365       2.642       0.000   79.41%       0.000      0.000      0.000
          2064.000      11.014       6.296      17.000    0.24%       0.000      1.000
     PRODA18             0.273       3.450       0.000   85.55%       0.000      0.000      0.000
          2062.000       0.588      14.002       6.000    0.19%       0.000      0.000
     PRODB18             0.247       3.734       0.000   87.83%       0.000      0.000      0.000
          2062.000       0.584      15.720       6.000    0.19%       0.000      0.000

THE MODEL ESTIMATION TERMINATED NORMALLY

MODEL FIT INFORMATION

Number of Free Parameters                       45

Loglikelihood

          H0 Value                      -42830.340
          H0 Scaling Correction Factor      4.1301
            for MLR
          H1 Value                      -42633.558
          H1 Scaling Correction Factor      3.0107
            for MLR

Information Criteria

          Akaike (AIC)                   85750.681
          Bayesian (BIC)                 86004.182
          Sample-Size Adjusted BIC       85861.213
            (n* = (n + 2) / 24)

Chi-Square Test of Model Fit

          Value                            208.092*
          Degrees of Freedom                    45
          P-Value                           0.0000
          Scaling Correction Factor         1.8913
            for MLR

*   The chi-square value for MLM, MLMV, MLR, ULSMV, WLSM and WLSMV cannot be used
    for chi-square difference testing in the regular way.  MLM, MLR and WLSM
    chi-square difference testing is described on the Mplus website.  MLMV, WLSMV,
    and ULSMV difference testing is done using the DIFFTEST option.

RMSEA (Root Mean Square Error Of Approximation)

          Estimate                           0.042
          90 Percent C.I.                    0.036  0.048
          Probability RMSEA <= .05           0.989

CFI/TLI

          CFI                                0.931
          TLI                                0.898

Chi-Square Test of Model Fit for the Baseline Model

          Value                           2418.883
          Degrees of Freedom                    66
          P-Value                           0.0000

SRMR (Standardized Root Mean Square Residual)

          Value                              0.041


MODEL RESULTS
```

	Estimate	S.E.	Est./S.E.	Two-Tailed P-Value
EXT BY				
ADHD18	1.632	0.128	12.719	0.000
ALC18	0.674	0.062	10.818	0.000
MAR18	0.616	0.082	7.481	0.000
SMK18	0.645	0.080	8.038	0.000
CD18	1.576	0.095	16.573	0.000
INT BY				
GAD18	0.811	0.118	6.869	0.000
MDE18	1.879	0.212	8.861	0.000
EAT18	0.225	0.043	5.283	0.000
PTSD18	0.586	0.134	4.358	0.000
P BY				
ADHD18	1.843	0.122	15.166	0.000
ALC18	0.402	0.069	5.869	0.000
MAR18	0.373	0.074	5.060	0.000
SMK18	0.373	0.063	5.883	0.000
CD18	0.779	0.081	9.638	0.000
GAD18	0.927	0.072	12.816	0.000
MDE18	1.526	0.096	15.871	0.000
EAT18	0.330	0.036	9.209	0.000
PTSD18	1.463	0.158	9.276	0.000
PSY18	0.128	0.025	5.048	0.000
PRODA18	0.552	0.040	13.840	0.000
PRODB18	0.515	0.038	13.482	0.000
EXT WITH				
INT	0.000	0.000	999.000	999.000
P	0.000	0.000	999.000	999.000
P WITH				
INT	0.000	0.000	999.000	999.000
Means				
EXT	0.000	0.000	999.000	999.000
INT	0.000	0.000	999.000	999.000
P	0.000	0.000	999.000	999.000
Intercepts				
SMK18	0.672	0.044	15.380	0.000
CD18	2.124	0.061	34.831	0.000
ADHD18	5.789	0.105	54.983	0.000
PSY18	0.044	0.007	6.312	0.000
ALC18	1.125	0.042	26.781	0.000
MAR18	0.335	0.034	9.757	0.000
GAD18	1.134	0.052	22.005	0.000
MDE18	1.810	0.072	25.031	0.000
EAT18	0.453	0.022	20.714	0.000
PTSD18	1.365	0.082	16.737	0.000
PRODA18	0.273	0.018	15.100	0.000
PRODB18	0.247	0.018	13.690	0.000
Variances				
EXT	1.000	0.000	999.000	999.000
INT	1.000	0.000	999.000	999.000
P	1.000	0.000	999.000	999.000
Residual Variances				
SMK18	2.073	0.170	12.176	0.000
CD18	2.117	0.256	8.284	0.000
ADHD18	12.337	0.464	26.594	0.000
PSY18	0.077	0.018	4.194	0.000
ALC18	2.202	0.129	17.042	0.000
MAR18	1.372	0.144	9.558	0.000
GAD18	3.111	0.192	16.190	0.000
MDE18	2.974	0.750	3.963	0.000

EAT18	0.619	0.044	13.961	0.000
PTSD18	8.528	0.536	15.901	0.000
PRODA18	0.283	0.029	9.788	0.000
PRODB18	0.319	0.031	10.150	0.000

QUALITY OF NUMERICAL RESULTS

Condition Number for the Information Matrix 0.378E-04
(ratio of smallest to largest eigenvalue)

STANDARDIZED MODEL RESULTS

STDYX Standardization

	Estimate	S.E.	Est./S.E.	Two-Tailed P-Value
EXT BY				
ADHD18	0.380	0.029	13.225	0.000
ALC18	0.402	0.033	12.015	0.000
MAR18	0.448	0.044	10.184	0.000
SMK18	0.398	0.047	8.432	0.000
CD18	0.691	0.038	18.370	0.000
INT BY				
GAD18	0.377	0.054	7.042	0.000
MDE18	0.632	0.070	9.053	0.000
EAT18	0.255	0.046	5.518	0.000
PTSD18	0.177	0.040	4.411	0.000
P BY				
ADHD18	0.430	0.027	15.911	0.000
ALC18	0.240	0.038	6.315	0.000
MAR18	0.271	0.047	5.775	0.000
SMK18	0.230	0.037	6.252	0.000
CD18	0.341	0.033	10.363	0.000
GAD18	0.431	0.031	13.712	0.000
MDE18	0.514	0.030	17.330	0.000
EAT18	0.374	0.037	10.078	0.000
PTSD18	0.441	0.039	11.290	0.000
PSY18	0.419	0.047	8.917	0.000
PRODA18	0.720	0.030	23.801	0.000
PRODB18	0.674	0.029	23.063	0.000
EXT WITH				
INT	0.000	0.000	999.000	999.000
P	0.000	0.000	999.000	999.000
P WITH				
INT	0.000	0.000	999.000	999.000
Means				
EXT	0.000	0.000	999.000	999.000
INT	0.000	0.000	999.000	999.000
P	0.000	0.000	999.000	999.000
Intercepts				
SMK18	0.414	0.014	29.280	0.000
CD18	0.931	0.018	51.089	0.000
ADHD18	1.350	0.023	57.502	0.000
PSY18	0.145	0.011	12.739	0.000
ALC18	0.670	0.015	43.283	0.000
MAR18	0.244	0.012	20.944	0.000
GAD18	0.527	0.014	36.592	0.000
MDE18	0.609	0.015	39.362	0.000
EAT18	0.513	0.013	39.858	0.000
PTSD18	0.411	0.012	33.051	0.000
PRODA18	0.356	0.012	29.057	0.000
PRODB18	0.323	0.012	27.102	0.000
Variances				

```
    EXT                1.000      0.000    999.000    999.000
    INT                1.000      0.000    999.000    999.000
    P                  1.000      0.000    999.000    999.000

 Residual Variances
    SMK18              0.789      0.034     23.193      0.000
    CD18               0.407      0.049      8.324      0.000
    ADHD18             0.671      0.023     29.776      0.000
    PSY18              0.824      0.039     20.915      0.000
    ALC18              0.781      0.028     27.489      0.000
    MAR18              0.726      0.036     20.197      0.000
    GAD18              0.672      0.037     18.096      0.000
    MDE18              0.337      0.086      3.927      0.000
    EAT18              0.795      0.027     29.127      0.000
    PTSD18             0.774      0.031     24.858      0.000
    PRODA18            0.482      0.044     11.056      0.000
    PRODB18            0.546      0.039     13.862      0.000

STDY Standardization
                                                    Two-Tailed
                    Estimate       S.E.  Est./S.E.    P-Value

 EXT      BY
    ADHD18             0.380      0.029     13.225      0.000
    ALC18              0.402      0.033     12.015      0.000
    MAR18              0.448      0.044     10.184      0.000
    SMK18              0.398      0.047      8.432      0.000
    CD18               0.691      0.038     18.370      0.000

 INT      BY
    GAD18              0.377      0.054      7.042      0.000
    MDE18              0.632      0.070      9.053      0.000
    EAT18              0.255      0.046      5.518      0.000
    PTSD18             0.177      0.040      4.411      0.000

 P        BY
    ADHD18             0.430      0.027     15.911      0.000
    ALC18              0.240      0.038      6.315      0.000
    MAR18              0.271      0.047      5.775      0.000
    SMK18              0.230      0.037      6.252      0.000
    CD18               0.341      0.033     10.363      0.000
    GAD18              0.431      0.031     13.712      0.000
    MDE18              0.514      0.030     17.330      0.000
    EAT18              0.374      0.037     10.078      0.000
    PTSD18             0.441      0.039     11.290      0.000
    PSY18              0.419      0.047      8.917      0.000
    PRODA18            0.720      0.030     23.801      0.000
    PRODB18            0.674      0.029     23.063      0.000

 EXT      WITH
    INT                0.000      0.000    999.000    999.000
    P                  0.000      0.000    999.000    999.000

 P        WITH
    INT                0.000      0.000    999.000    999.000

 Means
    EXT                0.000      0.000    999.000    999.000
    INT                0.000      0.000    999.000    999.000
    P                  0.000      0.000    999.000    999.000

 Intercepts
    SMK18              0.414      0.014     29.280      0.000
    CD18               0.931      0.018     51.089      0.000
    ADHD18             1.350      0.023     57.502      0.000
    PSY18              0.145      0.011     12.739      0.000
    ALC18              0.670      0.015     43.283      0.000
    MAR18              0.244      0.012     20.944      0.000
    GAD18              0.527      0.014     36.592      0.000
    MDE18              0.609      0.015     39.362      0.000
```

```
    EAT18              0.513      0.013     39.858      0.000
    PTSD18             0.411      0.012     33.051      0.000
    PRODA18            0.356      0.012     29.057      0.000
    PRODB18            0.323      0.012     27.102      0.000

 Variances
    EXT                1.000      0.000    999.000    999.000
    INT                1.000      0.000    999.000    999.000
    P                  1.000      0.000    999.000    999.000

 Residual Variances
    SMK18              0.789      0.034     23.193      0.000
    CD18               0.407      0.049      8.324      0.000
    ADHD18             0.671      0.023     29.776      0.000
    PSY18              0.824      0.039     20.915      0.000
    ALC18              0.781      0.028     27.489      0.000
    MAR18              0.726      0.036     20.197      0.000
    GAD18              0.672      0.037     18.096      0.000
    MDE18              0.337      0.086      3.927      0.000
    EAT18              0.795      0.027     29.127      0.000
    PTSD18             0.774      0.031     24.858      0.000
    PRODA18            0.482      0.044     11.056      0.000
    PRODB18            0.546      0.039     13.862      0.000

STD Standardization
                                                    Two-Tailed
                    Estimate       S.E.  Est./S.E.    P-Value

 EXT      BY
    ADHD18             1.632      0.128     12.719      0.000
    ALC18              0.674      0.062     10.818      0.000
    MAR18              0.616      0.082      7.481      0.000
    SMK18              0.645      0.080      8.038      0.000
    CD18               1.576      0.095     16.573      0.000

 INT      BY
    GAD18              0.811      0.118      6.869      0.000
    MDE18              1.879      0.212      8.861      0.000
    EAT18              0.225      0.043      5.283      0.000
    PTSD18             0.586      0.134      4.358      0.000

 P        BY
    ADHD18             1.843      0.122     15.166      0.000
    ALC18              0.402      0.069      5.869      0.000
    MAR18              0.373      0.074      5.060      0.000
    SMK18              0.373      0.063      5.883      0.000
    CD18               0.779      0.081      9.638      0.000
    GAD18              0.927      0.072     12.816      0.000
    MDE18              1.526      0.096     15.871      0.000
    EAT18              0.330      0.036      9.209      0.000
    PTSD18             1.463      0.158      9.276      0.000
    PSY18              0.128      0.025      5.048      0.000
    PRODA18            0.552      0.040     13.840      0.000
    PRODB18            0.515      0.038     13.482      0.000

 EXT      WITH
    INT                0.000      0.000    999.000    999.000
    P                  0.000      0.000    999.000    999.000

 P        WITH
    INT                0.000      0.000    999.000    999.000

 Means
    EXT                0.000      0.000    999.000    999.000
    INT                0.000      0.000    999.000    999.000
    P                  0.000      0.000    999.000    999.000

 Intercepts
    SMK18              0.672      0.044     15.380      0.000
    CD18               2.124      0.061     34.831      0.000
```

ADHD18	5.789	0.105	54.983	0.000
PSY18	0.044	0.007	6.312	0.000
ALC18	1.125	0.042	26.781	0.000
MAR18	0.335	0.034	9.757	0.000
GAD18	1.134	0.052	22.005	0.000
MDE18	1.810	0.072	25.031	0.000
EAT18	0.453	0.022	20.714	0.000
PTSD18	1.365	0.082	16.737	0.000
PRODA18	0.273	0.018	15.100	0.000
PRODB18	0.247	0.018	13.690	0.000
Variances				
EXT	1.000	0.000	999.000	999.000
INT	1.000	0.000	999.000	999.000
P	1.000	0.000	999.000	999.000
Residual Variances				
SMK18	2.073	0.170	12.176	0.000
CD18	2.117	0.256	8.284	0.000
ADHD18	12.337	0.464	26.594	0.000
PSY18	0.077	0.018	4.194	0.000
ALC18	2.202	0.129	17.042	0.000
MAR18	1.372	0.144	9.558	0.000
GAD18	3.111	0.192	16.190	0.000
MDE18	2.974	0.750	3.963	0.000
EAT18	0.619	0.044	13.961	0.000
PTSD18	8.528	0.536	15.901	0.000
PRODA18	0.283	0.029	9.788	0.000
PRODB18	0.319	0.031	10.150	0.000

R-SQUARE

Observed Variable	Estimate	S.E.	Est./S.E.	Two-Tailed P-Value
SMK18	0.211	0.034	6.212	0.000
CD18	0.593	0.049	12.147	0.000
ADHD18	0.329	0.023	14.631	0.000
PSY18	0.176	0.039	4.458	0.000
ALC18	0.219	0.028	7.691	0.000
MAR18	0.274	0.036	7.627	0.000
GAD18	0.328	0.037	8.818	0.000
MDE18	0.663	0.086	7.740	0.000
EAT18	0.205	0.027	7.500	0.000
PTSD18	0.226	0.031	7.243	0.000
PRODA18	0.518	0.044	11.900	0.000
PRODB18	0.454	0.039	11.531	0.000

SUMMARY OF FACTOR SCORES

FACTOR SCORE INFORMATION (COMPLETE-DATA PATTERN)
FACTOR DETERMINACIES

EXT	0.797
INT	0.725
P	0.870

FACTOR SCORE INFORMATION (PATTERN 2)
FACTOR DETERMINACIES

EXT	0.776
INT	0.677
P	0.739

FACTOR SCORE INFORMATION (PATTERN 3)
FACTOR DETERMINACIES

EXT	0.796
INT	0.724
P	0.865

```
FACTOR SCORE INFORMATION (PATTERN 4)
      FACTOR DETERMINACIES

      EXT        0.797
      INT        0.720
      P          0.868

FACTOR SCORE INFORMATION (PATTERN 5)
      FACTOR DETERMINACIES

      EXT        0.796
      INT        0.469
      P          0.865

FACTOR SCORE INFORMATION (PATTERN 6)
      FACTOR DETERMINACIES

      EXT        0.787
      INT        0.723
      P          0.866

FACTOR SCORE INFORMATION (PATTERN 7)
      FACTOR DETERMINACIES

      EXT        0.667
      INT        0.725
      P          0.869

FACTOR SCORE INFORMATION (PATTERN 8)
      FACTOR DETERMINACIES

      EXT        0.621
      INT        0.724
      P          0.869

FACTOR SCORE INFORMATION (PATTERN 9)
      FACTOR DETERMINACIES

      EXT        0.643
      INT        0.664
      P          0.702

FACTOR SCORE INFORMATION (PATTERN 10)
      FACTOR DETERMINACIES

      EXT        0.559
      INT        0.425
      P          0.853

FACTOR SCORE INFORMATION (PATTERN 11)
      FACTOR DETERMINACIES

      EXT        0.611
      INT        0.654
      P          0.672

FACTOR SCORE INFORMATION (PATTERN 12)
      FACTOR DETERMINACIES

      EXT        0.784
      INT        0.725
      P          0.870

FACTOR SCORE INFORMATION (PATTERN 13)
      FACTOR DETERMINACIES

      EXT        0.769
      INT        0.725
      P          0.869
```

```
     Beginning Time:  10:07:10
        Ending Time:  10:07:11
       Elapsed Time:  00:00:01
```


MUTHEN & MUTHEN
3463 Stoner Ave.
Los Angeles, CA 90066

Tel: (310) 391-9971
Fax: (310) 391-8971
Web: www.StatModel.com
Support: Support@StatModel.com

Copyright (c) 1998-2021 Muthen & Muthen
